# JUND/linc00976 promotes cholangiocarcinoma progression and metastasis, inhibits ferroptosis by regulating the miR-3202/GPX4 axis

**DOI:** 10.1038/s41419-022-05412-5

**Published:** 2022-11-18

**Authors:** Shan Lei, Wenpeng Cao, Zhirui Zeng, Zhixue Zhang, Bangming Jin, Qianting Tian, Yingming Wu, Tuo Zhang, Dahuan Li, Chujiao Hu, Jinzhi Lan, Jinjuan Zhang, Tengxiang Chen

**Affiliations:** 1 Department of Physiology, School of Basic Medical Sciences, Guizhou Medical University, Guiyang, 550009 Guizhou China; 2 Transformation Engineering Research Center of Chronic Disease Diagnosis and Treatment, Guizhou Medical University, Guiyang, 550009 Guizhou China; 3 Department of Anatomy, School of Basic Medicine, Guizhou Medical University, Guiyang, 550009 Guizhou China; 4 Digestive Endoscopy Center, the Affiliated of Guizhou Medical University, Guiyang, 550009 Guizhou China; 5 State Key Laboratory of Functions and Applications of Medicinal Plants, Guizhou Medical University, Guiyang, 550009 Guizhou China; 6 The Functional Science laboratory, School of Basic Medical Sciences, Guizhou Medical University, Guiyang, 550009 Guizhou China

**Keywords:** Long non-coding RNAs, Oncogenes

## Abstract

Long noncoding RNAs (lncRNAs) are a novel class of noncoding RNAs that have emerged as critical regulators and biomarkers in various cancers. Nevertheless, the expression profile and mechanistic function of lncRNAs in cholangiocarcinoma (CCA) remain unclear. Herein, we examined the expression levels of linc00976 in clinical specimens and cell lines using reverse transcription-quantitative PCR. In total, 50 patients with CCA were enrolled to analyze the correlation between linc00976 expression and clinical characteristics of CCA. Loss- and gain-of-function experiments were performed to investigate the biological effects of linc00976 on proliferation, ferroptosis, migration, and invasion of CCA cells in vitro and in vivo. In situ hybridization, RNA immunoprecipitation, bioinformatic databases, RNA pull-down assay, a dual-luciferase reporter assay, mRNA sequencing, chromatin immunoprecipitation–PCR, and rescue experiments were performed to elucidate the underlying mechanisms of linc00976-induced competitive endogenous RNA regulatory networks. We characterized a novel and abundant lncRNA, linc00976, that functions as a pro-oncogenic regulator of CCA progression. Compared with normal controls, linc00976 was dramatically upregulated in CCA tissue samples and cell lines. Patients with CCA exhibiting high linc00976 expression had a highly advanced clinical stage, substantial lymph node metastasis, and poor overall survival. Knockdown of linc00976 significantly repressed proliferation and metastasis and promoted ferroptosis of CCA cells both in vitro *and* in vivo, whereas linc00976 overexpression exerted the opposite effect. Mechanistically, linc00976 competitively interacted with miR-3202 to upregulate GPX4 expression, thus contributing to the malignant biological behavior of CCA cells. Moreover, we demonstrated that JUND specifically interacts with the linc00976 promoter and activates linc00976 transcription. Accordingly, JUND promotes linc00976 transcription, and linc00976 plays a crucial role in accelerating CCA tumorigenesis and metastasis and inhibiting ferroptosis by modulating the miR-3202/GPX4 axis. These findings suggest that targeting linc00976 may afford a promising therapeutic strategy for patients with CCA.

## Introduction

Cholangiocarcinoma (CCA) is responsible for the fourth-highest number of tumor-related deaths worldwide [1]. Although therapeutic options for patients with CCA have improved, the 5-year survival rate for patients with CCA remains <5% [2]. The poor prognosis of patients with CCA can be attributed to the high proliferation and early invasion of cancer cells [3]. Therefore, there is an urgent need to elucidate the molecular mechanisms underlying CCA progression.

Long noncoding RNAs (lncRNAs) are a type of RNAs with a sequence length of >200 nucleotides, but lack coding function [4]. To date, numerous studies have revealed that aberrant lncRNA expression can be associated with a series of biological processes, including cell cycle processes, apoptosis, metastasis, aging, and chemotherapy resistance [5, 6]. Moreover, lncRNAs can act as oncogenes or tumor suppressors that affect cancer progression by regulating downstream targets and relative signal pathways [7]. Xu et al. have revealed that SPRY4-IT1 acts as an oncogenic lncRNA that promotes CCA cell proliferation by scaffolding EZH2/LSD1/DNMT1 and sponging miR-101-3p [8]. Chen et al. have reported that lncRNA PVT1 can promote CCA proliferation and metastasis by silencing ANGPTL4 expression [9]. Previously, we have shown that linc00976 promotes PC progression through OTUD7B by sponging miR-137 via the EGFR/MAPK pathway [10]. Typically, abnormal lncRNA expression can be associated with the occurrence and progression of tumors via the competing endogenous RNA (ceRNA) regulating network, post-transcriptional regulation, metabolism, and reprogramming [11, 12]. Currently, the ceRNA regulation network is the most frequently examined molecular mechanism to clarify lncRNA function in tumors; as a “sponge body,” lncRNA can increase or decrease the expression of downstream target genes by sponging target miRNAs and plays a role in tumorigenesis and development [13, 14].

Herein, we found that linc00976 was significantly overexpressed in CCA and correlated with clinicopathological features in patients with CCA. The experimental results indicated that linc00976 exerted pro-oncogenic effects on CCA proliferation and metastasis by targeting the miR-13202/GPX4 axis. Our findings suggest that linc00976 is a potential therapeutic target for CCA.

## Results

### The expression of linc00976 and its correlation with the clinical parameters in patients with CCA

**Fig. 1:**
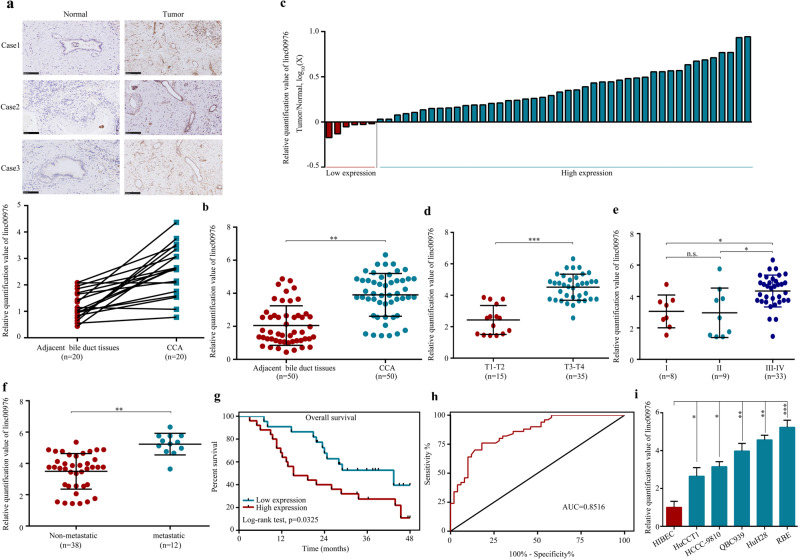
Correlation between linc00976 expression and clinical characteristics of CCA. **a** ISH analysis of linc00976 expression in CCA and adjacent non-tumor tissues. **b** The expression level of linc00976 was detected by RT-qPCR in 50 paired CCA tissues and adjacent non-cancerous tissues. **c** The fold changes of linc00976 expression in each paired sample are arranged from high to low. **e** Correlation between linc00976 expression and clinical stage. **d** Correlation between linc00976 expression and tumor size. **f** Correlation between linc00976 expression and distant metastasis. **g** CCA cases were divided into two groups according to the median value of linc00976 expression. Overall survival was analyzed by Kaplan–Meier survival analysis using the log-rank test. **h** ROC curve showing the diagnostic sensitivity and specificity of linc00976 in CCA. i. RT-qPCR analysis of relative expression levels of linc00976 in CCA cell lines and HIBECs. n.s. not significant, *P < 0.05, **P < 0.01, ***P < 0.001. *CCA* cholangiocarcinoma, *HIBECs* human intrahepatic biliary epithelial cells, *ISH* in situ hybridization, *RT-qPCR* reverse transcription-quantitative PCR.

We examined the expression level of linc00976 in 50 human CCA tissues and adjacent non-cancerous tissues. Based on in situ hybridization (ISH) and reverse transcription-quantitative PCR (RT-qPCR) analysis, we found that linc00976 expression was higher in tumor tissues than in normal tissues (Fig. 1a-b). Among these paired clinical samples, 50 cases (90%) exhibited higher linc00976 expression in tumor tissues than in matched non-cancerous tissues (Fig. 1c). To further validate the effect of linc00976 on CCA, the correlation between linc00976 expression and the clinicopathological parameters of patients with CCA was analyzed. The results revealed that linc00976 expression in CCA tissues was positively and significantly associated with the tumor stage (Fig. 1d), TNM stage (Fig. 1e), and lymph node metastasis (Fig. 1f). No significant differences were detected in other parameters, including age, sex, tumor site, and differentiation grade (Table 1). Next, all patients with CCA were divided into two groups, high and low linc00976 expression, based on the median value of linc00976 expression. Kaplan–Meier survival analysis followed by a log-rank test confirmed that patients with higher linc00976 expression levels had a worse overall survival rate than those with lower linc00976 levels (Fig. 1g). Receiver operating curve (ROC) analysis was conducted to determine the diagnostic value of linc00976 based on 50 paired tissue samples. As shown in Fig. 1h, the area under the ROC curve (AUC) was 0.8516, indicating that linc00976 has diagnostic potential in CCA. RT-qPCR revealed that linc00976 expression in CCA cell lines (HuCCT1, HCCC-9810, QBC939, HuH28, and RBE) was significantly elevated when compared with that in human intrahepatic biliary epithelial cells (HIBEC) (Fig. 1f).

**Table 1:** Correlation between clinicopathological characteristics and linc00976 expression levels.

Linc00976 expression					
Features	*n*	Low	High	*X*^2^	*P* value
All cases	50	25	25		
Age				0.333	0.564
<60	30	16	14		
≥60	20	9	11		
Gender				0.739	0.39
Man	29	13	16		
Female	21	12	9		
Tumor stage				11.524	0.001
T1-T2	15	13	2		
T3-T4	35	12	23		
TNM stage				7.219	0.007
I and II	17	13	4		
III and IV	33	12	21		
Tumor site				4.5	0.034
Intrahepatic	10	8	2		
Extrahepatic	40	17	23		
Differentiation grade				3.388	0.66
Well/moderately	9	7	2		
Poorly/undifferentiated	41	18	23		
Lymph node metastasis				3.947	0.047
No	38	22	16		
Yes	12	3	9		

### Knockdown of linc00976 reduces CCA cells progression in vitro and in vivo

**Fig. 2:**
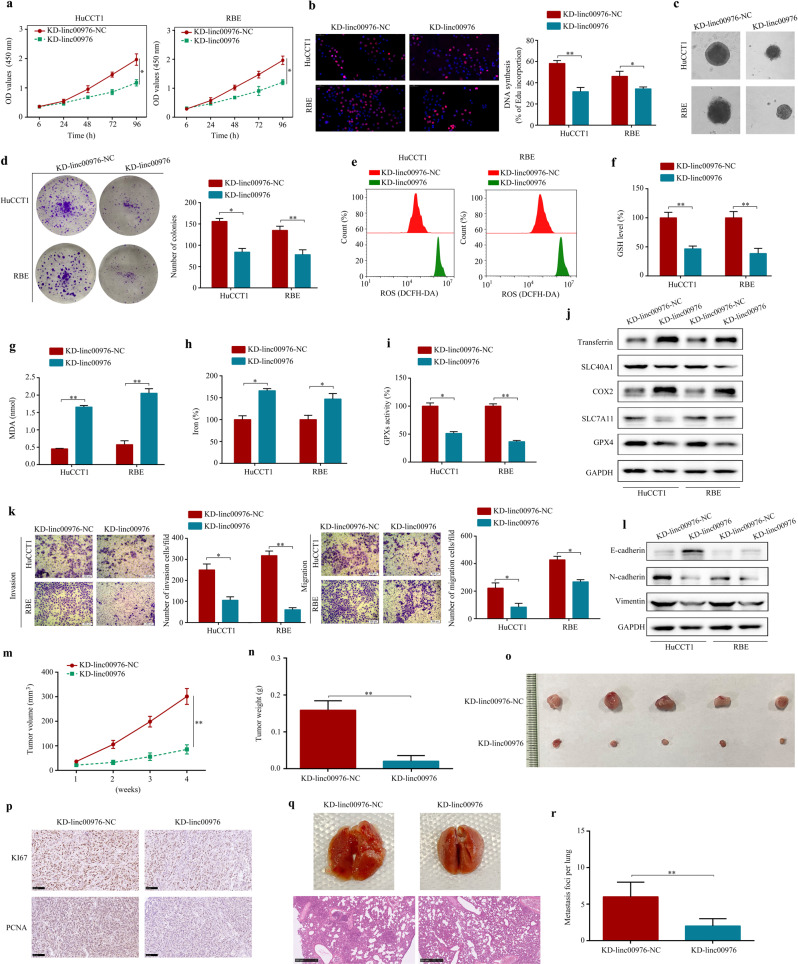
Knockdown of linc00976 inhibits the proliferation and metastasis and induces ferroptosis of CCA cells. **a**–**d** CCK8, EdU (scale bar, 50 μm), generation of CCA spheroids, and colony formation assays detected the effect of linc00976 knockdown on cell proliferation ability. **e**–**i** Effects of linc00976 knockdown on ROS, GSH, MDA, iron progression, and GPX activities in CCA cells. **j** Western blotting detected the effect of linc00976 knockdown on the expression of ferroptosis-associated biomarkers. **k** Transwell migration and Matrigel invasion assays examined the effect of linc00976 knockdown on cell migration and invasion. Scale bar, 50 μm. **l** Western blotting detected the effect of linc00976 knockdown on the expression of EMT-associated biomarkers. **m**, **n** Effect of linc00976 knockdown on tumor growth and tumor weight in nude mice. **o** Representative images of xenograft tumors in nude mice after subcutaneous implantation of HuCCT1 cells stably infected with linc00976-knockdown lentivirus or control lentivirus. **p** Tumor tissues were sectioned and stained for KI67 and PCNA by IHC. Scale bar, 100 μm. n.s. not significant, **q**, **r** Representative images of lung tissues and sections stained with H&E in the lung metastatic mice model. Scale bar, 500 μm. ^*^P < 0.05, ^**^P < 0.01. *CCA* cholangiocarcinoma, *EMTR* epithelial–mesenchymal transition, *GPX* glutathione peroxidase, *GSH* glutathione, *H&E* hematoxylin and eosin, *IHC* immunohistochemistry, *MDA* malondialdehyde, *ROS* reactive oxygen species.

Given that linc00976 is knockdown in CCA tissues and cell lines, we investigated the role of linc00976 in CAA. Stable linc00976 knockdown (KD-linc00976) HuCCT1 and RBE stable cell lines were constructed by lentiviral transfection (Additional file 2: Fig. S1a). Linc00976 knockdown significantly repressed the CCK8 assay, DNA synthesis, generation of CCA spheroids, and colony formation abilities of HuCCT1 and RBE cells (Fig. 2a–d). Furthermore, KD-linc00976 CCA cells exhibited high baseline reactive oxygen species (ROS) levels (Fig. 2e). KD-linc00976 CCA cells exhibited enhanced ferroptosis-related events, including glutathione (GSH) depletion, increased malondialdehyde (MDA) production, and elevated iron levels (Fig. 2f–h). Consistently, KD-linc00976 CCA cells exhibited a low baseline GSH peroxidase (GPX) activity (Fig. 2i). Using western blotting (Fig. 2g), we observed that linc00976 knockdown decreased protein levels of SLC7A11, SLC40A1, and GPX4, and increased those of cyclooxygenase 2 (COX2) and transferrin, suggesting that linc00976 might regulate ferroptosis-associated genes to affect CCA development. In addition, transwell assay results revealed that migration and invasion capacities of linc00976-knockdown cells were significantly reduced (Fig. 2k). Western blotting (Fig. 2i) revealed that linc00976 knockdown could decrease protein levels of N-cadherin and vimentin and increase those of E-cadherin, suggesting that linc00976 might regulate epithelial–mesenchymal transition (EMT)-associated genes, thereby impacting CCA development. Considering in vivo experiments, nude mice were administered stable KD-linc00976 lentivirus- or control lentivirus-transfected HuCCT1 cells subcutaneously (for the subcutaneous xenograft tumor model) or intravenously via the tail vein (for the lung metastatic model). Compared with the control group, tumor volume and weight were significantly reduced in mice injected with linc00976-knockdown cells (Fig. 2m–o). Immunohistochemistry (IHC) revealed that linc00976 knockdown triggered a reduction in KI67 and proliferating cell nuclear antigen (PCNA) protein expression in excised tumor tissues (Fig. 2l). Additionally, the number of metastatic pulmonary colonies was reduced (Fig. 2q, r).

### Overexpression of linc00976 promotes CCA cell progression in vitro and in vivo

**Fig. 3:**
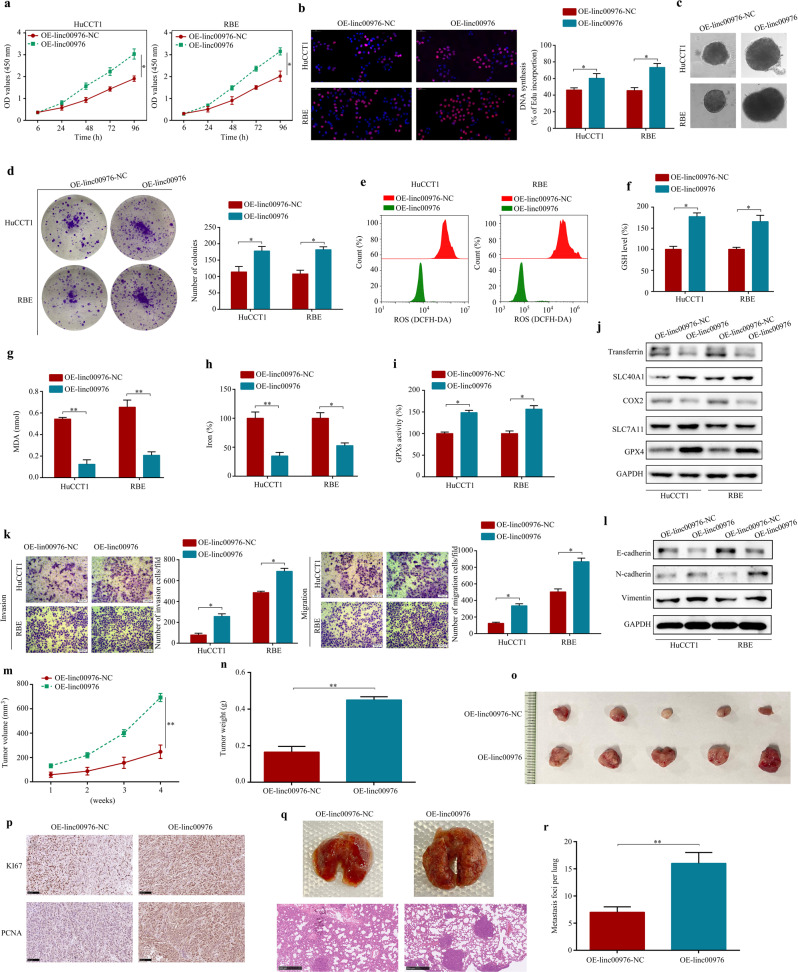
Overexpression of linc00976 promotes proliferation and metastasis and inhibits ferroptosis of CCA cells. **a**–**d** CCK8, EdU (scale bar, 50 μm), generation of CCA spheroids, and colony formation assays detected the effect of linc00976 overexpression on cell proliferation ability. **e**–**i** Effects of linc00976 overexpression on ROS, GSH, MDA, iron progression, and GPX activities in CCA cells. **j** Western blotting detected the effect of linc00976 overexpression on the expression of ferroptosis-associated biomarkers. **k** Transwell migration and Matrigel invasion assays detected the effect of linc00976 overexpression on cell migration and invasion. Scale bar, 50 μm. **l** Western blotting determined the effect of linc00976 overexpression on the expression of EMT-associated biomarkers. **m**, **n** Effect of linc00976 overexpression on tumor growth and tumor weight in nude mice. **o** Representative images of xenograft tumors in nude mice after subcutaneous implantation of HuCCT1 cells stably infected with linc00976-overexpression lentivirus or control lentivirus. **p** Tumor tissues were sectioned and stained for KI67 and PCNA by IHC. Scale bar, 100 μm. **q**, **r** Representative images of lung tissues and lung sections stained with H&E in the lung metastatic mice model. Scale bar, 500 μm. ^*^P < 0.05, ^**^P < 0.01. *CCA* cholangiocarcinoma, *EMT* epithelial–mesenchymal transition, *GPx* glutathione peroxidase, *GSH* glutathione, *H&E* hematoxylin and eosin, *IHC* immunohistochemistry, *MDA* malondialdehyde, *ROS* reactive oxygen species.

HuCCT1 and RBE cell lines were transfected with the linc00976-overexpression or control vectors, and the overexpression efficiency was verified by RT-qPCR (Additional file 2: Fig. S1b). Based on findings of the CCK8 assay, DNA synthesis, CCA spheroid generation, and colony formation, linc00976 overexpression significantly suppressed ferroptosis and promoted the proliferation of CCA cells (Fig. 3a–d). Furthermore, linc00976 overexpression in CCA cells resulted in lower baseline ROS levels (Fig. 3e). In CCA cells, linc00976 overexpression decreased ferroptosis-related events, including GSH depletion, increased MDA production, and elevated iron levels (Fig. 3f–h). Consistently, linc00976 overexpressing CCA cells exhibited high baseline GPX activity (Fig. 3i). Western blotting (Fig. 3g) revealed that linc00976 overexpression increased protein levels of SLC7A11, SLC40A1, and GPX4, and decreased those of COX2 and transferrin. Consistently, transwell assay results demonstrated that linc00976 overexpression significantly promoted CCA cell migration and invasion (Fig. 3k). Using western blotting (Fig. 3i), we observed that linc00976 overexpression increased protein levels of N-cadherin and vimentin and decreased those of E-cadherin. For in vivo experiments, nude mice were administered stable OV-linc00976 lentivirus- or control lentivirus-transfected HuCCT1 cells subcutaneously (for the subcutaneous xenograft tumor model) or intravenously via the tail vein (for the lung metastatic model). Compared with the control group, tumor volume and weight were significantly increased in mice injected with linc00976-overexpression cells (Fig. 3m–o). IHC revealed that linc00976 overexpression increased KI67 and PCNA protein expression in excised tumor tissues (Fig. 3l). Additionally, the number of metastatic pulmonary colonies was elevated (Fig. 3q, r). Collectively, these findings suggested that linc00976 played a critical role in regulating the oncogenic and metastatic capacity of CCA cells.

### Linc00976 acts as a sponge for miR-3202

**Fig. 4:**
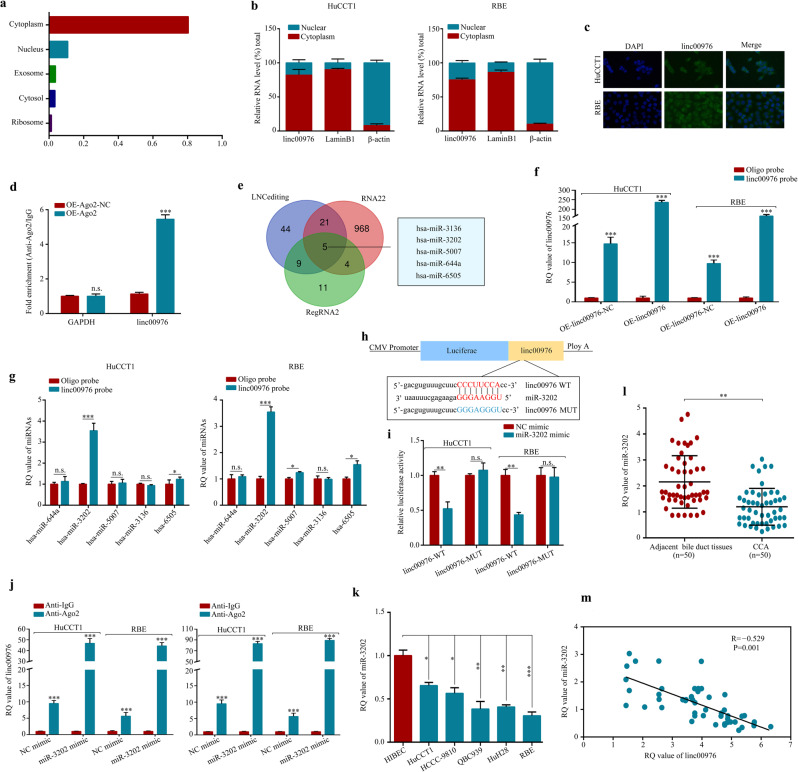
linc00976 acts as a sponge for miR-3202. **a**–**c** lncLocator, nuclear-cytoplasmic fractionation, and ISH assays revealed the subcellular location of linc00976 in CCA cells. **d** RIP assay for linc00976 levels in HuCCT1 cells transfected with Ago2-overexpression or control vectors. **e** Schematic illustration showing overlapping target miRNAs of linc00976 predicted by LNCediting, RegRNA2, and RNA22. **f** Lysates prepared from CCA cells transfected with linc00976-overexpression vectors or control vectors were subject to RNA pull-down assay, and the efficiency was confirmed by RT-qPCR. **g** The relative levels of five miRNA candidates in the CCA cell lysates were examined by RT-qPCR. **h** The putative binding sites between miR-3202 and linc00976. **i** The luciferase activities was detected in CCA cells after co-transfection with linc00976-WT or linc00976-MUT and miR-3202 mimics or NC mimics. **j** Anti-Ago2 RIP assay was conducted in CCA cells after transfection with miR-3202 mimics or NC mimics, followed by western blotting and RT-qPCR analyses to detect the expression levels of Ago2, linc00976, and miR-3202. **k** Relative expression level of miR-3202 in CCA cells and human intrahepatic biliary epithelial cells. n.s. not significant, ^*^P < 0.05, ^**^P < 0.01, ^***^P < 0.001. **l** The expression level of miR-3202 was detected by qRT-PCR in 50 paired CCA tissues and adjacent non-tumor tissues. **m** Pearson correlation analysis of linc00976 and miR-3202 expression in 50 CCA tissues. *CCA* cholangiocarcinoma, *ISH* in situ hybridization, *RT-qPCR* reverse transcription-quantitative PCR.

LncLocator, nuclear–cytoplasmic fractionation, and FISH assays were performed to examine the subcellular localization of linc00976. Herein, we found that linc00976 was predominantly localized in the cytoplasm (Fig. 4a–c). Accumulating evidence has demonstrated that lncRNAs possess abundant miRNA binding sites and function as miRNA sponges to regulate the expression of downstream genes. Given that linc00976 is predominantly localized in the cytoplasm and exhibits marked stability, we hypothesized that linc00976 might serve as a miRNA sponge. The RNA immunoprecipitation (RIP) assay for Ago2 was performed by transfecting HuCCT1 cells with the Ago2-overexpressing plasmid and control vector. Compared with the control group, endogenous linc00976 pull-down by anti-Argonaute2 (anti-Ago2) antibodies was significantly enriched in the Ago2 overexpression group, as determined by RT-qPCR analysis (Fig. 4d), indicating that linc00976 might interact with miRNAs via the Ago2 protein. Next, five miRNA candidates were identified by overlapping the prediction results from three bioinformatic databases (LNCediting, RegRNA2, and RNA22) (Fig. 4e). To further confirm whether linc00976 could directly bind to these miRNA candidates, a pull-down assay with a specific biotin-labeled probe against linc00976 was performed. As shown in Fig. 4f and g, the pull-down efficiency was markedly enhanced after linc00976 overexpression, and only miR-3202 was significantly enriched by the biotinylated linc00976 probe in both HuCCT1 and RBE cell lines. After verifying the transfection efficiencies of miR-3202 mimics and inhibitors (Additional file 2: Fig. S2), the dual-luciferase reporter assay consistently revealed that miR-3202 mimics could inhibit the luciferase activity of the wild-type reporter for linc00976 (linc00976-WT vector), in contrast to the mutant-type reporter vector (linc00976-MUT vector) (Fig. 4h, i). Moreover, an anti-Ago2 RIP assay revealed that Ago2, linc00976, and miR-3202 were all efficiently pulled down in the presence of anti-Ago2 antibodies, but not anti-IgG antibodies. Compared with the NC mimic group, linc00976 and miR-3202 were significantly enriched in HuCCT1 and RBE cells transfected with miR-3202 mimics (Fig. 4j). CCA cell lines exhibited lower miR-3202 expression than HIBECs (Fig. 4k). Further, we found that miR-3202 expression was higher in normal tissues than in tumor tissues (Fig. 4l). The miR-3202 expression level was inversely correlated with that of linc00976 (Fig. 4m).

### The pro-oncogenic role of linc00976 in CCA progression depends on miR-3202

**Fig. 5:**
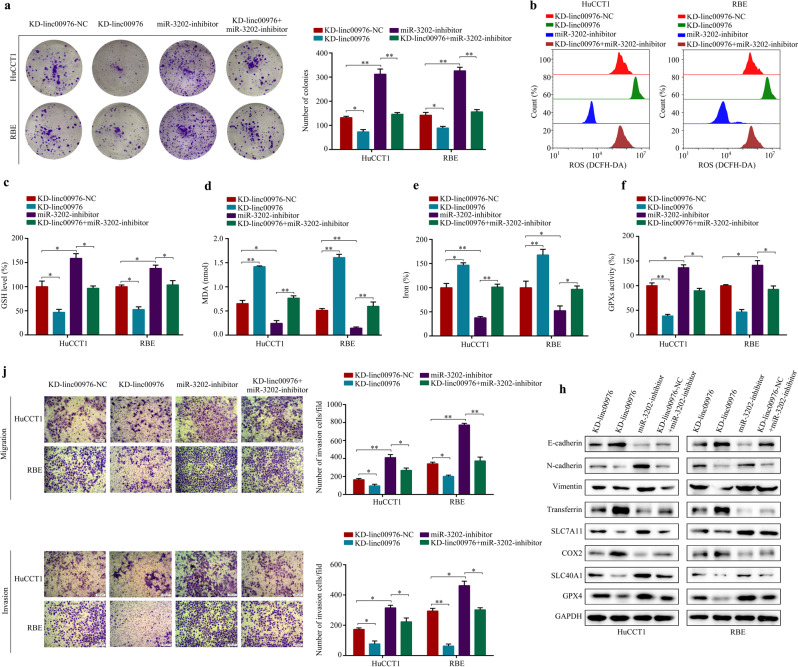
Knockdown of miR-3202 rescues KD-linc00976-mediated inhibitory effect on the proliferation, metastasis, and ferroptosis in CCA cells. **a** CCA cells were transfected with KD-linc00976-NC or KD-linc00976 or co-transfected with KD-linc00976 and miR-3202 inhibitors. Colony formation assays detected the cell proliferation ability of CCA cells in each group. **b**–**f** Effects of miR-3202 silencing on KD-linc00976-mediated ROS, GSH, MDA, iron progression, and GPX activities in CCA cells. **j** Transwell migration and Matrigel invasion assays evaluated the effect of miR-3202 silencing on KD-linc00976-mediated migration and invasion ability. Scale bar, 50 μm. **h** The protein expressions of ferroptosis and EMT-associated biomarkers were analyzed by western blotting and normalized to GAPDH. ^*^P < 0.05, ^**^P < 0.01. *CCA* cholangiocarcinoma, *EMT* epithelial–mesenchymal transition, *GPx* glutathione peroxidase, *GSH* glutathione, *MDA* malondialdehyde, *ROS* reactive oxygen species.

Functional rescue experiments were performed to determine whether linc00976 promotes the proliferation and metastasis of CCA cells by regulating miR-3202 expression. The miR-3202 inhibitors were first transfected into linc00976-knockdown cells to explore the effect of miR-3202 on the linc00976-induced malignant phenotype of CCA. Based on colony formation assay results, knockdown of linc00976 significantly reduced colony formation in both CCA cell lines, whereas miR-3202 silencing attenuated these effects (Fig. 5a). Linc00976 knockdown in CCA cells increased ferroptosis-related events, including GSH depletion, increased MDA production, and elevated iron levels, along with low baseline GPX activities; miR-3202 silencing attenuated these effects (Fig. 5b–f). As shown in Fig. 5j, exogenous downregulation of miR-3202 expression in CCA cells effectively weakened the suppressive effects of linc00976 knockdown on cell migration and invasion. Treatment with miR-3202 inhibitors could rescue the reduced expression levels of ferroptosis-associated proteins (SLC7A11, SLC40A1, and GPX4) and mesenchymal phenotype markers (N-cadherin and vimentin) induced by linc00976 knockdown. In contrast, miR-3202 silencing remarkably reversed the linc00976-mediated increase in expression levels of ferroptosis proteins (COX2 and transferrin) and the epithelial cell marker (E-cadherin) (Fig. 5h). Collectively, these findings revealed that linc00976 promoted the malignant biological behavior of CCA cells, at least, partly, by functioning as a miR-3202 sponge.

### Linc00976 positively regulates GPX4 expression via sponging miR-3202 in CCA cells

**Fig. 6:**
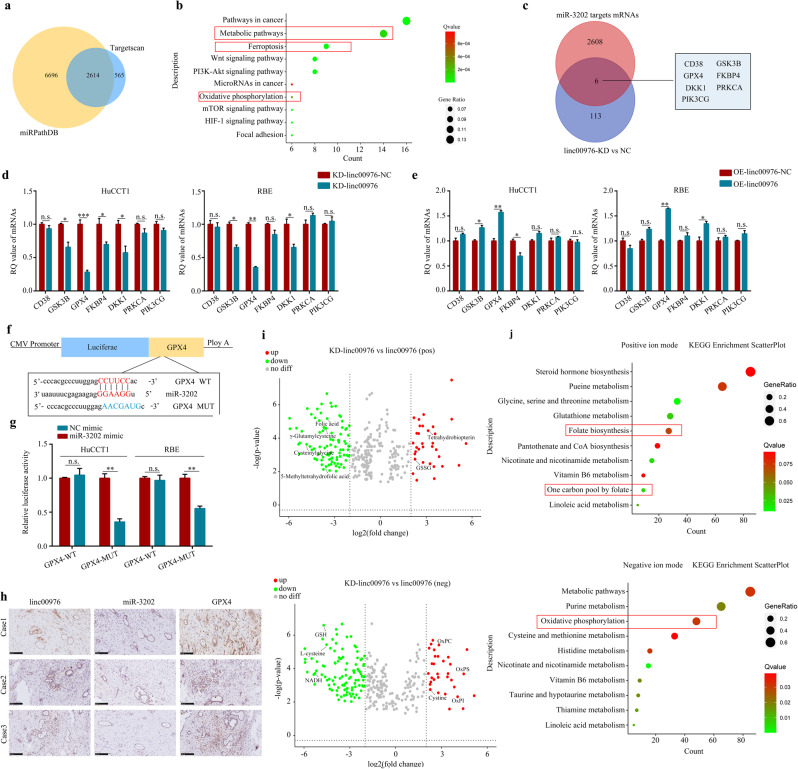
linc00976 positively regulates GPX4 expression via sponging miR-3202 in CCA cells. **a** Schematic illustration showing overlapping of the target mRNAs of miR-3202 predicted by TargetScan and miRPathDB. **b** The downregulated mRNAs were subjected to KEGG enrichment analysis. **c** Venn diagram showing overlapping of target mRNAs and downregulated mRNAs of miR-3202. **d** RT-qPCR showed the mRNA levels of six candidate genes in CCA cells transfected with KD-linc00976 or KD-linc00976-NC. **e** RT-qPCR showed the mRNA levels of six candidate genes in CCA cells transfected with OE-linc00976 or OE-linc00976-NC. **f** The putative binding sites between miR-3202 and GPX4. **g** The luciferase activities were detected in CCA cells after co-transfection with GPX4-WT or GPX4-MUT and miR-3202 mimics or NC mimics. **h** IHC images showed the co-expression of linc00976, miR-3202, and GPX4. Scale bar, 100 μm. **i** Volcano plots demonstrating altered metabolite levels. Metabolites associated with GSH metabolism and folate biosynthesis are significantly downregulated, while oxidized phospholipids are significantly upregulated. **j** KEGG enrichment analysis of differentially abundant metabolites identified in negative and positive ion modes, respectively. n.s. not significant, ^*^P < 0.05, ^**^P < 0.01. *CCA* cholangiocarcinoma, *GSH* glutathione, *IHC* immunohistochemistry, *KEGG* Kyoto Encyclopedia of Genes and Genomes, *RT-qPCR* reverse transcription-quantitative PCR.

Based on the above findings, we further explored whether downstream molecules could be regulated via the linc00976/miR-3202 axis to influence the malignant phenotype of CCA. Previous studies have demonstrated that lncRNAs function as miRNA sponges to terminate their regulatory effects on target genes [10]. Meanwhile, miRNA-targeting mRNA interactions typically lead to degradation or translational repression of target mRNA. Thus, we speculated the presence of a positive correlation between the expression of linc00976 and its target genes. Firstly, 2614 mRNA candidates were identified by overlapping the prediction results from two bioinformatic databases (TargetScan and miRPathDB) (Fig. 6a). We analyzed the mRNA expression profile using mRNA-seq, focusing on differentially expressed genes (DEGs) between HuCCT1 cells with stable linc00976 knockdown and control HuCCT1 cells. In total, 119 DEGs were screened according to the filter criteria of fold change ≤ 1 and *P*-value < 0.05 (Additional file 2: Fig. S3a and b). DEGs were subjected to the Kyoto Encyclopedia of Genes and Genomes (KEGG) enrichment analysis. As shown in Fig. 6b, ferroptosis, metabolic pathways, and oxidative phosphorylation were the most significantly enriched. Second, six mRNA candidates were identified after overlapping the mRNA-seq-identified DEGs (*n* = 119), and the potential target genes of miR-3202 were predicted using bioinformatic databases (*n* = 2614). Subsequently, RT-qPCR analysis was performed to determine the mRNA expression of these six candidate genes in CCA cells. Compared with control cells, linc00976 knockdown significantly altered mRNA levels of GPX4 and GSK3B in CCA cells (Fig. 6d). After introducing exogenous linc00976 into CCA cells, mRNA expression of GPX4 was significantly increased in CCA cells, whereas no significant change in GSK3B mRNA was detected in RBE cells (Fig. 6e).

Next, the putative binding sites of GPX4 on miR-3202 and the corresponding mutant sequences were identified (Fig. 6f). Co-transfection of the GPX4-WT reporter vector with miR-3202 mimics dramatically decreased luciferase activity; this effect was not observed with scrambled oligonucleotides. No significant difference in luciferase activity was detected when the binding sequence was mutated (Fig. 6g). The mRNA expression of GPX4 was also higher in CCA tissues and cells than in the corresponding adjacent non-cancerous tissues and HIBECs (Additional file 2: Fig. S4a and b). Pearson’s correlation analysis revealed that protein and mRNA expression levels of GPX4 in CCA tissues were positively correlated with linc00976 expression and inversely correlated with miR-3202 expression, exhibiting statistical significance (Fig. 6h, Additional file 2: Fig. S3c and d).

Next, we conducted a metabolomic study to examine the impact of linc00976 knockout on metabolite levels in HuCCT1 cells. Based on KEGG enrichment analyses of differentially abundant metabolites, we found that the identified metabolites were associated with oxidative phosphorylation (Fig. 6j), consistent with our RNA-seq results (Fig. 6b). Typically, oxidized phospholipid levels dramatically increased following linc00976 knockout, consistent with an increase in lipid peroxide levels associated with elevated ferroptosis activity (Fig. 6i). Notably, linc00976 knockout markedly reduced GSH activity and enhanced levels of oxidized GSH (GSSG). As key components of the GSH synthesis process [15], γ-glutamyl cysteine, cysteinyl glycine, and cysteine levels were significantly reduced, whereas no significant changes in cystine or glutamic acid levels were detected (Fig. 6i). This finding suggests that the linc00976 knockout specifically disrupted the process of cysteine synthesis. Moreover, levels of key folate biosynthesis-related metabolites, including folic acid and 5-methyltetrahydrofolic acid, were significantly reduced (Fig. 6i). These findings suggested that linc00976 protects CCA cells against the induction of ferroptosis by maintaining GSH metabolism and folate biosynthesis.

### Linc00976 facilitates proliferation and metastasis and inhibits ferroptosis of CCA cells via the miR-3202/GPX4 axis

**Fig. 7:**
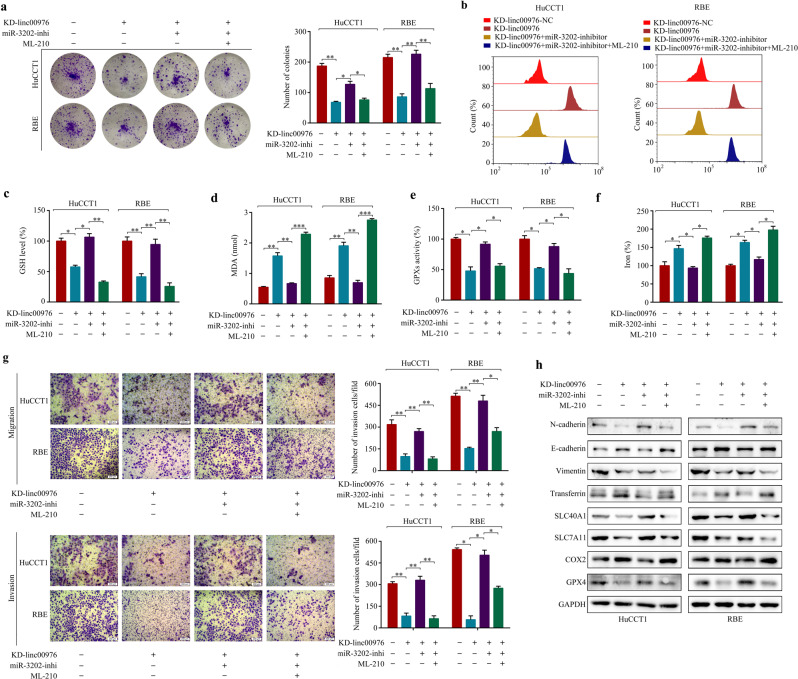
Gain-of-function assays confirm the involvement of linc00976/miR-3202/GPX4 axis in CCA progression. **a** KD-linc00976 stable CCA cells were transfected with miR-3202 inhibitor or co-transfected with miR-3202 inhibitor and ML-210. Colony formation assays evaluated the effect of the linc00976/miR-3202/GPX4 axis on cell proliferation. **b**–**f** Effects of the linc00976/miR-3202/GPX4 axis on ROS, GSH, MDA, iron progression, and GPX activities in CCA cells. **g** Transwell migration and Matrigel invasion assays evaluated the effect of the linc00976/miR-3202/GPX4 axis on the migration and invasion ability of CCA cells. Scale bar, 50 μm. **h** The protein expressions of ferroptosis and EMT-associated were analyzed by western blotting and normalized to GAPDH. ^*^P < 0.05, ^**^P < 0.01, ^***^P < 0.001. *CCA* cholangiocarcinoma, *EMT* epithelial–mesenchymal transition, *GPx* glutathione peroxidase, *GSH* glutathione, *MDA* malondialdehyde, *ROS* reactive oxygen species.

Rescue experiments were performed to determine whether linc00976 promotes the malignant biological functions of CCA cells by competitively binding to miR-3202 and subsequently enhancing GPX4 expression. First, stable linc00976-silenced cell lines were transfected with miR-3202 inhibitors alone or in combination with ML-210, a GPX4 inhibitor. The colony formation assay results were consistent with previous data, revealing that the reduced proliferation of KD-linc00976 stable cells was effectively reversed by miR-3202 inhibitors; co-treatment with ML-210 could significantly reverse these rescue effects of miR-3202 inhibitors on KD-linc00976 stable cells (Fig. 7a). In addition, miR-3202 silencing abolished GSH depletion, increased MDA production, and elevated iron levels, and exhibited low baseline GPX activities following linc00976 knockdown; the reintroduction of ML-210 successfully attenuated these miR-3202 silencing-mediated effects (Fig. 7b–f). Following linc00976 knockdown, CCA cells exhibited increased ferroptosis-related events, including GSH depletion, increased MDA production, and elevated iron levels, along with low baseline GPX activities; miR-3202 silencing attenuated these effects. Transwell assay results revealed that exogenous downregulation of miR-3202 expression in CCA cells markedly suppressed the inhibitory effects of linc00976 knockdown on cell migration and invasion. However, co-transfection with miR-3202 inhibitors and ML-210 restored the suppressive effects of KD-linc00976 on cell motility in stable linc00976-silenced cells (Fig. 7g). Similar trends were observed considering the regulatory effects of the linc00976/miR-3202/GPX4 axis on the expression levels of ferroptosis- and EMT-associated proteins (Fig. 7h).

### Linc00976 was a downstream gene of JUND

**Fig. 8:**
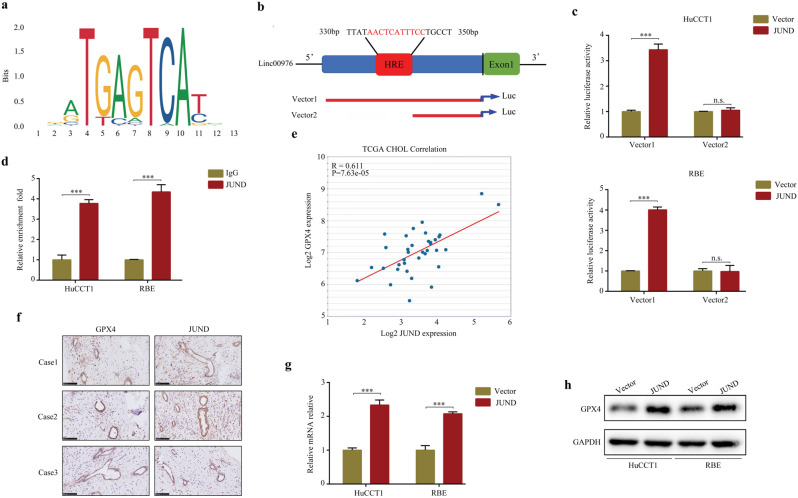
JUND regulates the expression of linc00976 by binding to the JUND promoter. **a** The motif of JUND. **b** The binding site in the promoter of linc00976 is shown. **c** CCA cells were transfected with either a full‐length or truncated linc00976 promoter‐pGL3 reporter vector and further cultured, either with or without JUND plasmid. After 48 h, luciferase activity was measured using the dual‐luciferase reporter assay system. **d** ChIP assays with anti-JUND antibody verifying the binding between JUND and response element of the linc00976 promoter. **e** Co-expression relationship between linc00976 and JUND based on data from CCA tissues. **f** Co-expression relationship between JUND and GPX4 based on the data from our additional CCA tissues. Scale bar, 100 μm. **g**, **h** RT-qPCR and western blotting detected the effect of JUND overexpression on the mRNA and protein expression of GPX4. n.s. not significant, ^***^P < 0.001. *CCA* cholangiocarcinoma, *ChIP* chromatin-immunoprecipitation, *RT-qPCR* reverse transcription-quantitative PCR.

To further investigate the regulatory networks of linc00976. First, we used the online database PROMO to analyze the potential transcription factors of linc00976. Total of 46 potential transcription factors of linc00976 were found. Among them, JUND had the highest co-expression relationship with GPX4 (Additional file 1: Table S1). The motif sequence of JUND was obtained from the JASPAR database (Fig. 8a), and a site on the linc00976 promoter was predicted (Fig. 8b). To verify the predicted results, a chromatin-immunoprecipitation (ChIP) assay was performed using an anti-JUND antibody in CCA cells. Based on assay results, we observed that the site sequence of the linc00976 promoter was enriched by the anti-JUND antibody, and its expression was higher in JUND overexpressing cells (Fig. 8c). According to data from CCA tissues in the TCGA database (*R* = 0.611, *P* < 0.001; Fig. 8e), GPX4 was co-expressed with JUND (Fig. 8f). We found that mRNA and protein levels of GPX4 were markedly increased in JUND overexpressing CCA cells (Fig. 8g, h).

## Discussion

Recently, lncRNAs have received considerable attention in cancer research investigating biological and pathological progress of proliferation, metastasis, tumor metabolism, and chemoresistance [16, 17]. To identify whether linc00976 was abnormally expressed in CCA and determine its effect on prognosis, ISH and RT-qPCR were used to verify linc00976 expression levels in bile duct adjacent/tumor tissues and normal/cancer cell lines, and overall survival analysis was used to evaluate prognosis risk. Functional studies revealed that linc00976 knockdown significantly suppressed cell proliferation, migration, and invasion and induced ferroptosis in vivo and in vitro. These findings suggest that linc00976 might act as an oncogene and play a critical role in the progression of CCA. Herein, we identified the mechanism responsible for upregulated linc00976 expression in CAA cells. We observed that JUND could interact specifically with the linc00976 promoter through the binding site, indicating the mechanism through which JUND upregulated linc00976 expression.

The subcellular distribution of RNA is closely related to its biological function [18]. It is well-established that cytoplasmic lncRNAs act as sponges for miRNAs, and the latter mostly repress translation or degrade mRNAs by binding with Ago2 protein [19, 20]. In the present study, linc00976 was mainly located in the cytoplasm of CCA cells and could recognize and bind to the Ago2 protein, suggesting that linc00976 might exert its regulatory functions via classically harboring miRNAs. Among the four candidate miRNAs predicted by bioinformatic databases, only miR-3202 was further validated to exhibit a high binding capacity with linc00976 in both CCA cell lines using the RNA pull-down, dual-luciferase reporter, and RIP assays. Although the involvement of miR-3202 in the pathogenesis of multiple tumors has been previously reported [21, 22] limited studies have examined the function and mechanism of both miR-3202 and lincRNAs. Our study revealed that miR-3202 was markedly downregulated in CCA tissues and was inversely correlated with linc00976 expression. Moreover, functional rescue experiments confirmed that miR-3202 inhibitors significantly reversed the inhibitory effects of linc00976 depletion on CCA cell proliferation and metastasis, whereas miR-3202 mimics attenuated the facilitatory effects of linc00976 overexpression.

Next, we explored miR-3202 targets. Herein, we, for the first time, reported that GPX4 is a downstream molecule of miR-3202 in CCA. Consistent with the theory of ceRNA, our current study revealed a positive correlation between the expression of linc00976 and GPX4 and a negative correlation between the expression of miR-3202 and GPX4 in clinical CCA tissues. Bioinformatic analysis and subsequent functional validation revealed that linc00976 upregulated GPX4 expression by sponging miR-3202 to accelerate the progression of CCA, including suppression of ferroptosis and promotion of proliferation, migration, and invasion. Recently, ferroptosis has been described as a form of cell death owing to the accumulation of lipid ROS to toxic levels, involving an oxidative, iron-dependent process. It is well-documented that ferroptosis plays a role in several pathological conditions, including cancer, neurodegeneration, and ischemia-reperfusion injury [23–25]. Ferroptosis can reportedly regulate the initiation, development, invasion, metastasis, and therapeutic resistance of multiple cancer types [26–28]. GPX4 is a key regulator of ferroptosis that inhibits COX and lipoxygenase (LOX) activities by reducing lipid peroxidation levels in cancer cells [29, 30]. However, the potentially important role of ferroptosis in metastatic progression in CCA remains poorly clarified. In addition to GPX4 inhibition, intracellular GSH depletion is another key driver of ferroptosis induction [31]. GSH functions as a co-factor that enables GPX4 to eliminate lipid peroxides from cells [32]. GSH synthesis depends on system Xc- by exchanging extracellular cystine for intracellular glutamate. Cystine is reduced to cysteine, the most important intermediate metabolite involved in GSH synthesis [33]. We demonstrated that linc00976 could sustain GSH synthesis by maintaining the reduction of cystine to cysteine. This linc00976-dependent cysteine synthesis may occur, in part, via the TrxR/Trx system, comprising selenocysteine-dependent antioxidant enzymes. Notably, we also found that linc00976 depletion markedly reduced folate biosynthesis. Our comprehensive RNA-seq and metabolomics analyses revealed that mRNA levels were significantly correlated with levels of metabolites involved in folate biosynthesis, suggesting that linc00976 may regulate this metabolic process at the transcriptional level. Recent studies have demonstrated that the availability of tetrahydrobiopterin (BH4) plays a key protective role in controlling cellular responses to ferroptosis upon GPX4 inhibition. We postulated that linc00976 could protect CCA cells against ferroptosis induction in a GPX4-independent manner by regulating folate synthesis at the transcriptional level.

In the present study, we aimed to identify the role and molecular mechanism of action of linc00976 in CCA. We observed that linc00976 was highly expressed in CCA and positively associated with adverse clinical features. In addition, linc00976 promoted CCA cell proliferation and motility and inhibited ferroptosis via the miR-3202/GPX4 axis. Accordingly, linc00976 may be a potential target for CCA diagnosis and therapy.

## Materials and methods

### Patient data and tissue sample

CCA and adjacent non-tumor tissues were obtained from 50 patients who underwent surgical treatment between 2016 and 2020 at the Affiliated Hospital of Guizhou Medical University, Guizhou, China. All tissues were confirmed by histopathological examination, snap-frozen in liquid nitrogen immediately after surgical resection, and stored at −80 °C. No patient had received any local or systemic therapy before surgery. All registered patients were informed of the study, and consent was obtained. All experiments were approved by the research ethics committee of Guizhou Medical University.

### Cell culture and transfection

The HIBEC, HuCCT1, HCCC-9810, QBC939, HuH28, and RBE cell lines were purchased from ATCC (Manassas, VA, USA). All cells were grown in RPMI-1640 medium (Gibco, USA) medium supplemented with 10% fetal bovine serum (FBS; Gibco, USA) and 1% penicillin/streptomycin (Solarbio, Beijing, China) at 37 °C and 5% CO_2_. All the cell lines had been authenticated through STR profiling and confirmed to be mycoplasma free.

### RT-qPCR and Western Blot

RT-qPCR and western blotting analyses were performed as previously described [10]. The RT-qPCR reagents used in the experiments were purchased from Takara (Shiga, Japan). The primer sequences used for RT-qPCR are listed in Table S2. The primary and secondary antibodies used for western blotting against E-cadherin (Cat No. 14472), N-cadherin (Cat No. 13116), vimentin (Cat No. 46173), SLC7A11 (Cat No. 98051), COX2 (Cat No. 12282), GPX4 (Cat No. 59735), JUND (Cat No. 5000), and GAPDH (Cat No. 5174) were purchased from CST, transferrin (Cat No. ab82411), SLC40A1 (Cat No. ab239583) were purchased from Abcam, and the dilution ratio was determined according to the manufacturer’s instructions. All the full and uncropped western blots are uploaded as ‘Supplemental Material’.

### Cell proliferation assay

The cell proliferation assay was performed using a CCK-8 assay kit (Dojindo Laboratories Co. Ltd., Kumamoto, Japan). Briefly, cells (2 × 10^3^ cells/well) were seeded in 96-well plates with 100 μL per well of RPMI-1640 culture medium, supplemented with 10% FBS and cultured at 37 °C in a 5% CO_2_ atmosphere. Each sample has six replicates. The medium was replaced with 100 μL fresh culture medium, and 10 μL CCK-8 solution was added to each well for different periods (6, 24, 48, 72, and 96 h). After 2 h of incubation, absorbance was measured spectrophotometrically at 450 nm using a Quant ELISA Reader (BioTek Instruments, USA). All experiments were performed in quintuplicate and repeated once.

### Colony formation assay

For the colony formation assay, cells (500 cells/well) were seeded into 6-well plates and maintained in RPMI-1640 medium containing 10% FBS for 2 weeks, replacing the medium every 4 days. After fixation in 4% paraformaldehyde for 10 min, cells were stained with 1% crystal violet. Colonies with diameters greater than 100 μm were counted. Each sample was assessed in triplicates.

### Generation of CCA spheroids

To generate spheroids, cells suspended in complete RPMI-1640 medium were seeded at 2000 cells/well in a 96-well round-bottomed ultra-low attachment surface (Corning). The plates were incubated for 21 days and photographed.

### 5-Ethynyl-2’-deoxyuridine (EdU) Assay

The EdU reagent was purchased from RiboBio (Guangzhou, China). The transfected cells were cultured in confocal dishes, washed, and fixed for 24 h. Next, cells were treated with 0.2% Triton X-100 (Boster, Wuhan, China) for 10 min, incubated with the EdU dye agent for 25 min, and stained with DAPI for 10 min. Finally, images were captured using a fluorescence microscope.

### RNA pull-down assay

Control and biotin-labeled linc00976 probes were synthesized by GeneChem (Guangzhou, China). C-1 magnetic beads (Life Technologies Corporation, USA) were used to incubate the probes for 2 h at room temperature. The transfected PC cells were lysed on ice and incubated overnight with the appeal-treated probes at 4 °C. Finally, the precipitate was collected and purified using an RNeasy Mini Kit (Qiagen). The linc00976 expression abundance in the RNA complex was examined by RT-qPCR.

### RNA-binding protein

The RIP assay was performed using the Magna RIP RNA-Binding Protein Immunoprecipitation Kit (Millipore, USA), in accordance with the manufacturer’s instructions. In brief, transfected cells were washed twice in ice-cold PBS and lysed in an equal volume of RIP lysis buffer supplemented with a protease inhibitor cocktail and RNase inhibitors. Subsequently, the cell lysates (200 μL) were incubated with immunoprecipitation buffer containing magnetic beads conjugated to anti-Ago2 antibody (Millipore, USA) or negative anti-IgG antibody (Millipore, USA) at 4 °C overnight. After washing beads with proteinase K buffer, immunoprecipitated RNAs were extracted and purified to further detect the abundance of the target RNAs by RT-qPCR.

### Luciferase reporter assays

HuCCT1 and RBE cells were cultured in 24-well plates. Then, miR-3202 and corresponding NC mimics were co-transfected into different groups of cells. After 48 h, the cells were lysed, and firefly and Renilla luciferase activities were detected in the lysate. Firefly luciferase activity was used as a control to quantify relative activity.

### Animal experiments

All conditions and procedures for animal experiments were approved by the Animal Care Committee of Guizhou Medical University. BALB/c nude mice (8 weeks old) were obtained from HFKBio and randomly assigned to each group (*n* = 5 per group).

For the proliferation assays, HuCCT1 cells with linc00976 knockdown, linc00976 overexpression, and negative control were subcutaneously injected into BALB/c nude mice. The mice were weighed every week and euthanized 5 weeks after injection. Finally, tumors were dissected and weighed.

For lung metastasis assays, the abovementioned cells were injected into nude mice via the tail vein (2 × 10^6^ HuCCT1 cells per mouse, *n* = 5 per group). Five weeks after injection, mice were euthanized, and lung tissues were harvested and photographed. IHC was used to detect metastatic loci in the lung.

### IHC and ISH

IHC and in situ hybridization (ISH) were performed as previously described [10]. Then sections were incubated against KI67(Cat No. 9449), and anti-PCNA (Cat No. 2586) were purchased from CST. The results were evaluated blindly by two independent pathologists.

### ChIP-PCR assay

PC cells were crosslinked with 1% formaldehyde for 20 min at room temperature. After breaking DNA into 200–500 bp using ultrasound, Pierce Magnetic ChIP Kit (Thermo Fisher Scientific, USA), combined with JUND antibody (1:100) or IgG antibody (1:100), was used to enrich the DNA fragment. The purified immunoprecipitated DNA fragments were analyzed using RT-qPCR.

### Measurement of cellular ferroptosis levels

The GSH concentrations were analyzed using a glutathione assay kit (Solarbio, Beijing, China) according to the manufacturer’s instructions. The MDA concentrations were analyzed using a lipid peroxidation assay kit (Solarbio, Beijing, China) according to the manufacturer’s instructions. Iron concentrations were analyzed using an iron assay kit (Solarbio, Beijing, China) according to the manufacturer’s instructions. Relative GPX activity was analyzed using a GPXs Assay Kit (Solarbio, Beijing, China) according to the manufacturer’s instructions.

### Statistical analysis

All statistical analyses were performed using the SPSS version 17 (IBM, Chicago, USA). Two-sided *P* values were calculated, and a threshold of *P* < 0.05 was considered statistically significant. The results are expressed as the mean ± SD. Statistical significance was assigned at *P < 0.05 or **P < 0.01.

## Supplementary information

Fig. S1

Fig. S2

Fig. S3

Fig. S4

Supplemental legends

Supplemental Material

Reproducibility checklist

Table S1

Table S2

Certificate_of_editing

## Data Availability

The datasets used and/or analyzed during the current study are available from the corresponding author upon reasonable request.
