# Supplementary figures and images for: JUND/linc00976 promotes cholangiocarcinoma progression and metastasis, inhibits ferroptosis by regulating the miR-3202/GPX4 axis
Source: Cell Death Dis. 2022 Nov 18;13(11):967. doi: 10.1038/s41419-022-05412-5 (PMC9674662; doi:10.1038/s41419-022-05412-5)

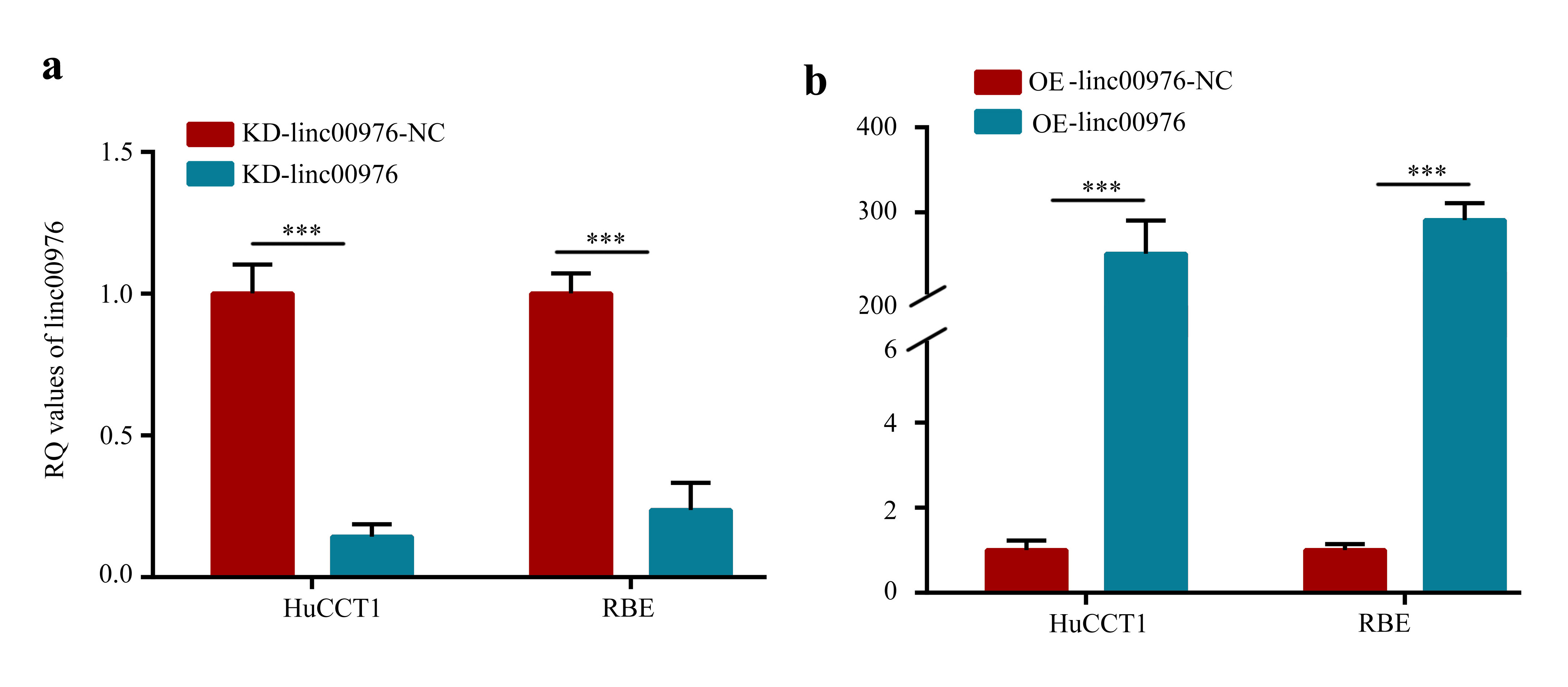

Supplement: Supplementary file 1 — Fig. S1 [file 41419_2022_5412_MOESM1_ESM.jpg]

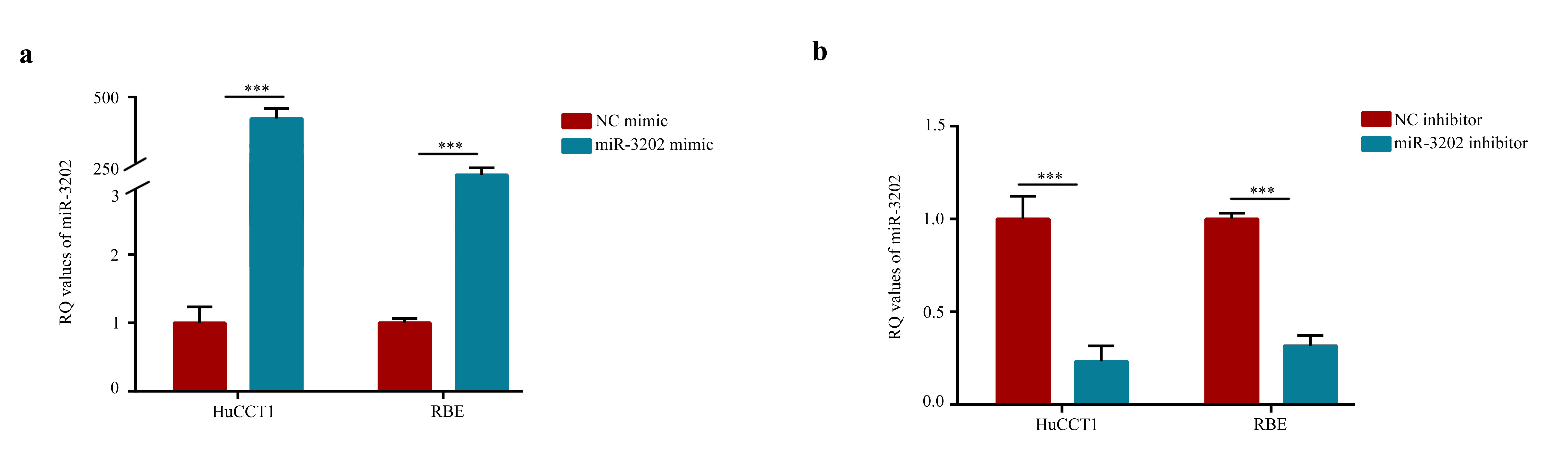

Supplement: Supplementary file 2 — Fig. S2 [file 41419_2022_5412_MOESM2_ESM.jpg]

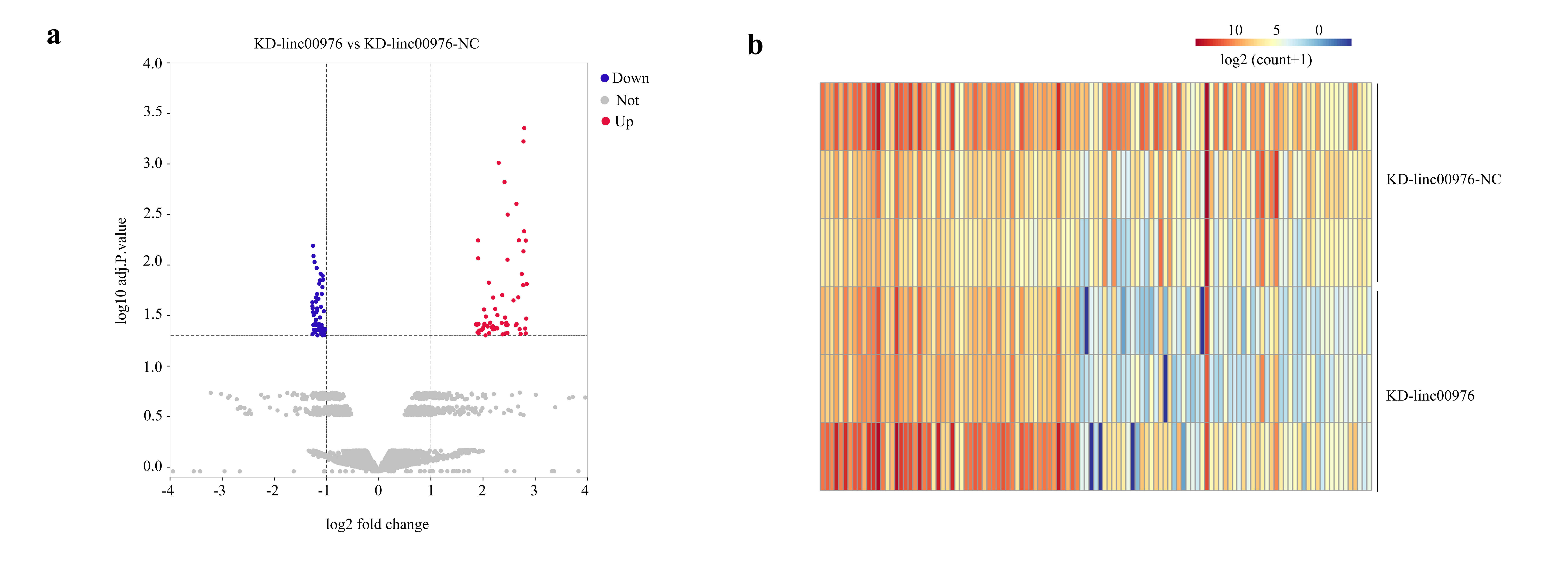

Supplement: Supplementary file 3 — Fig. S3 [file 41419_2022_5412_MOESM3_ESM.jpg]

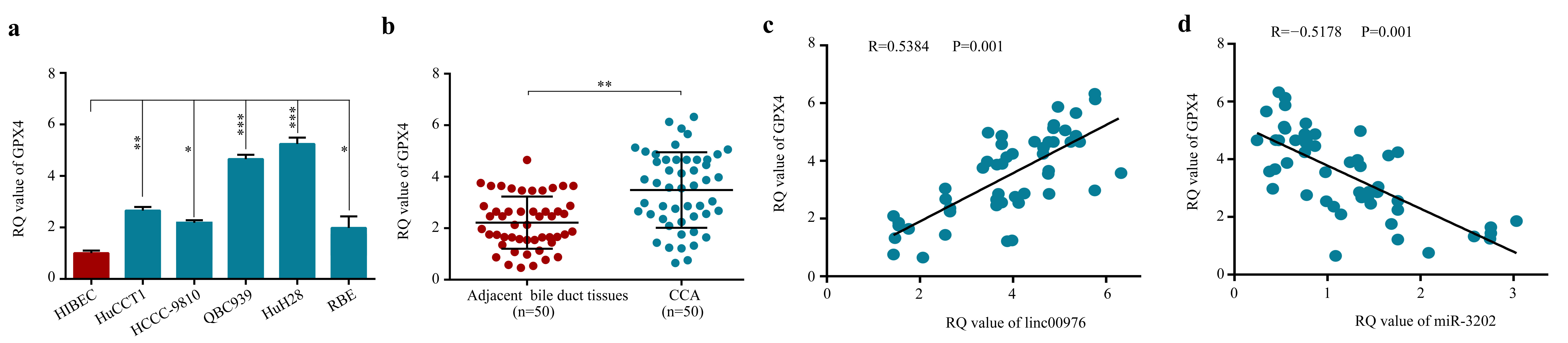

Supplement: Supplementary file 4 — Fig. S4 [file 41419_2022_5412_MOESM4_ESM.jpg]
