# Supplementary material for: JUND/linc00976 promotes cholangiocarcinoma progression and metastasis, inhibits ferroptosis by regulating the miR-3202/GPX4 axis
Source: Cell Death Dis. 2022 Nov 18;13(11):967. doi: 10.1038/s41419-022-05412-5 (PMC9674662; doi:10.1038/s41419-022-05412-5)
Supplement: Supplementary file 5 — Supplemental legends [file 41419_2022_5412_MOESM5_ESM.docx]

**Additional file 1: Table S1. Linc00976 potential transcription factor and GPX4 co-expression relationship in TCGA database.**

**Table S2.** **Primer sequences for RT-qPCR used in this study.**

**Additional file 2: Figure S1.** **linc00976 promotes the proliferation and metastasis of cholangiocarcinoma (CCA) cells**

a. Knockdown efficiency of linc00976 was verified by RT-qPCR.

b. Overexpression efficiency of linc00976 was verified by RT-qPCR.

**Figure S2.** **linc00976 acts as a sponge for miR-3202**

a-b. Transfection efficiencies of miR-3202 mimics and inhibitors in cholangiocarcinoma (CCA) cells were verified by RT-qPCR.

**Figure S3. Screening of differentially expressed genes of negative control and knockdown linc00976**

a. Volcano plot exhibiting changes in differentially expressed genes (DEGs) between negative control and knockdown linc00976 in HuCCT1 cells.

b. Heatmap plots exhibiting DEGs in negative control and knockdown linc00976 in HuCCT1 cells.

**Figure S4. Gain-of-function assays confirm the involvement of the linc00976/miR-3202/GPX4 axis in cholangiocarcinoma (CCA) progression**

a. The expression level of linc00976 was detected by RT-qPCR in 50 paired CCA tissues and adjacent non-cancerous tissues.

b. RT-qPCR analysis of relative expression levels of linc00976 in CCA cell lines and HIBEC cells.

c. Pearson correlation analysis of linc00976 and GPX4 expression in 50 CCA tissues.

d. Pearson correlation analysis of miR-3202 and GPX4 expression in 50 CCA tissues.
