# Supplementary material for: JUND/linc00976 promotes cholangiocarcinoma progression and metastasis, inhibits ferroptosis by regulating the miR-3202/GPX4 axis
Source: Cell Death Dis. 2022 Nov 18;13(11):967. doi: 10.1038/s41419-022-05412-5 (PMC9674662; doi:10.1038/s41419-022-05412-5)

Figure 2j Transferrin

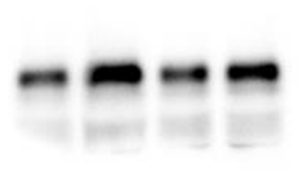

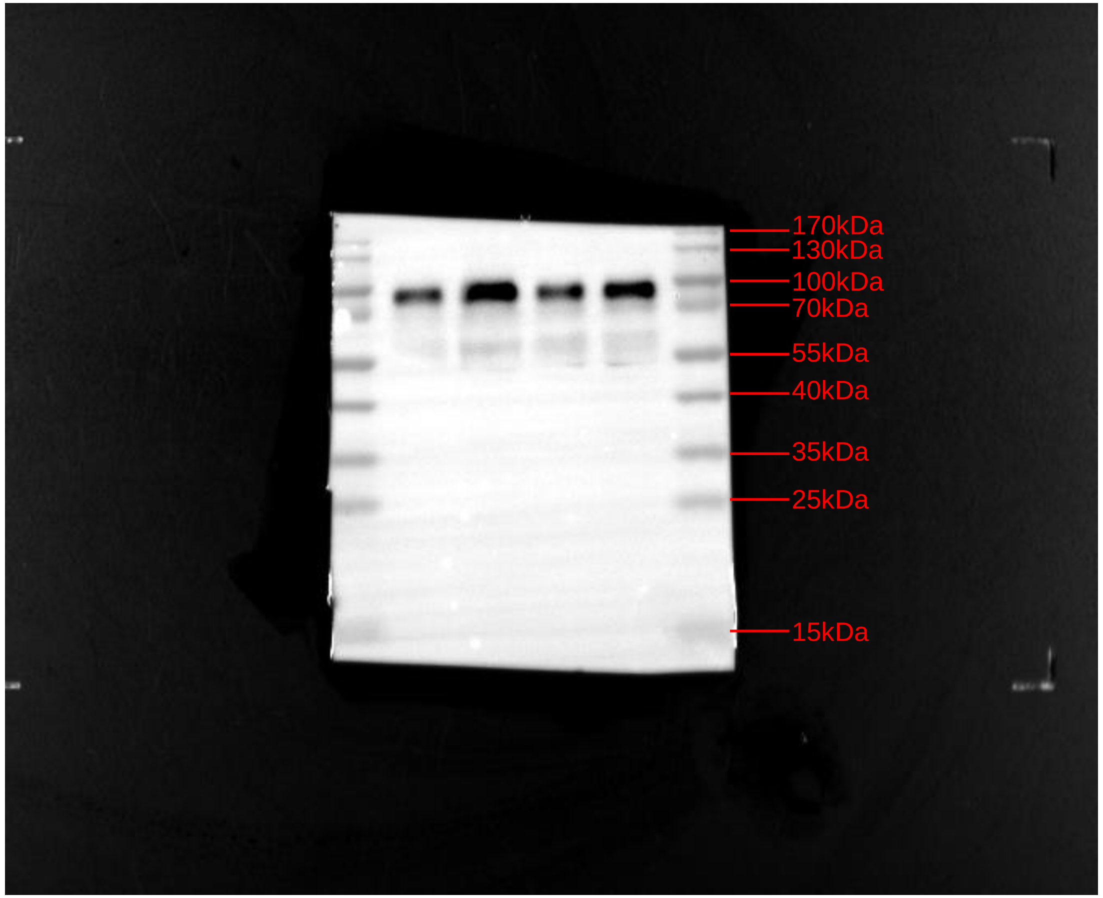

Figure 2j SLC40A1

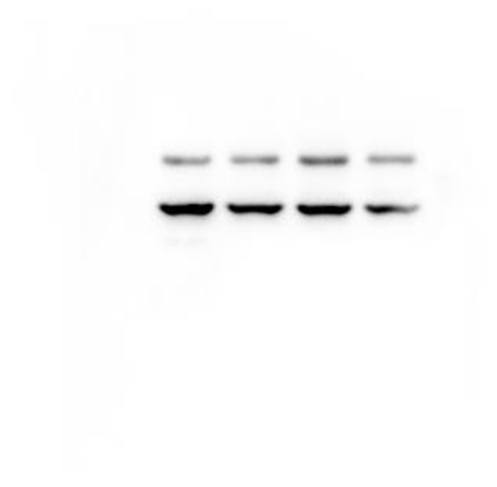

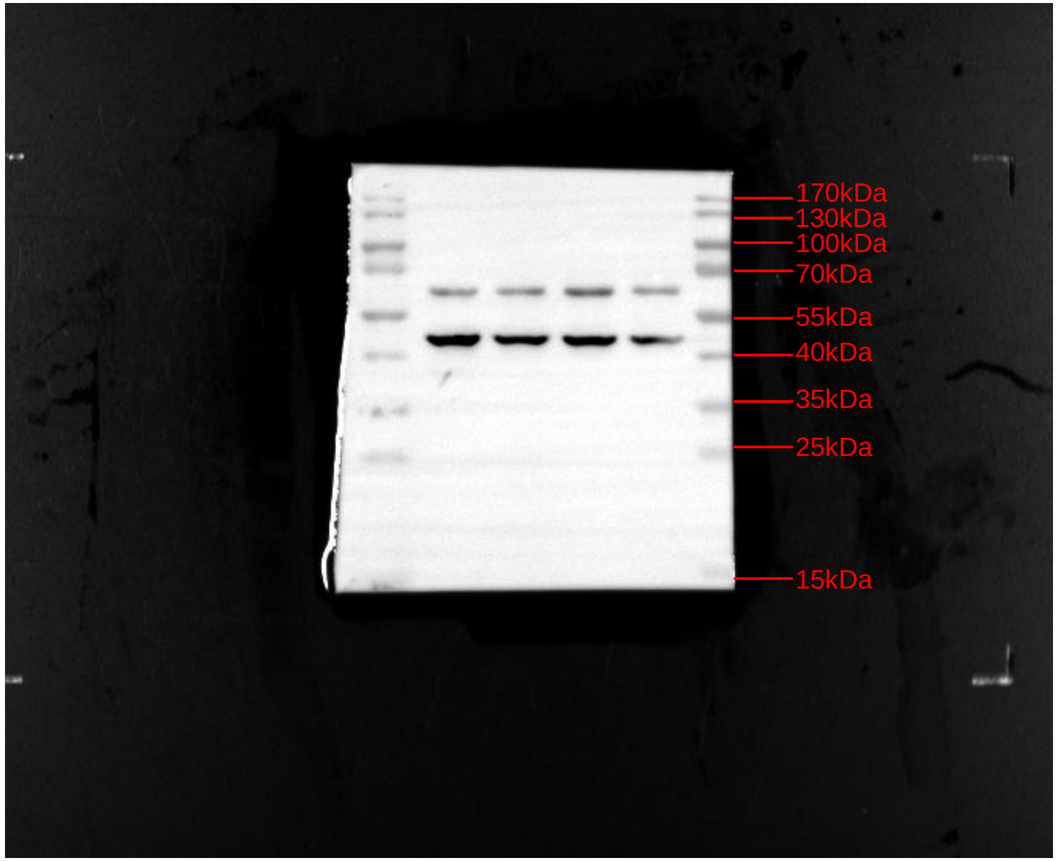

Figure 2j COX2

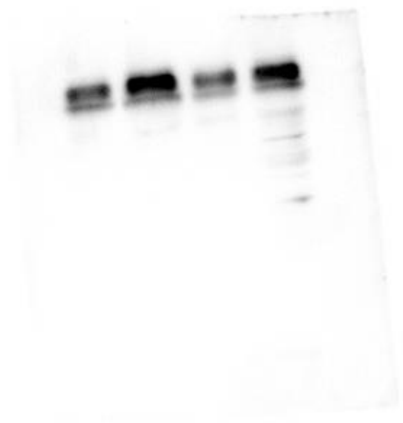

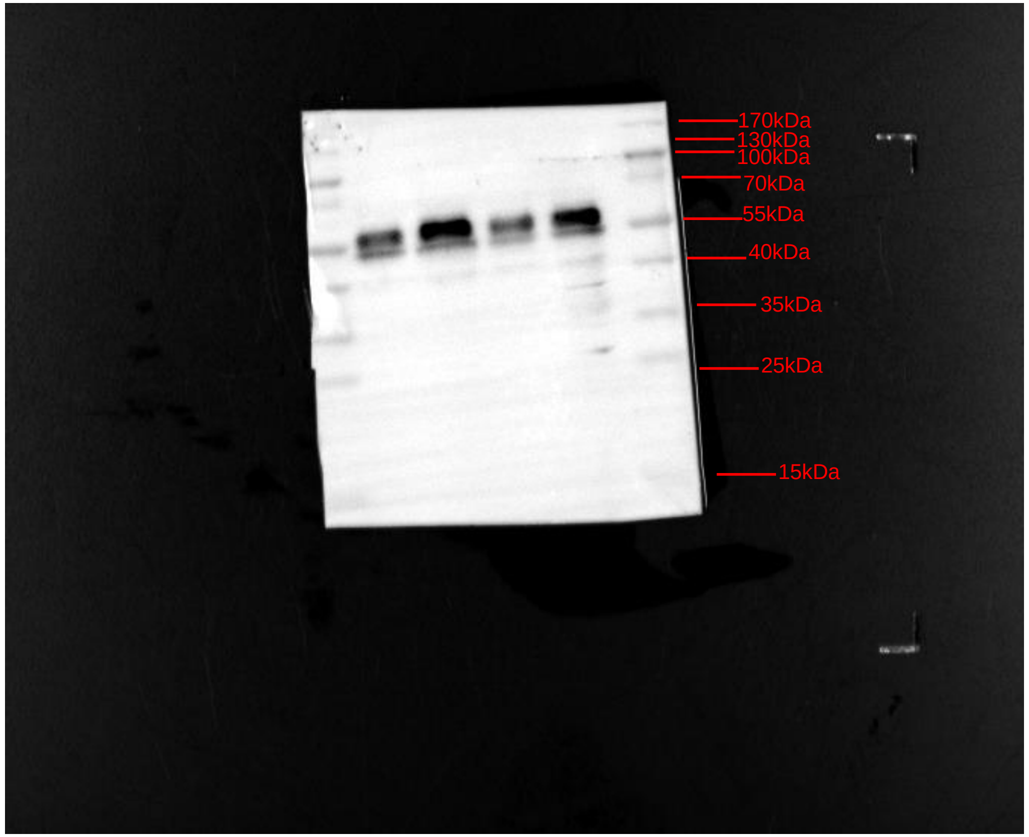

Figure 2j SLC7A11

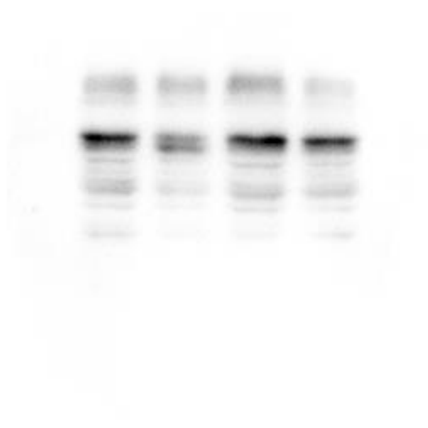

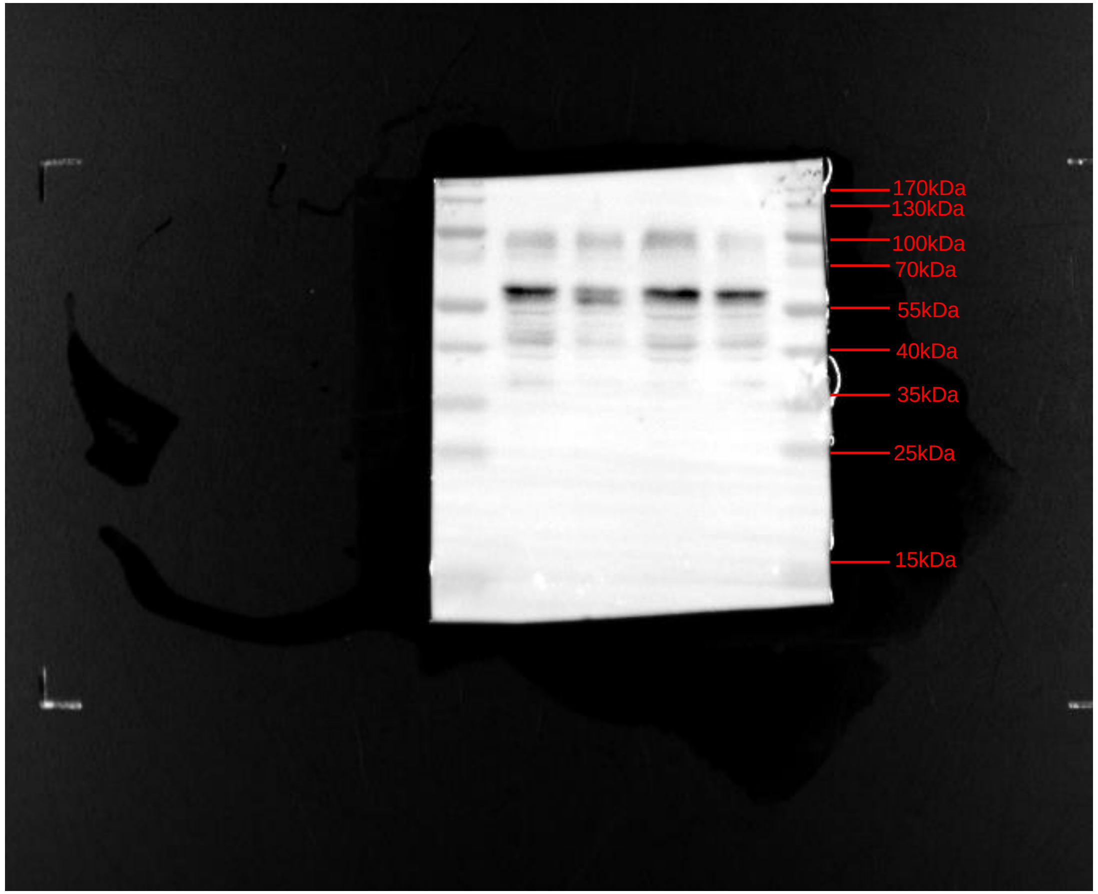

Figure 2j GPX4

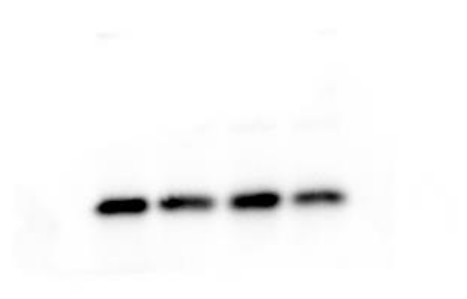

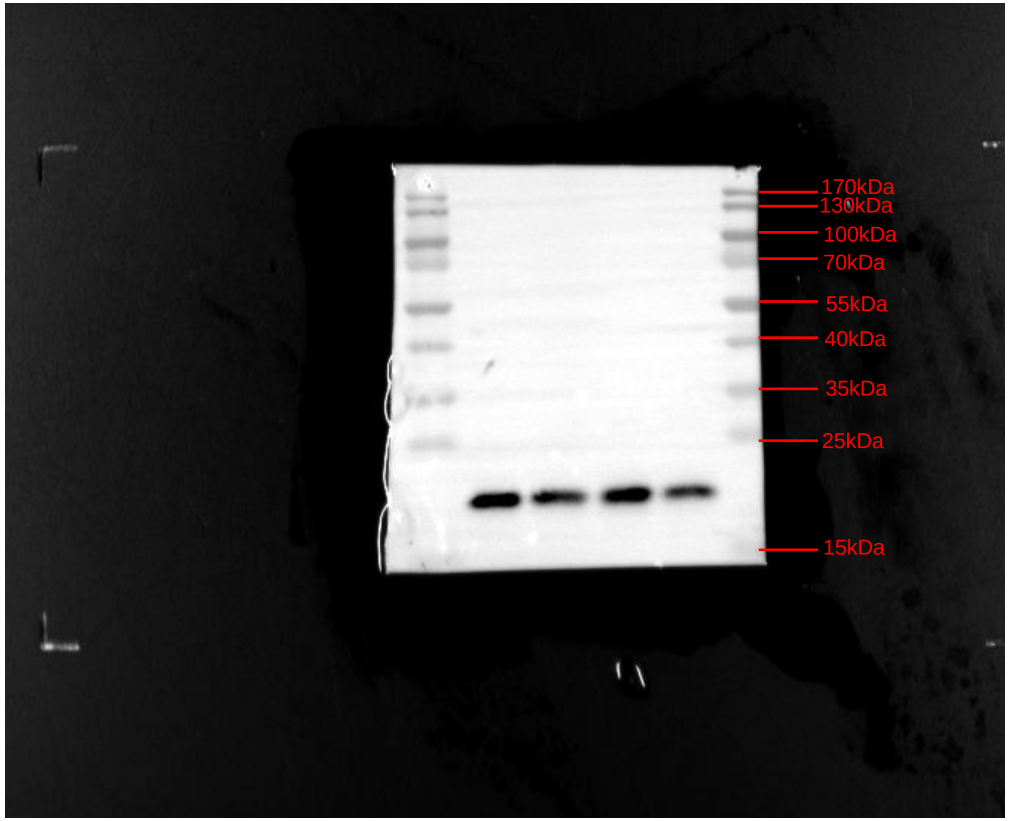

Figure 2j GPADH

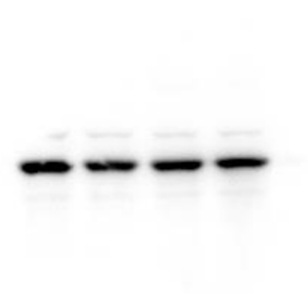

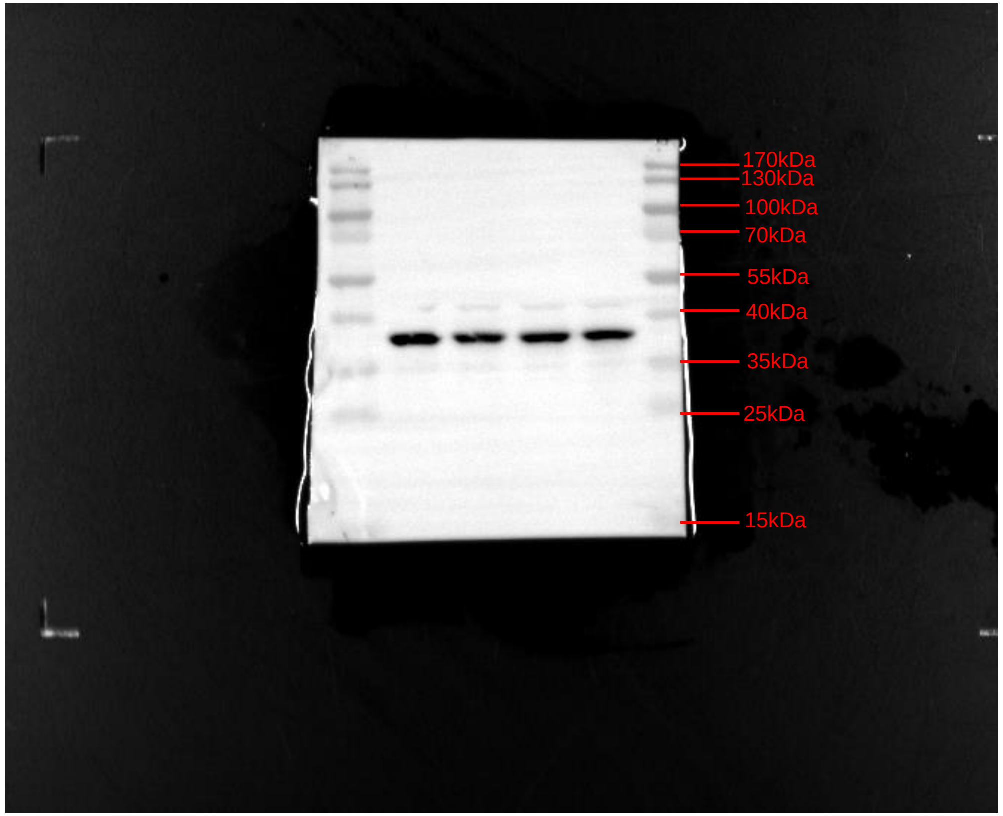

Figure 2i E-cadherin

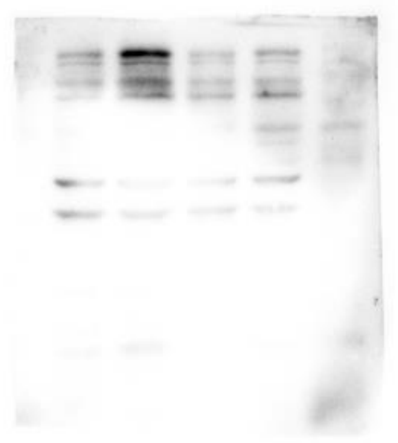

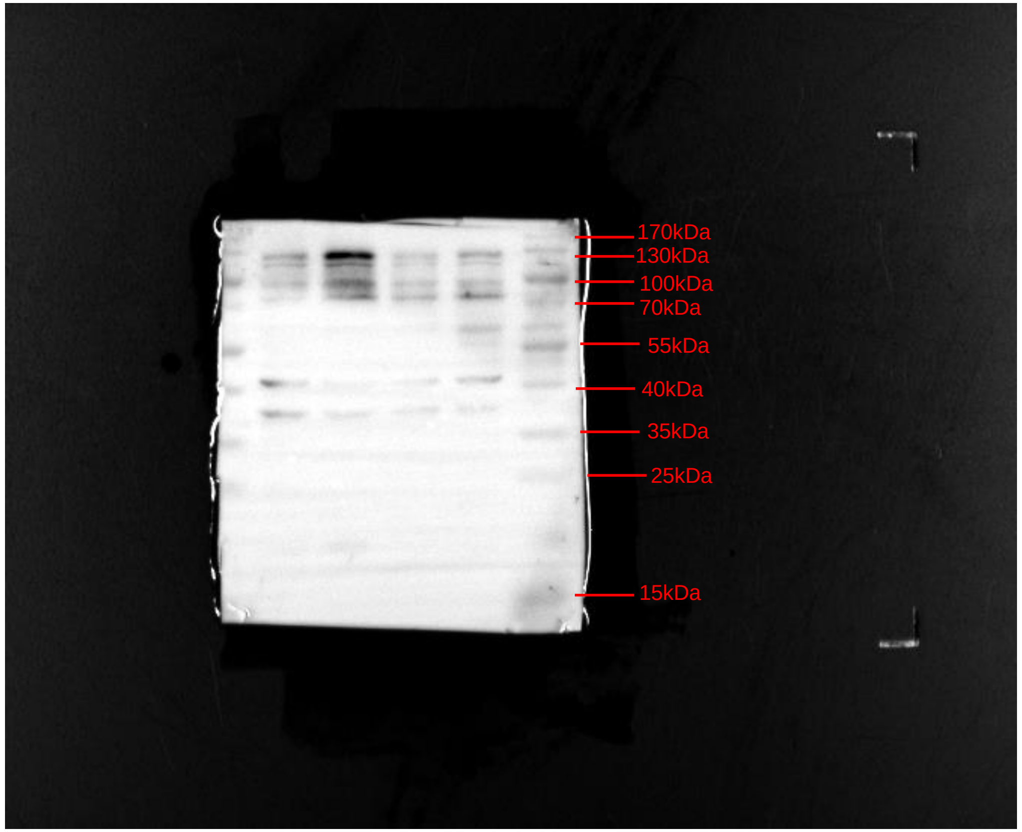

Figure 2i N-cadherin

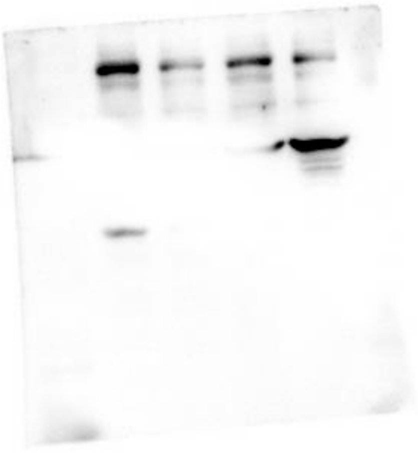

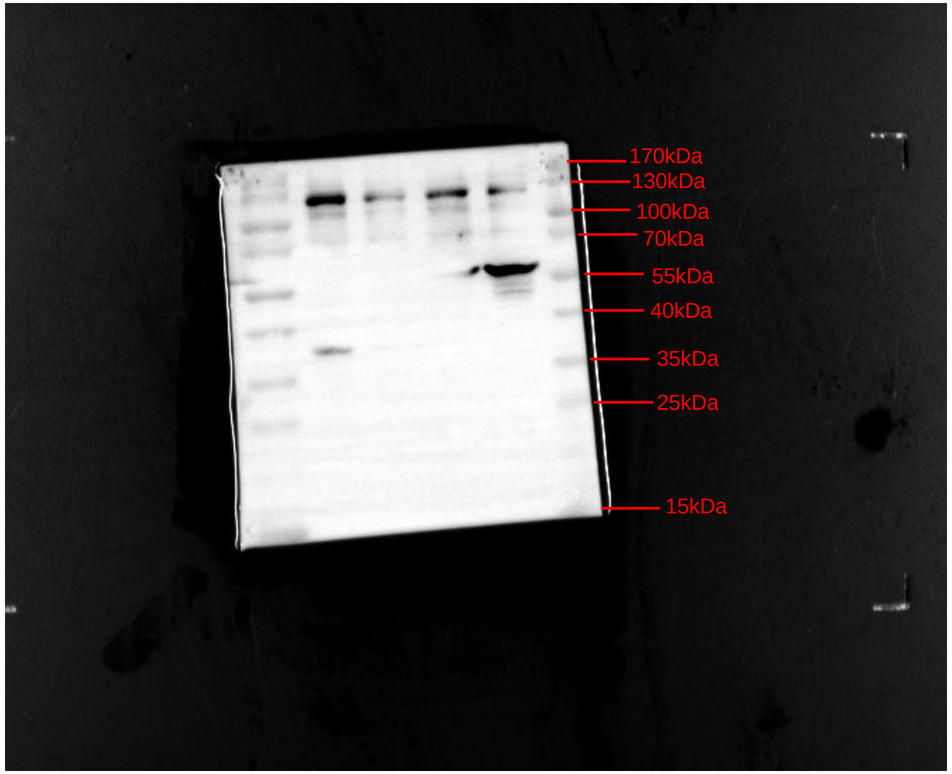

Figure 2i Vimentin

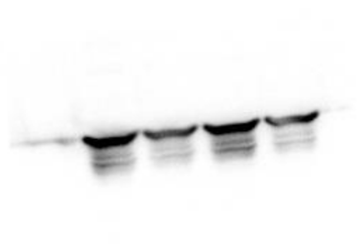

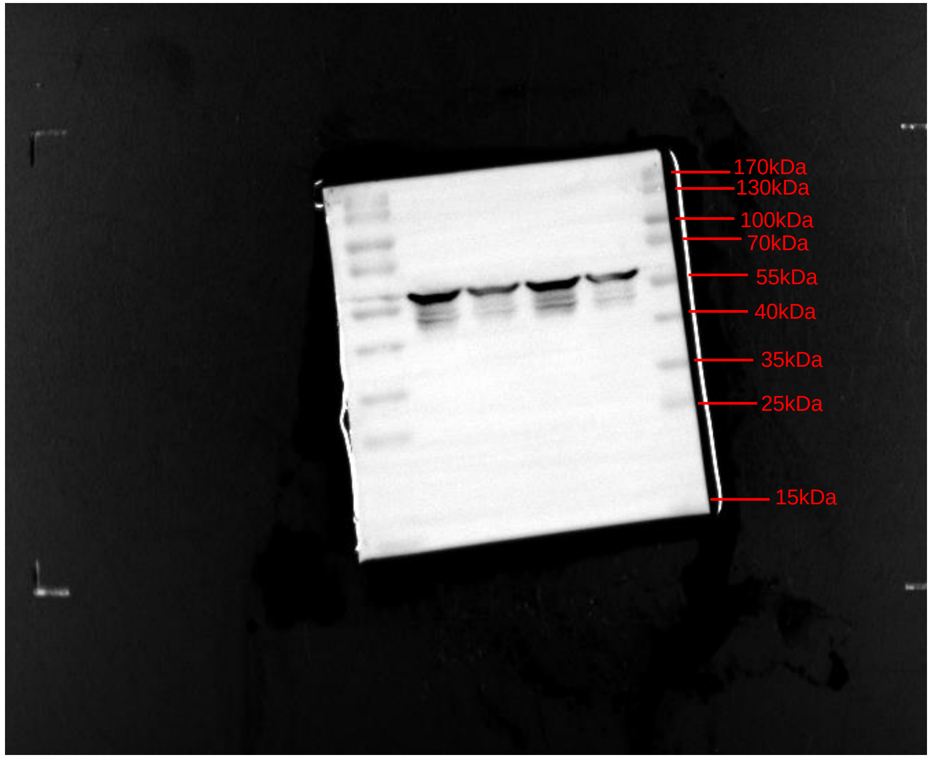

Figure 2i GAPDH

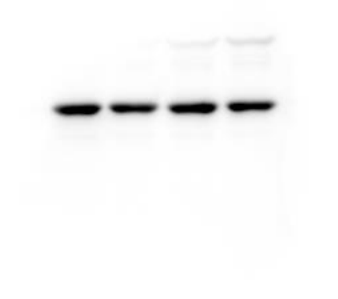

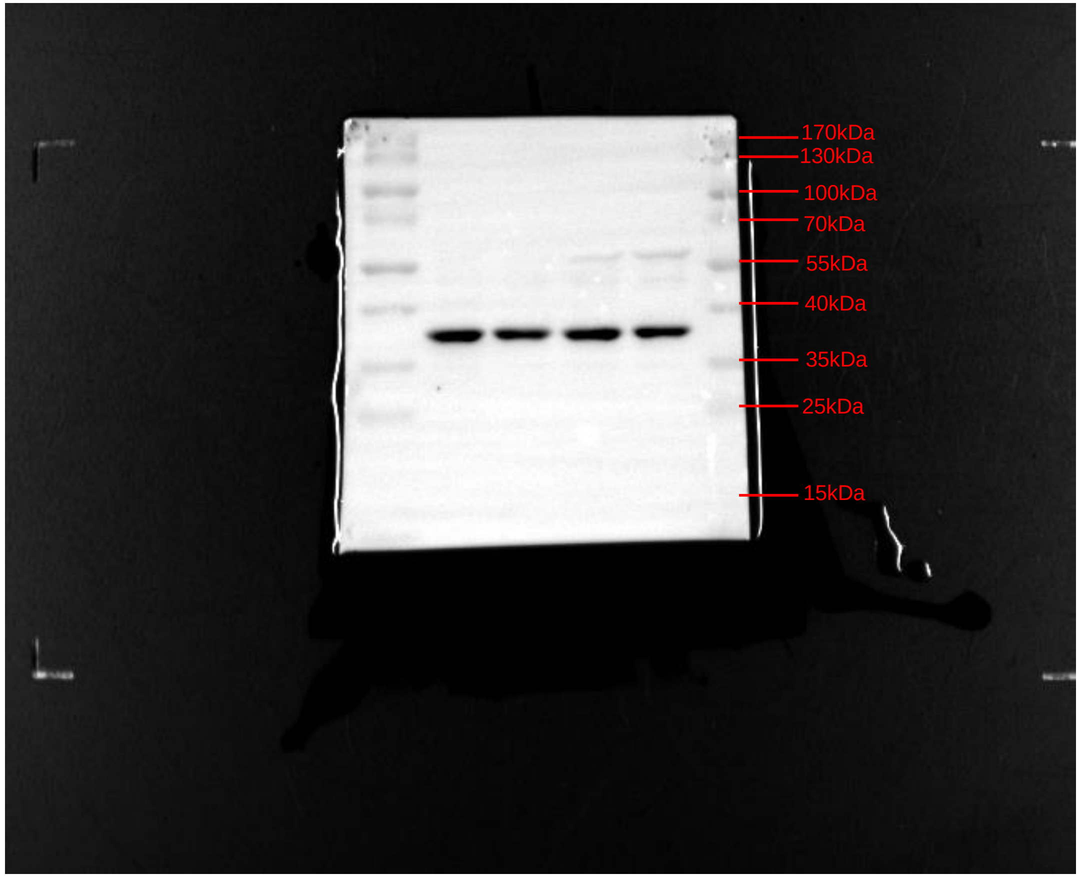

Figure 3j Transferrin

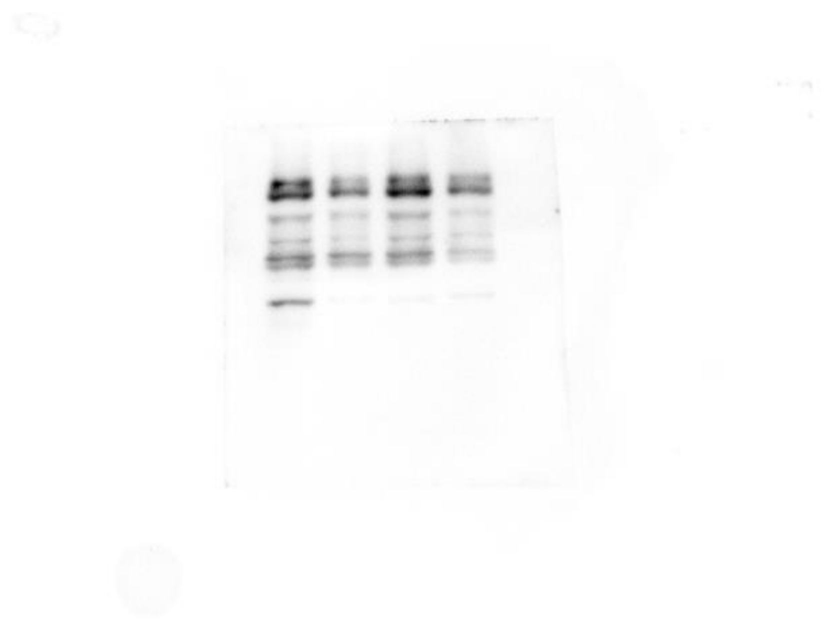

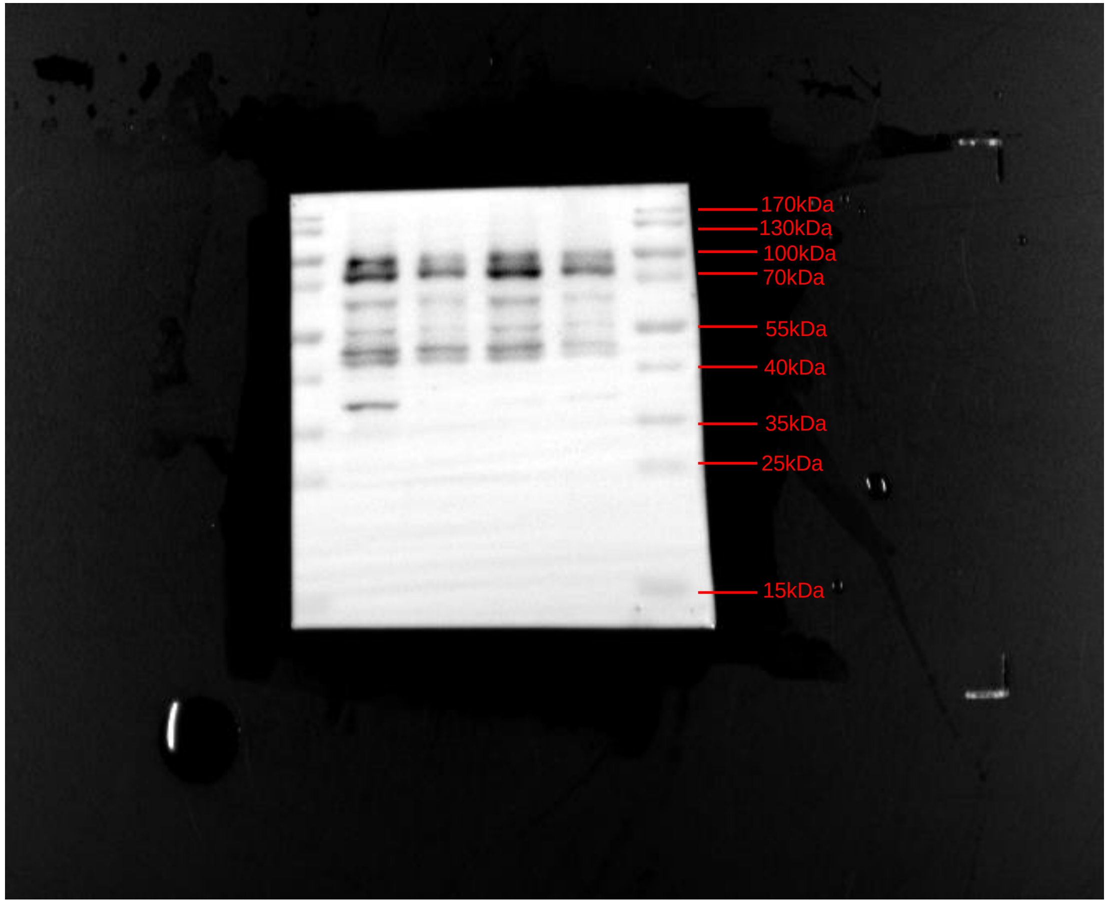

Figure 3j SLC40A1

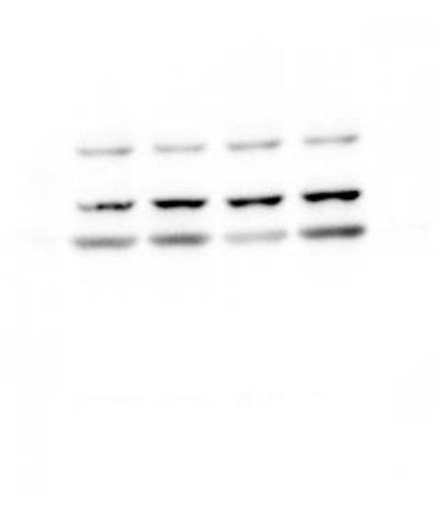

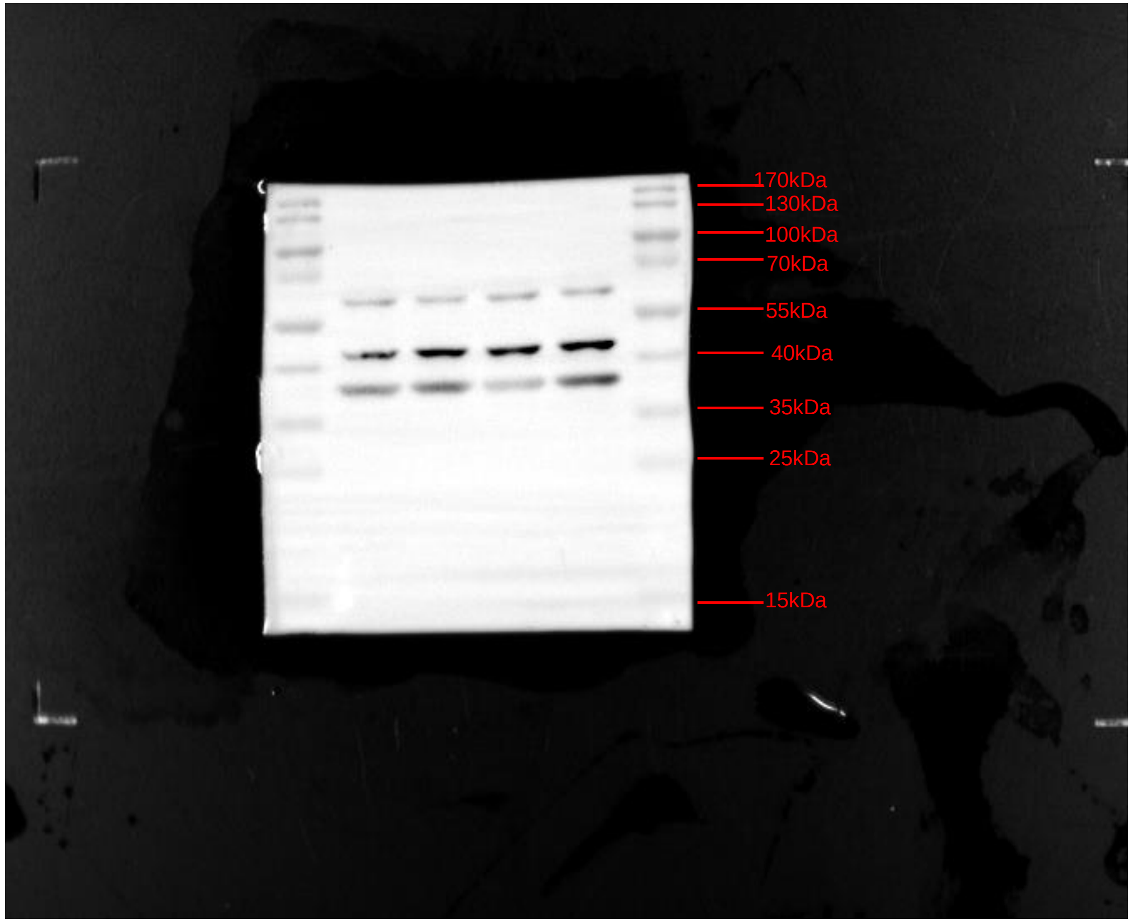

Figure 3j COX2

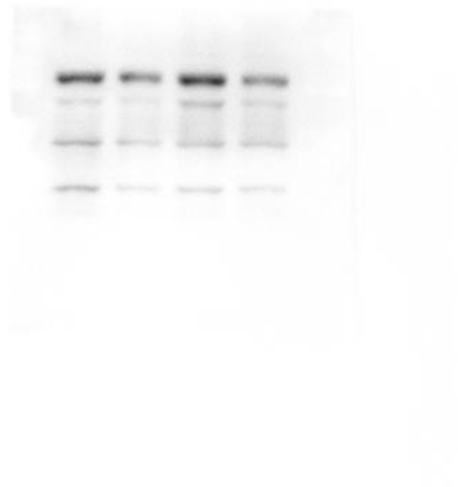

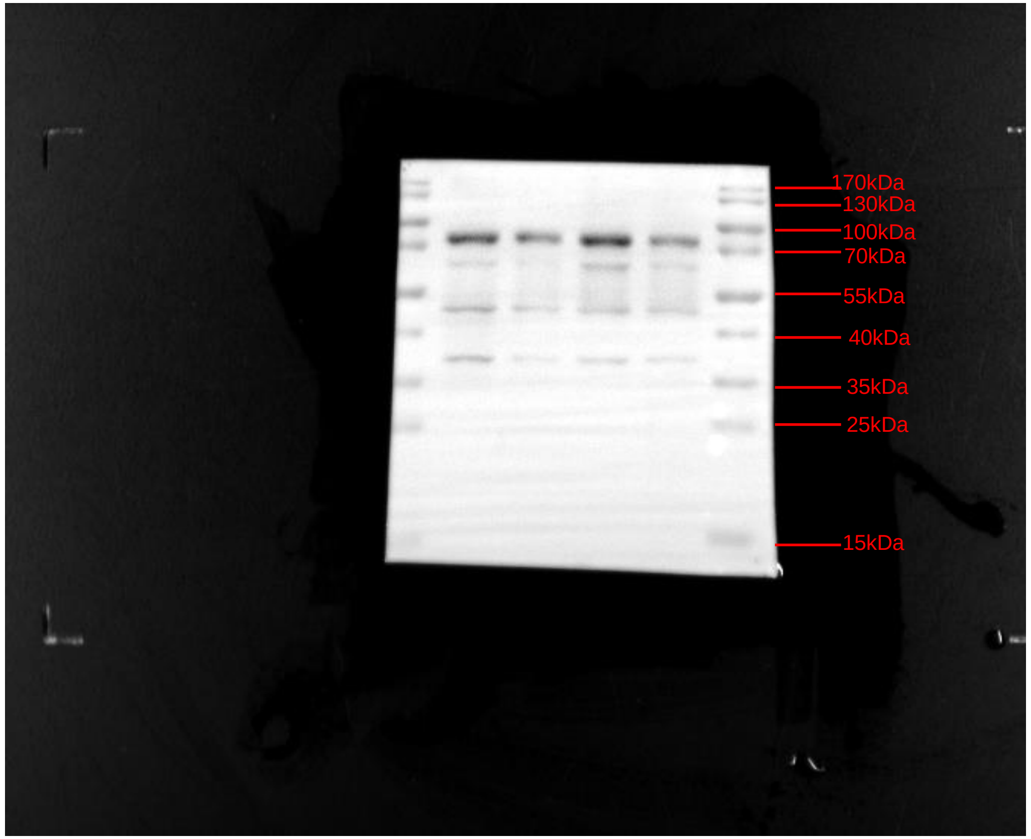

Figure 3j SLC7A11

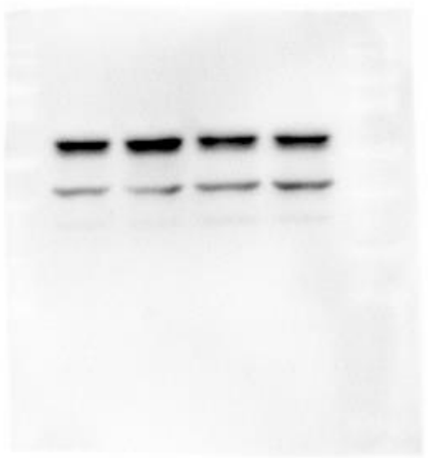

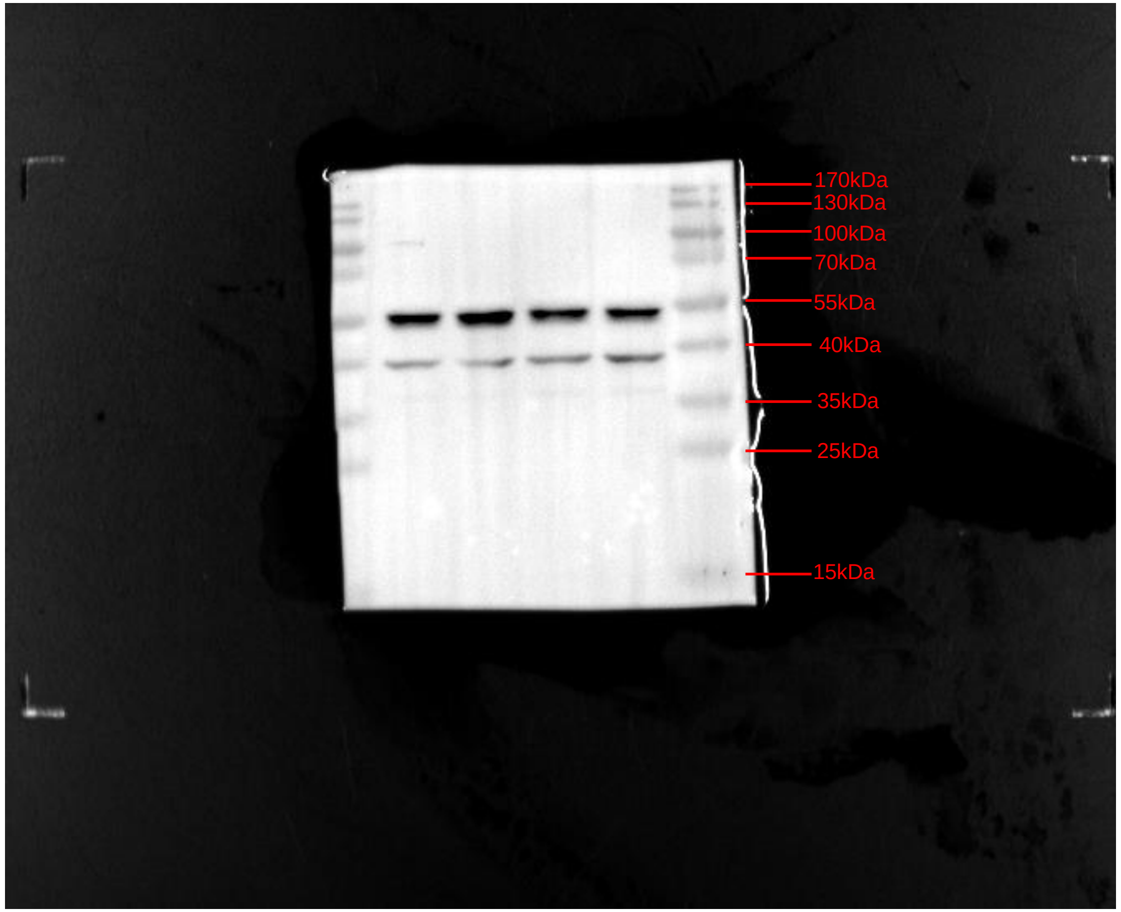

Figure 3j GPX4

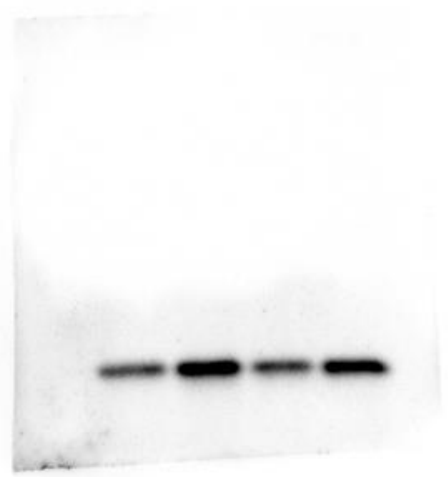

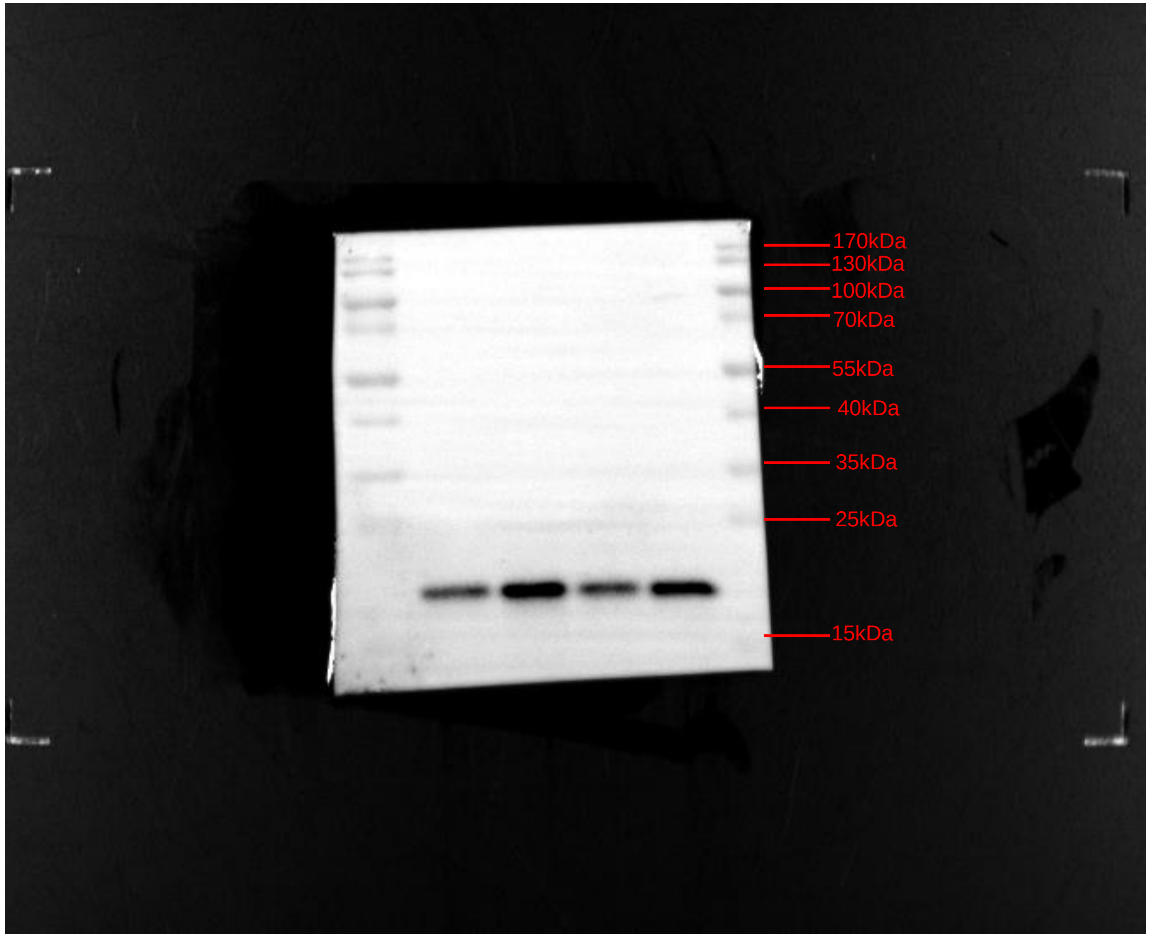

Figure 3j GPADH

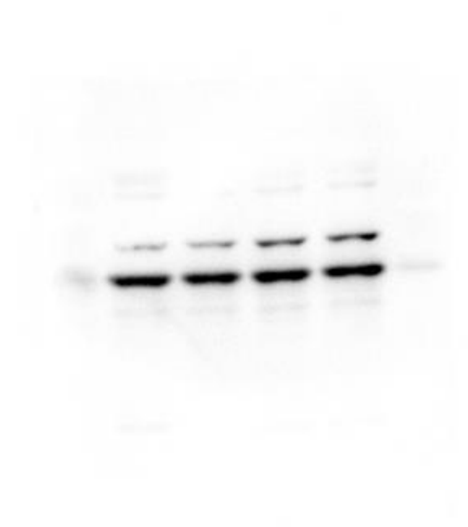

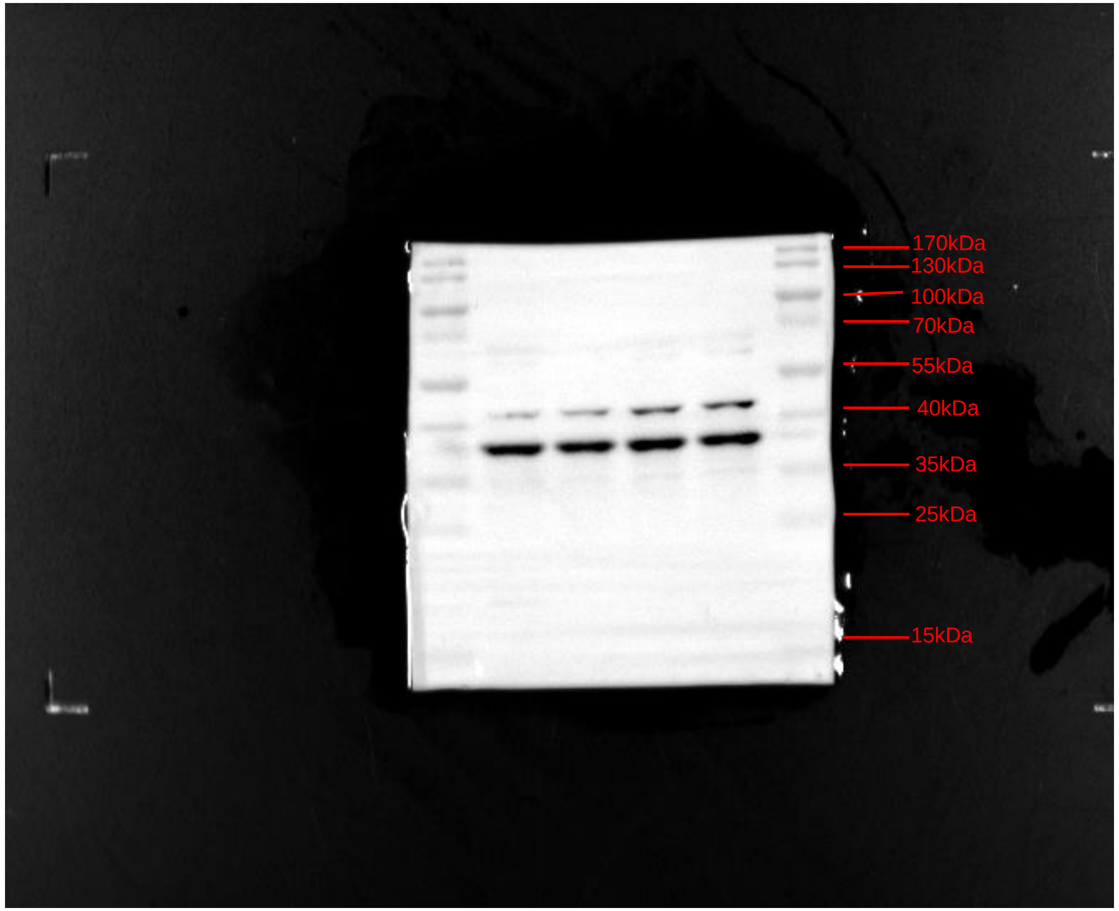

Figure 3i E-cadherin

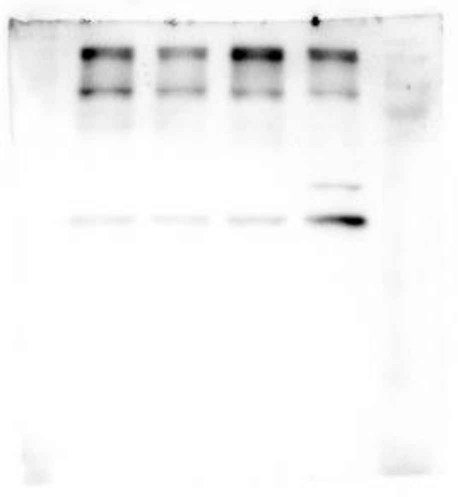

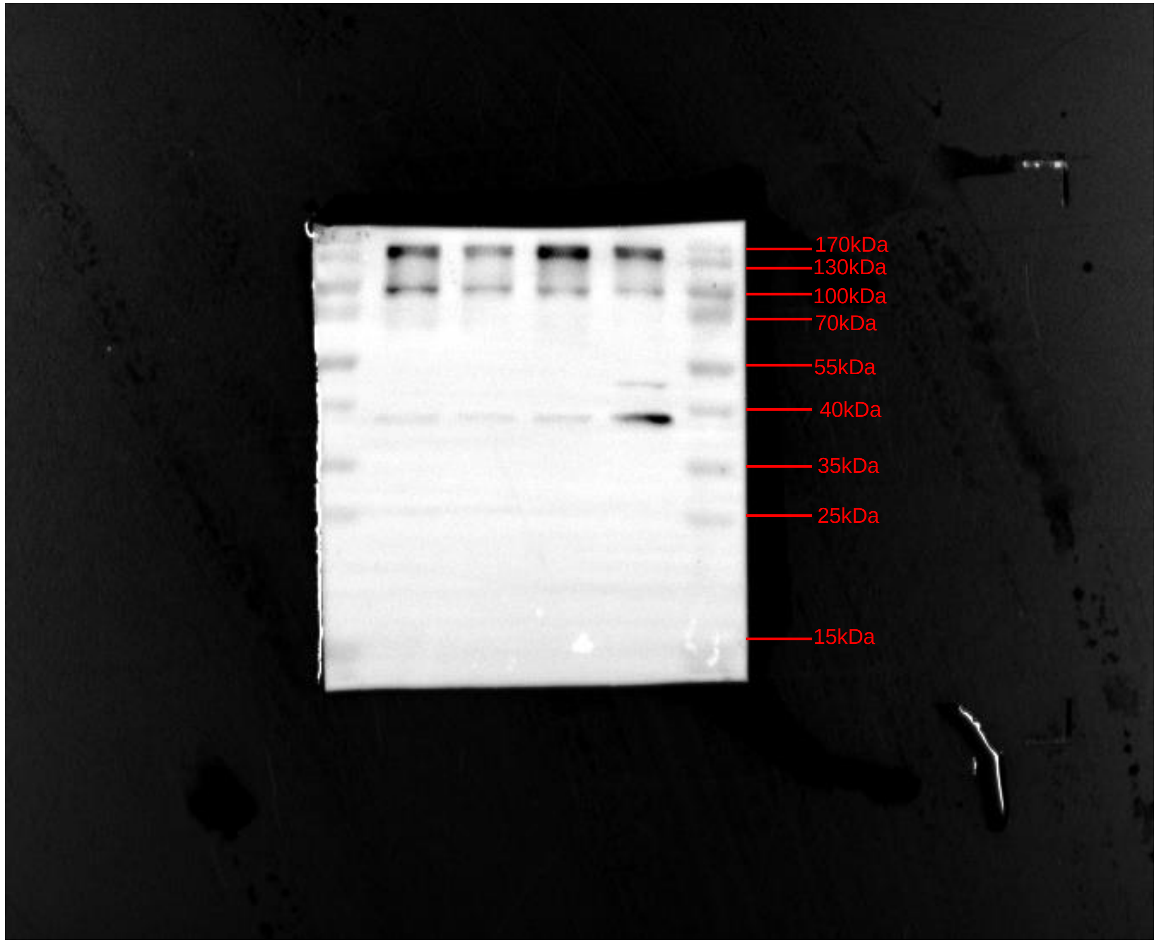

Figure 3i N-cadherin

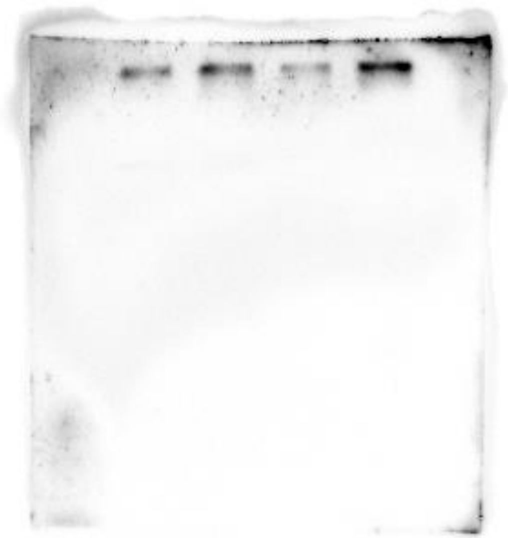

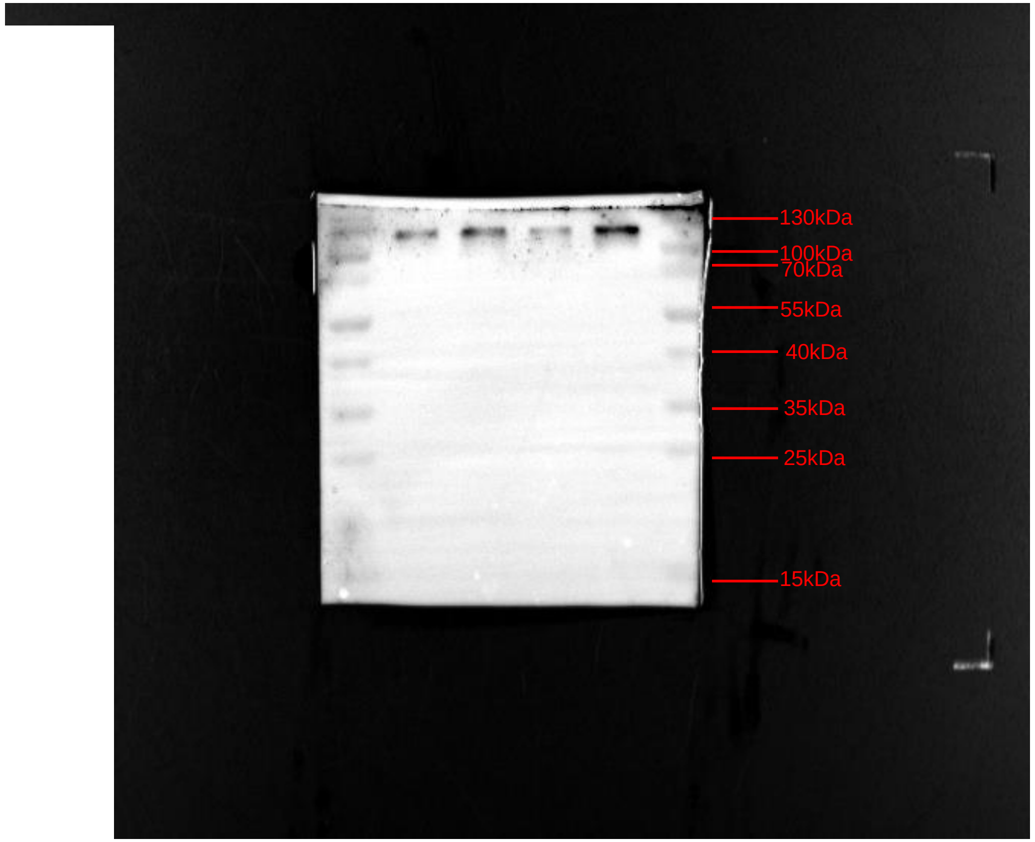

Figure 3i Vimentin

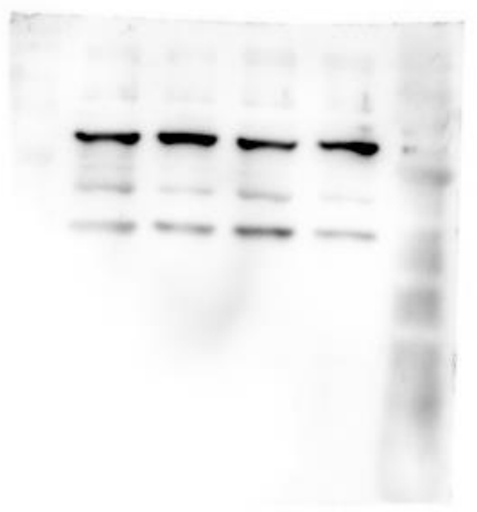

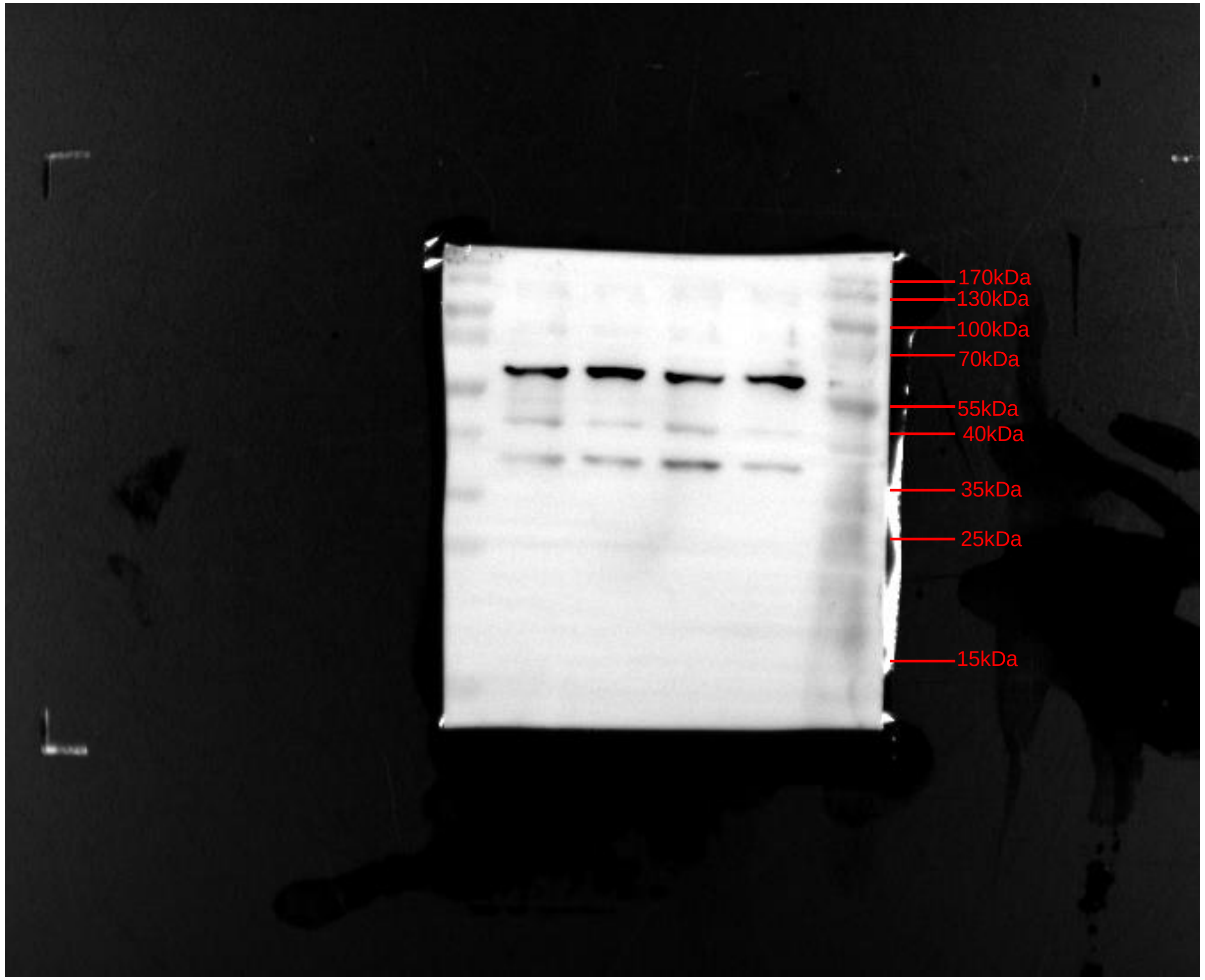

Figure 3i GAPDH

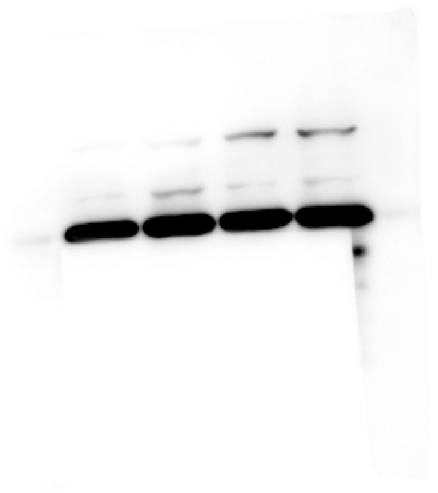

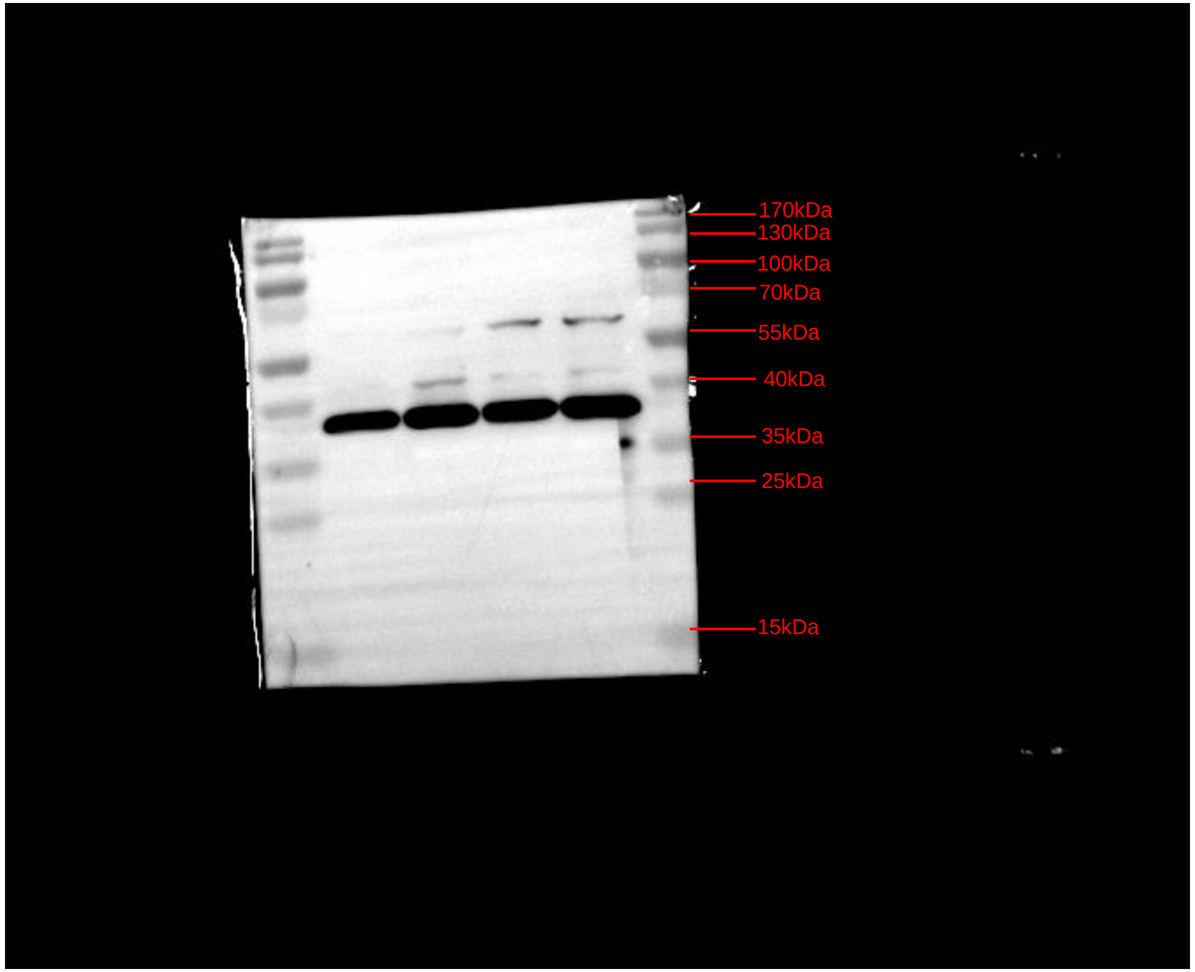

Figure 5h E-cadherin HuCCT1

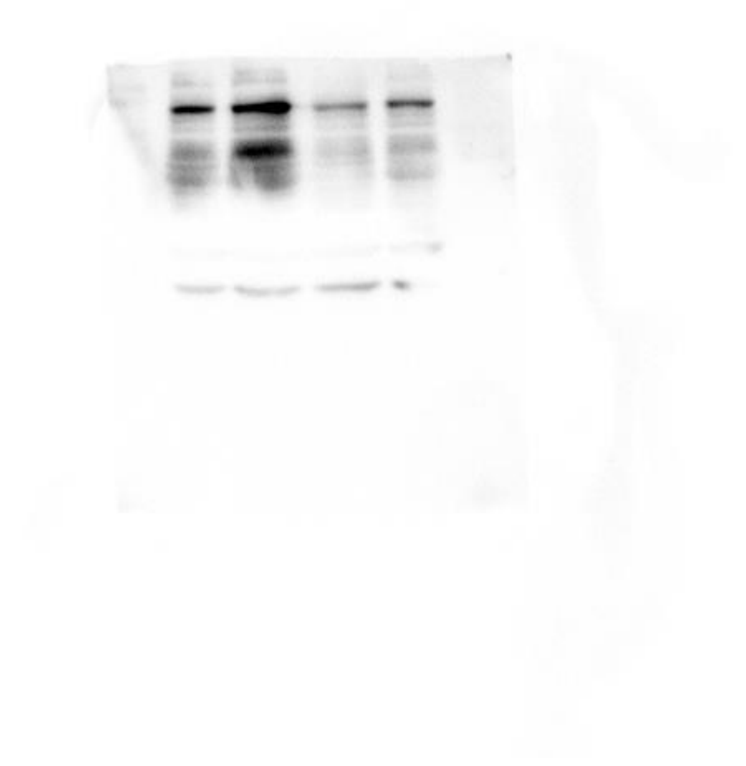

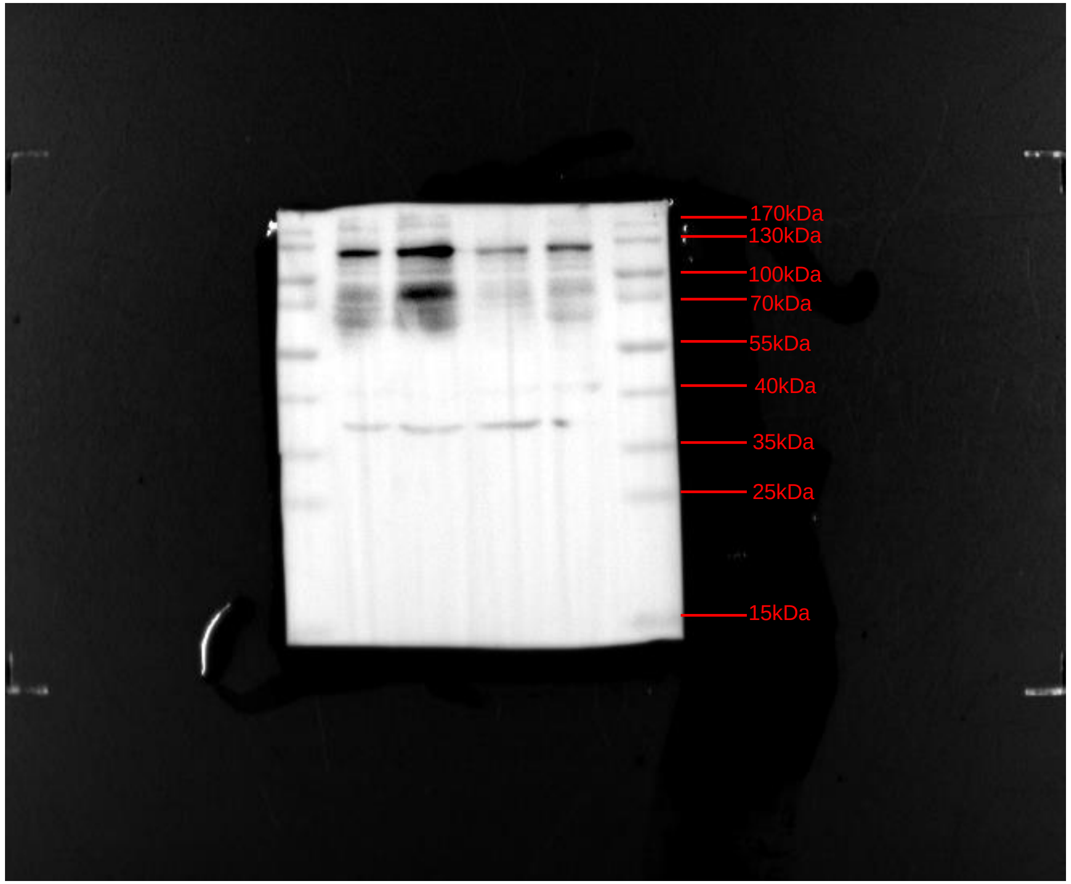

Figure 5h N-cadherin HuCCT1

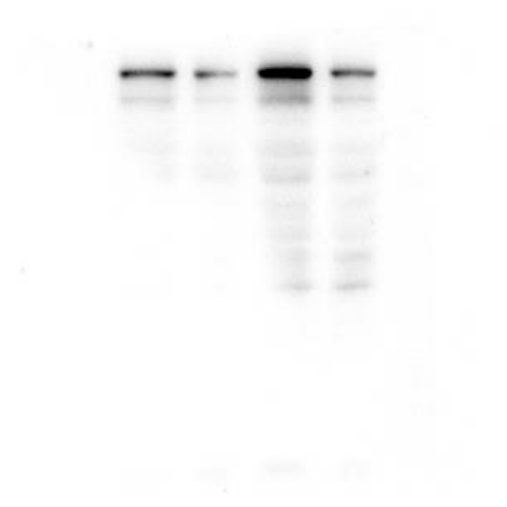

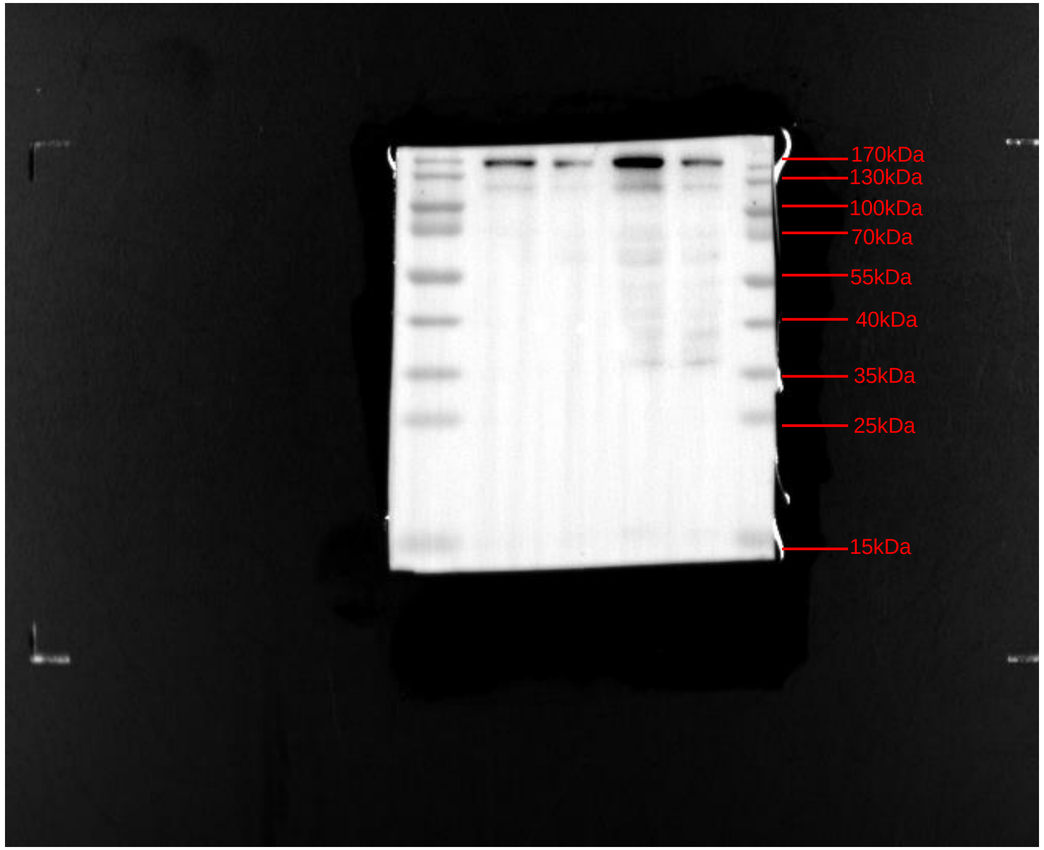

Figure 5h Vimentin HuCCT1

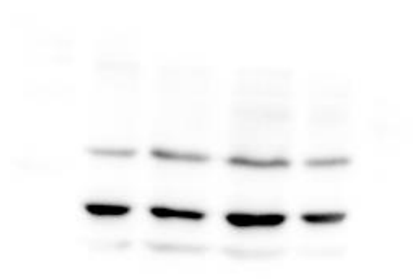

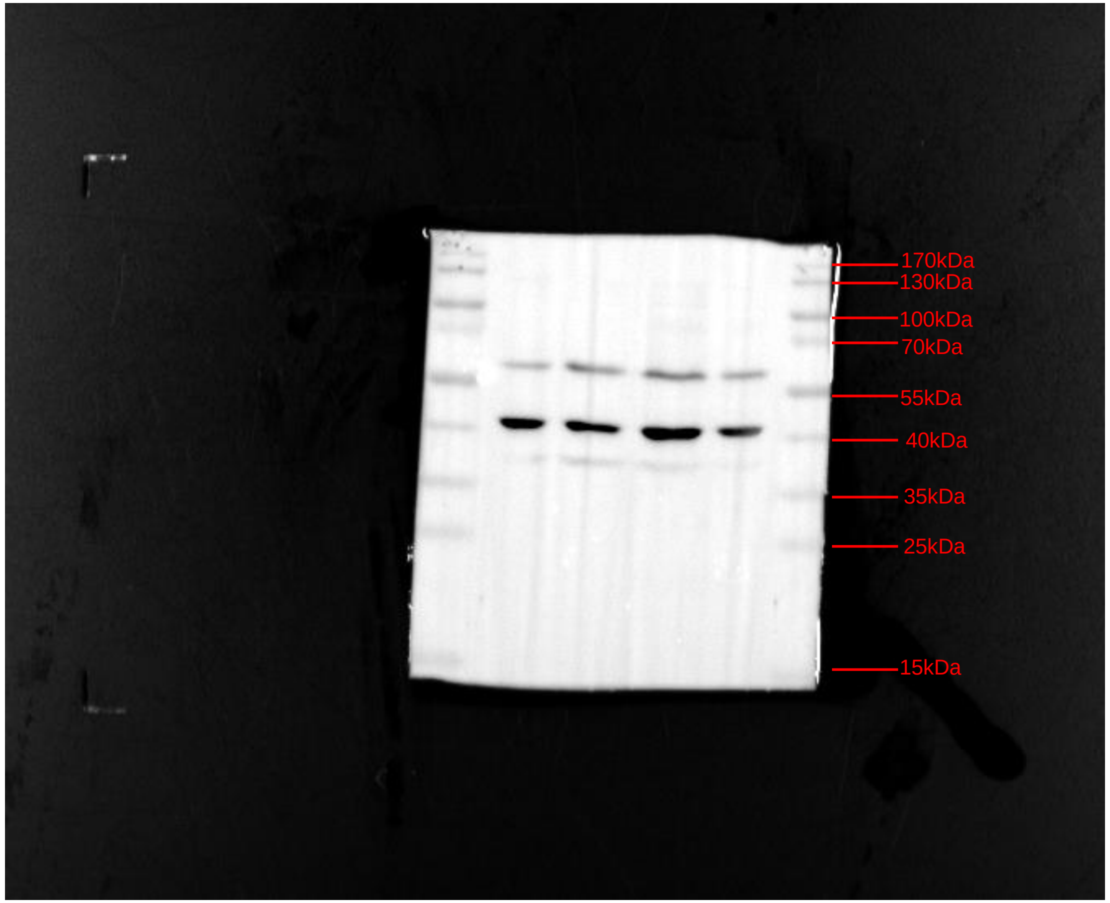

Figure 5h Transferrin HuCCT1

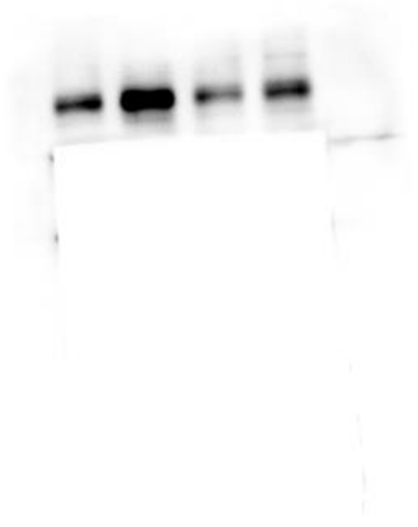

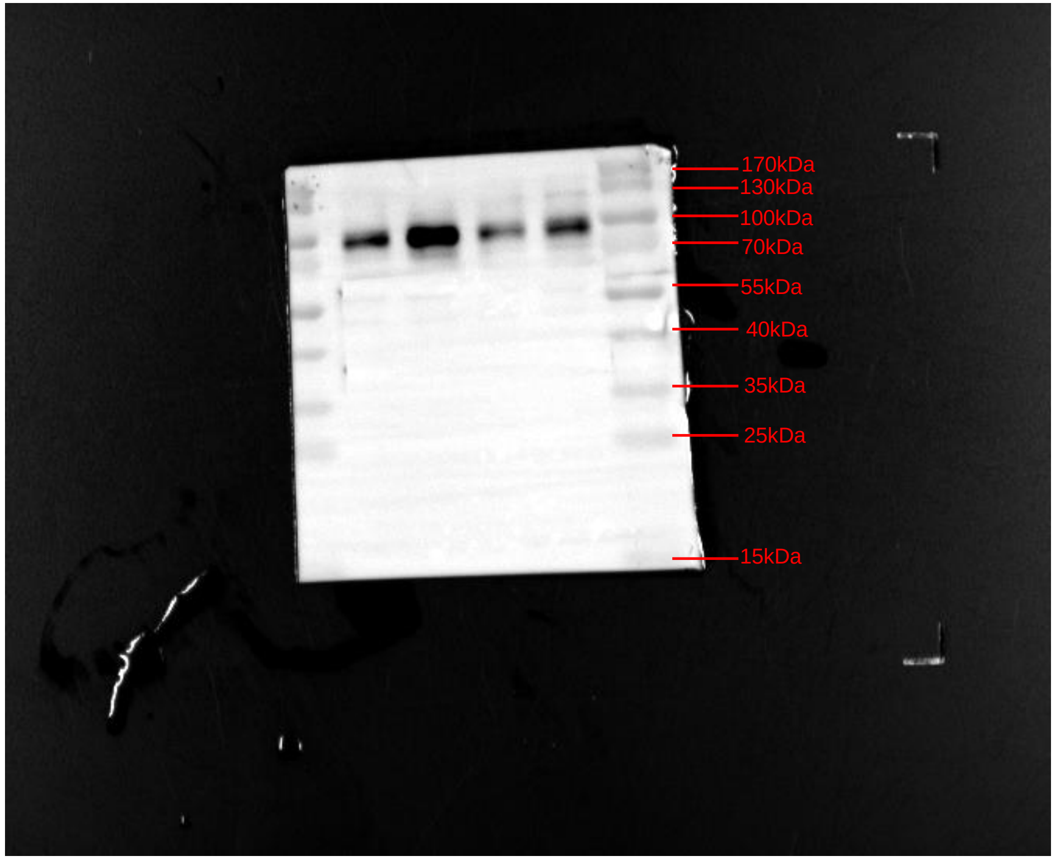

Figure 5h SLC7A11 HuCCT1

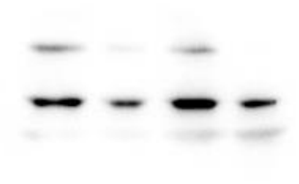

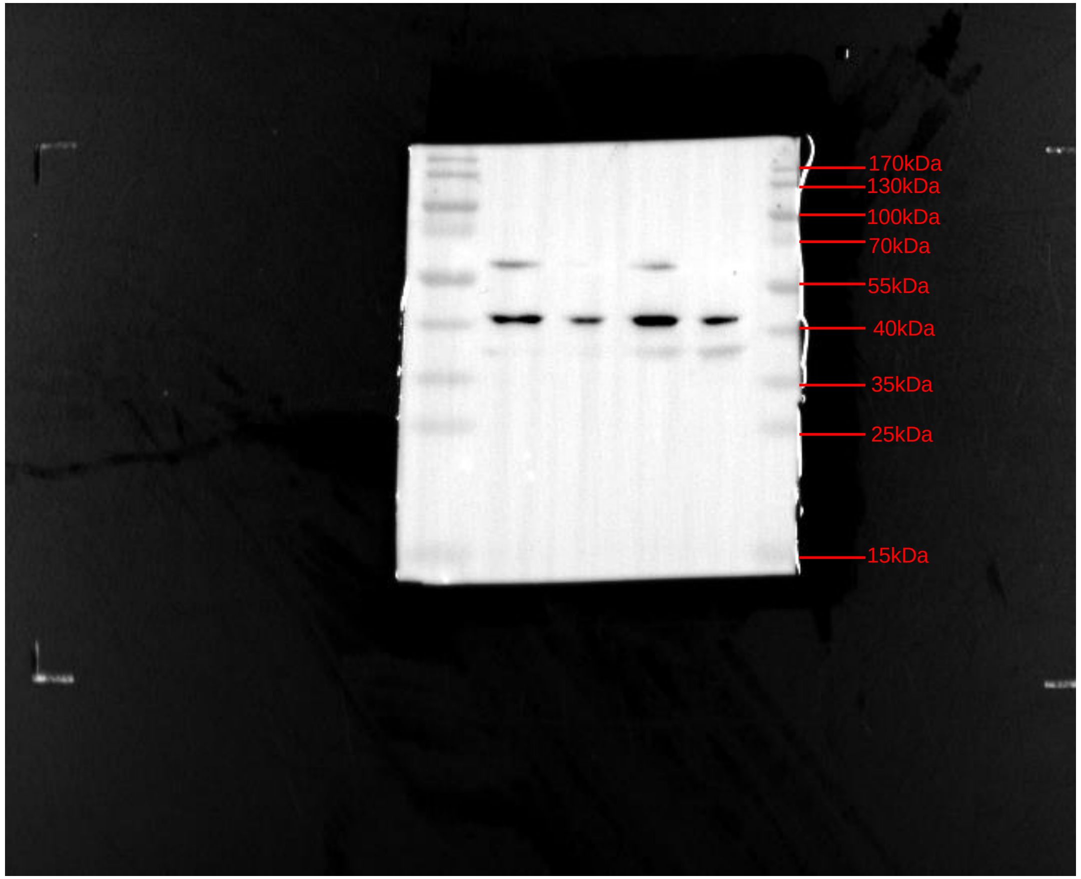

Figure 5h COX2 HuCCT1

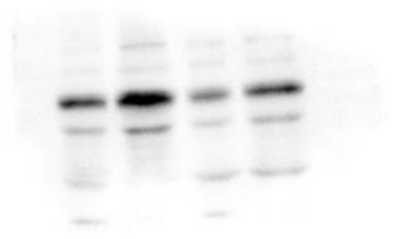

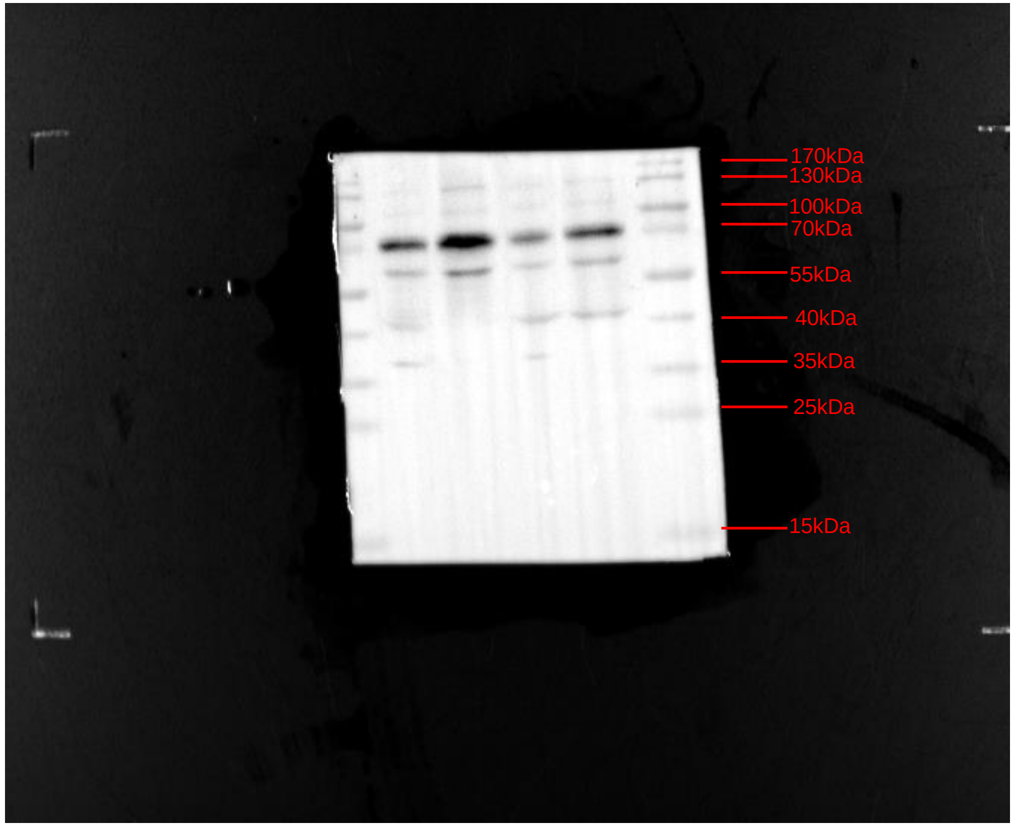

Figure 5h SLC40A1 HuCCT1

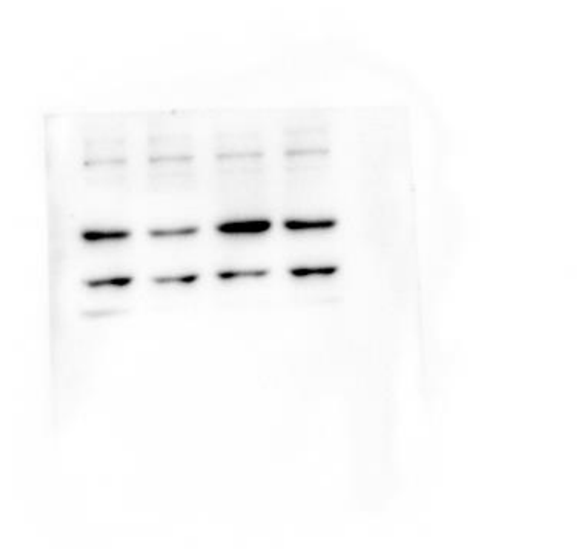

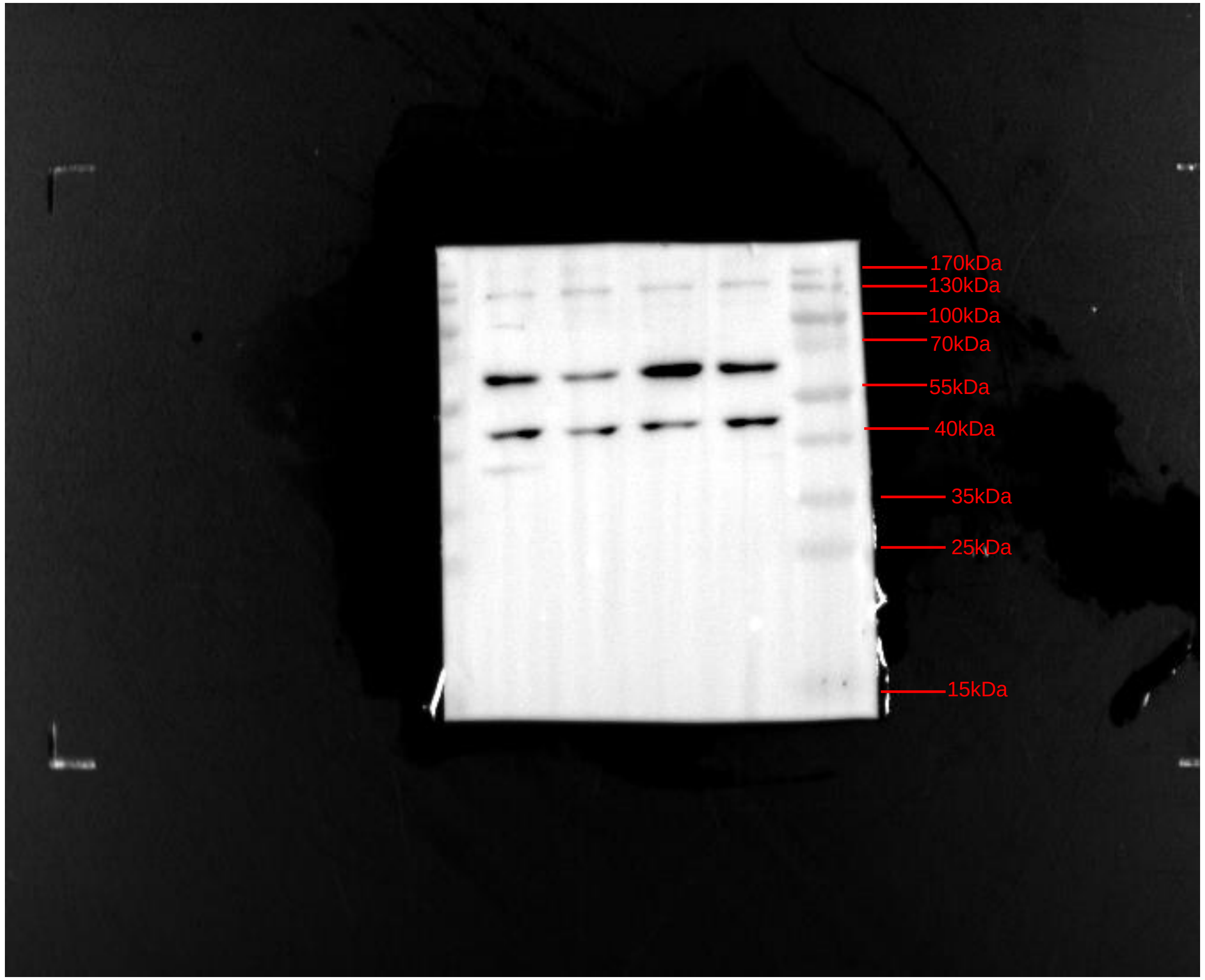

Figure 5h GPX4 HuCCT1

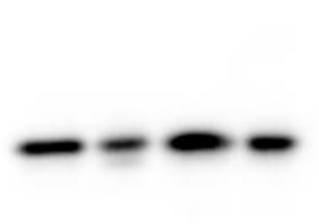

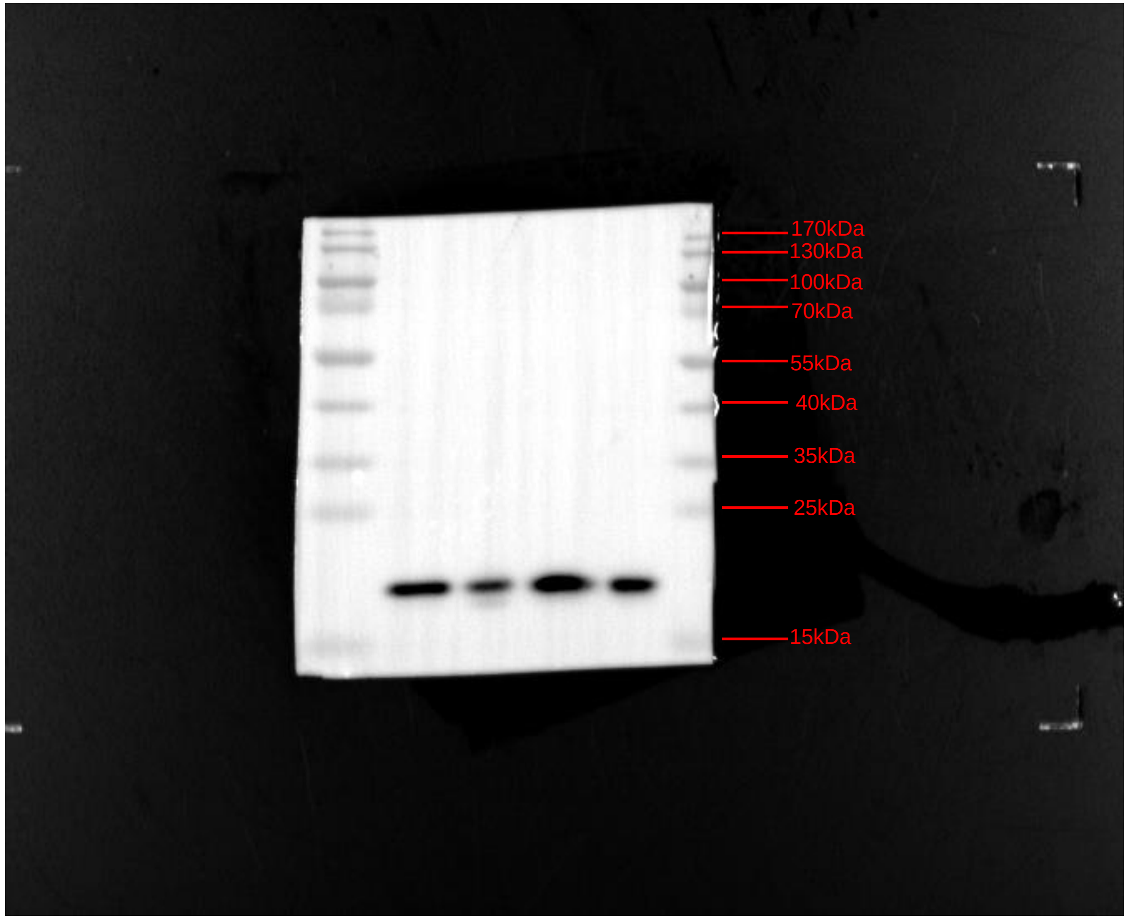

Figure 5h GPADH HuCCT1

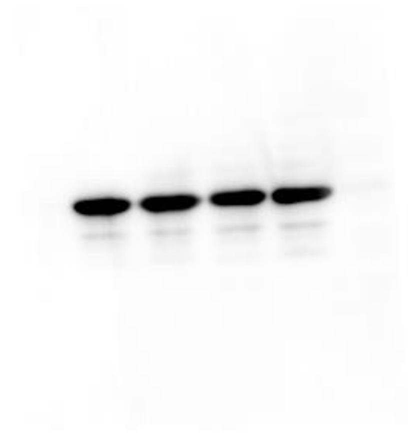

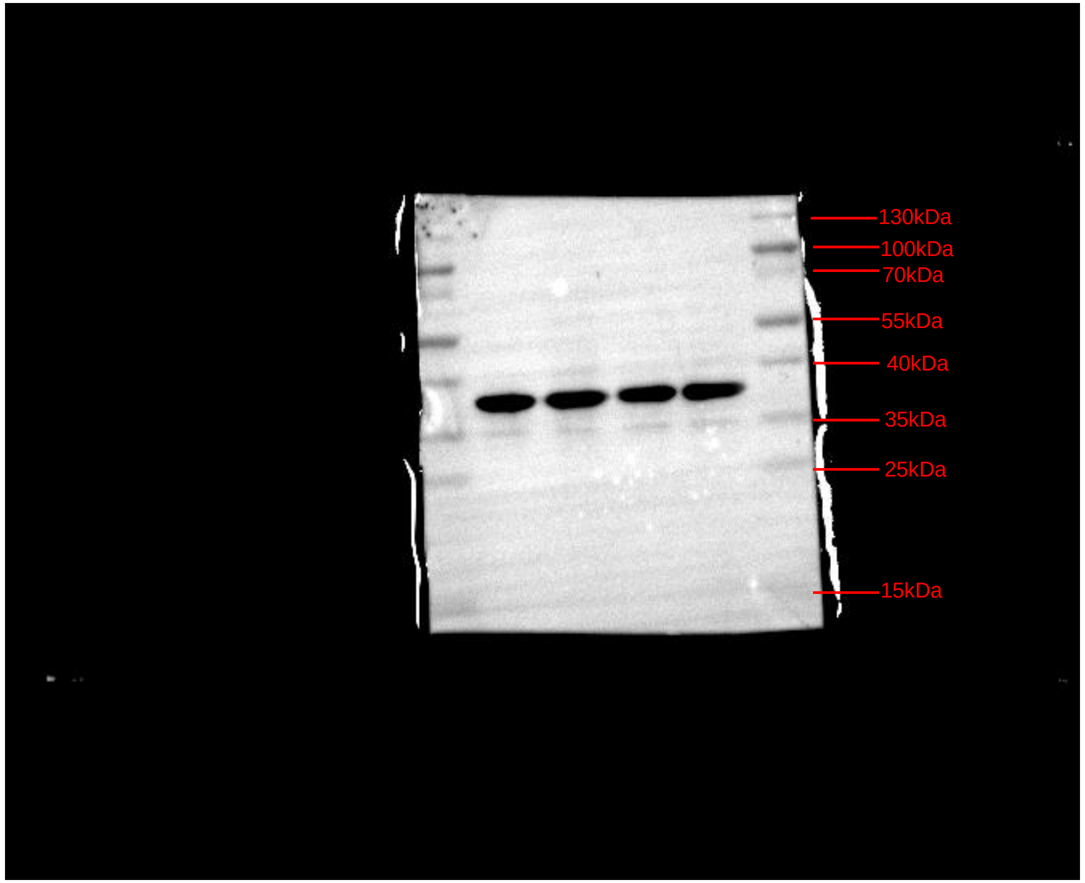

Figure 5h E-cadherin RBE

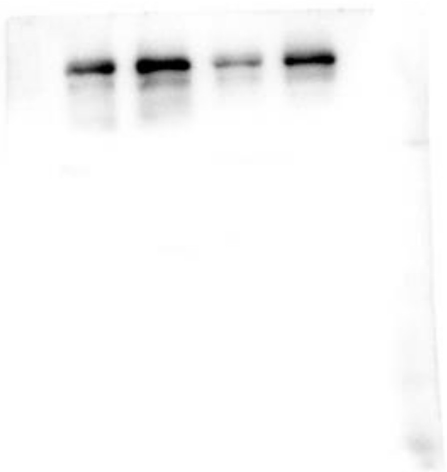

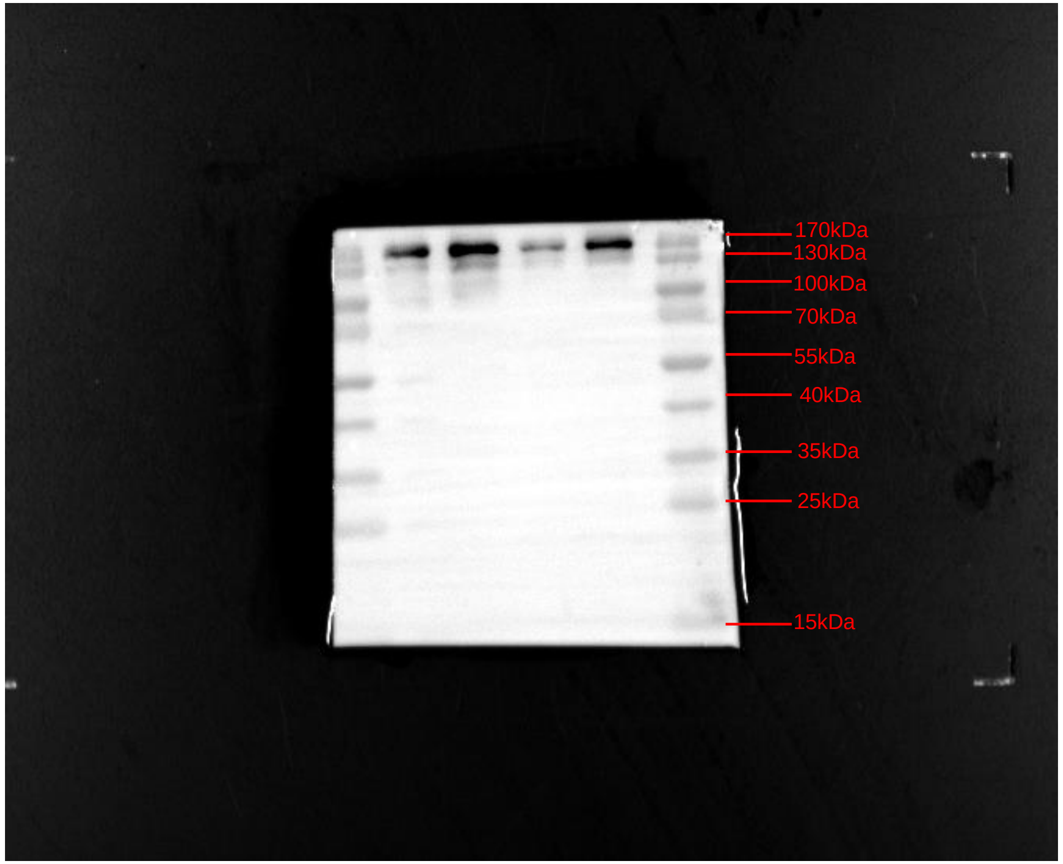

Figure 5h N-cadherin RBE

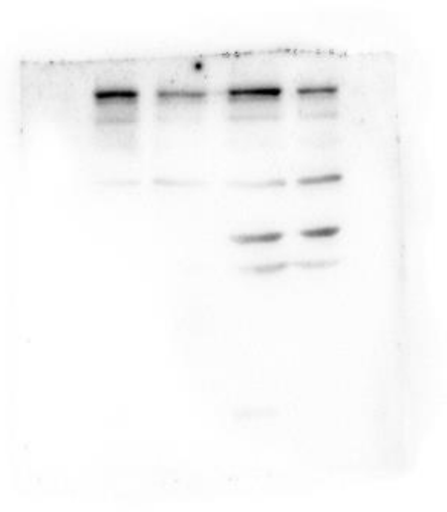

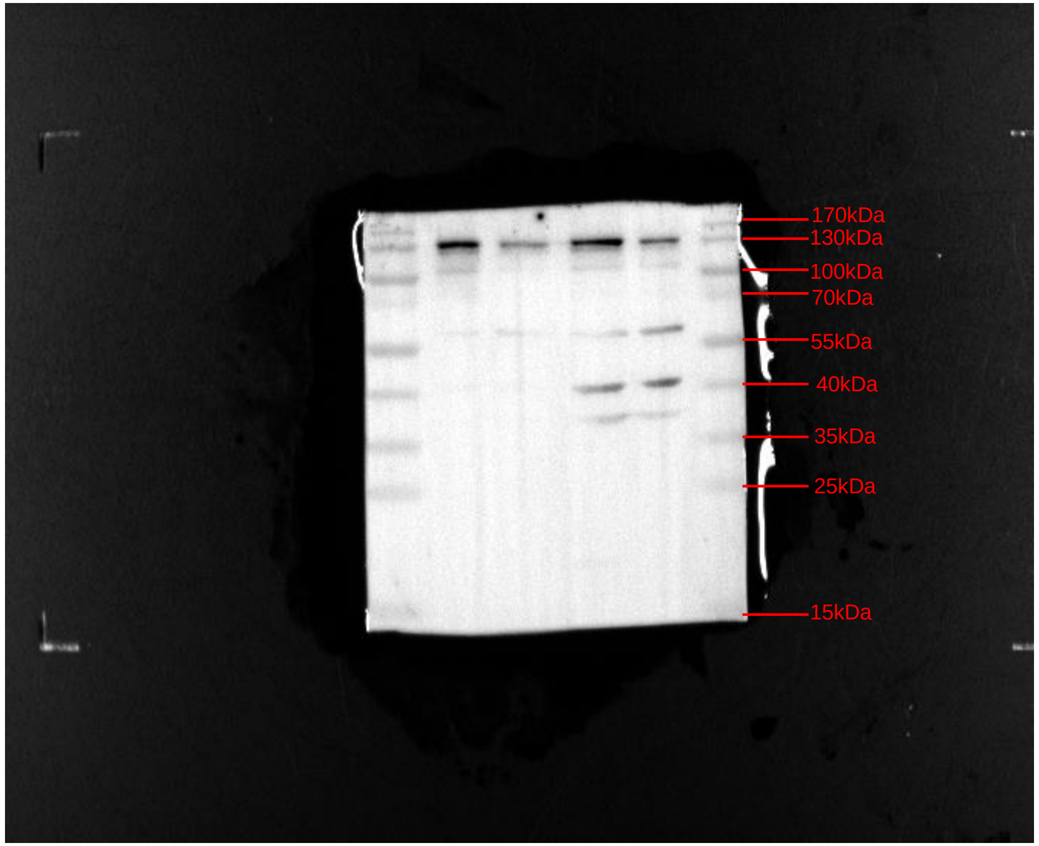

Figure 5h Vimentin RBE

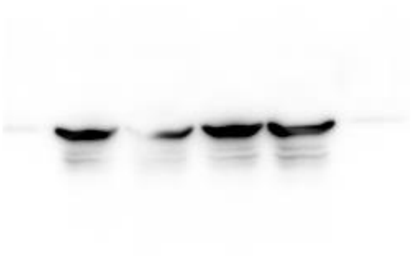

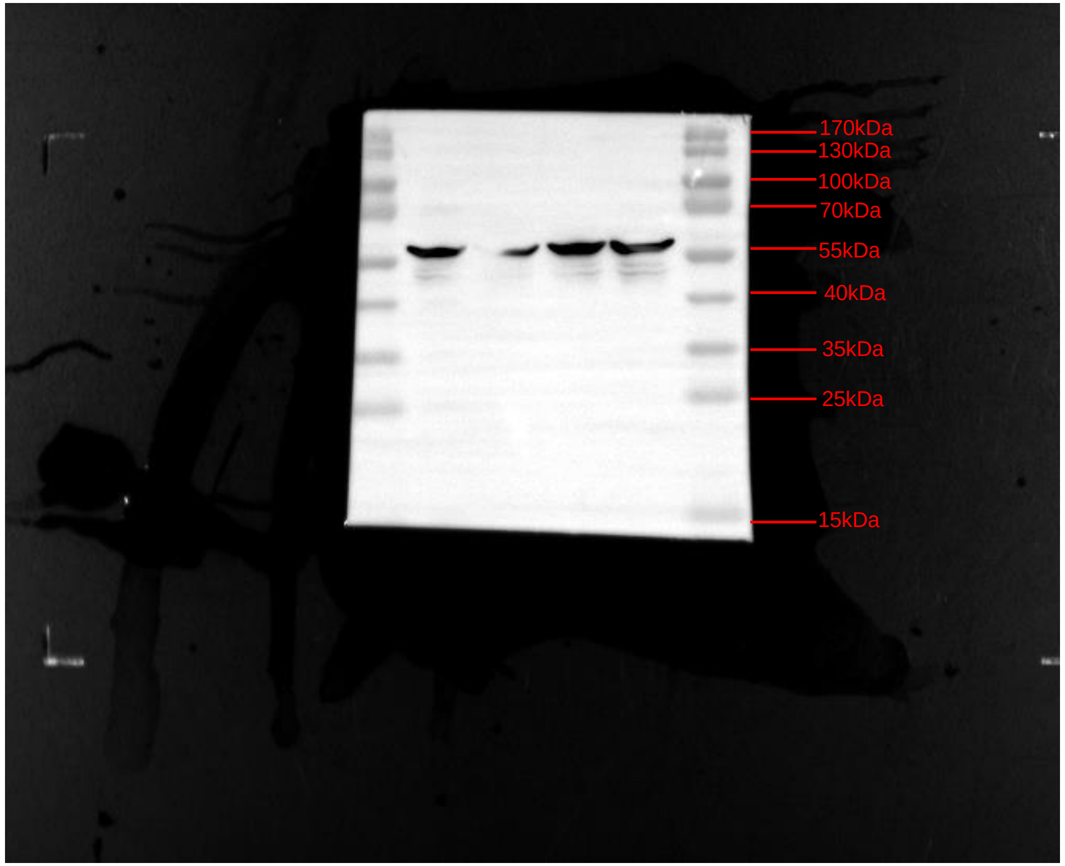

Figure 5h Transferrin RBE

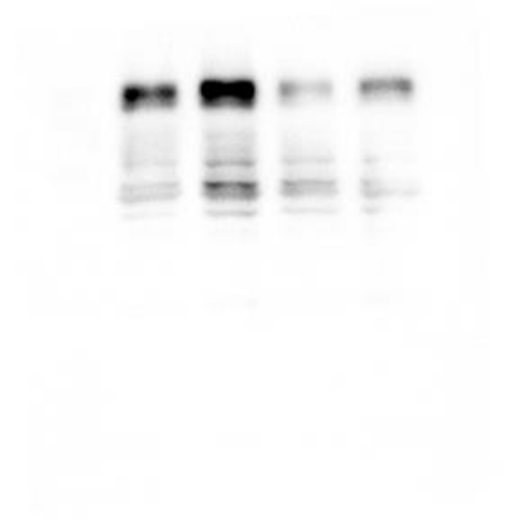

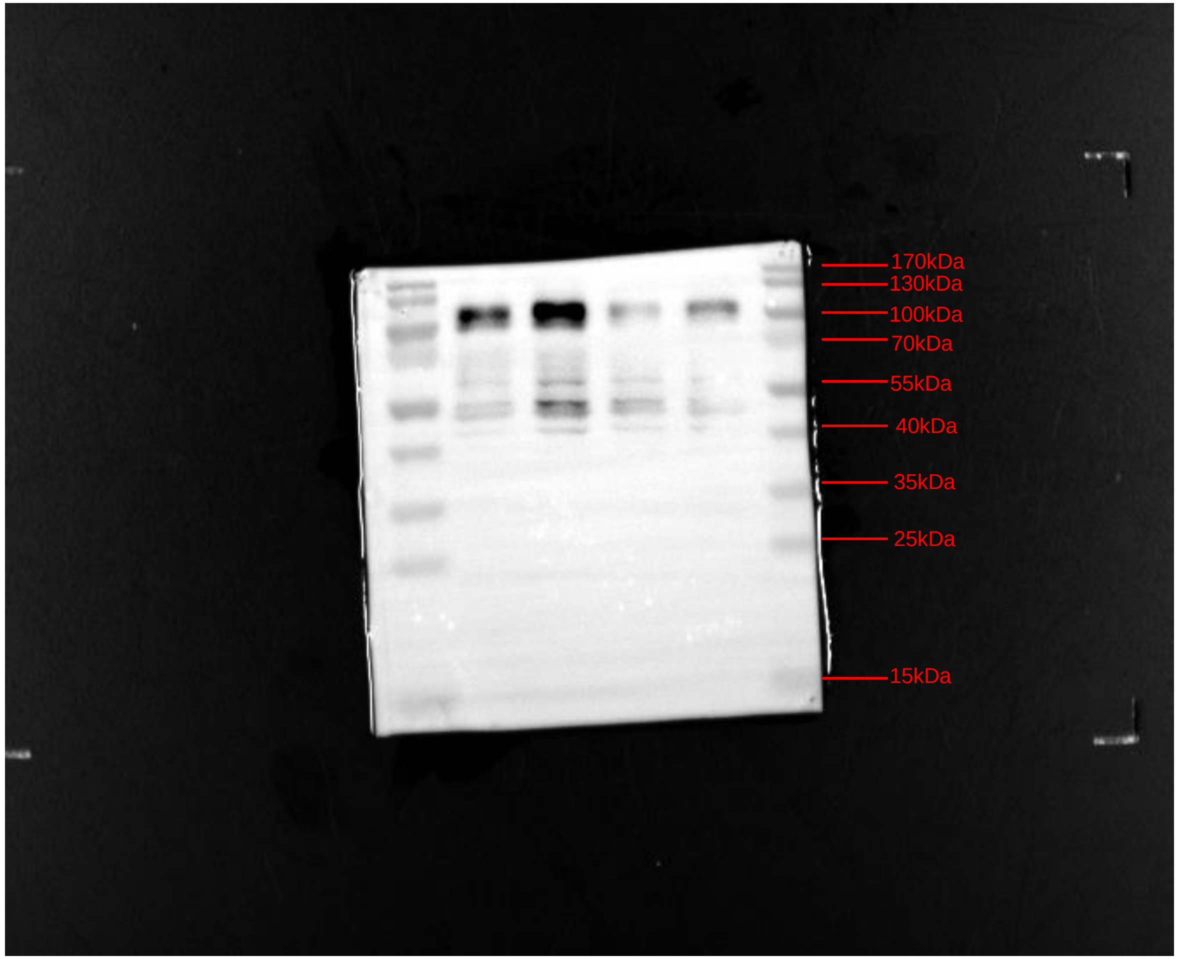

Figure 5h SLC7A11 RBE

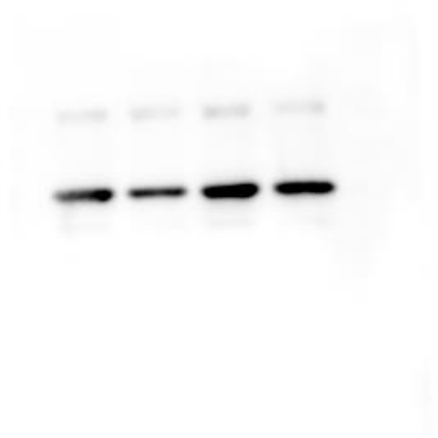

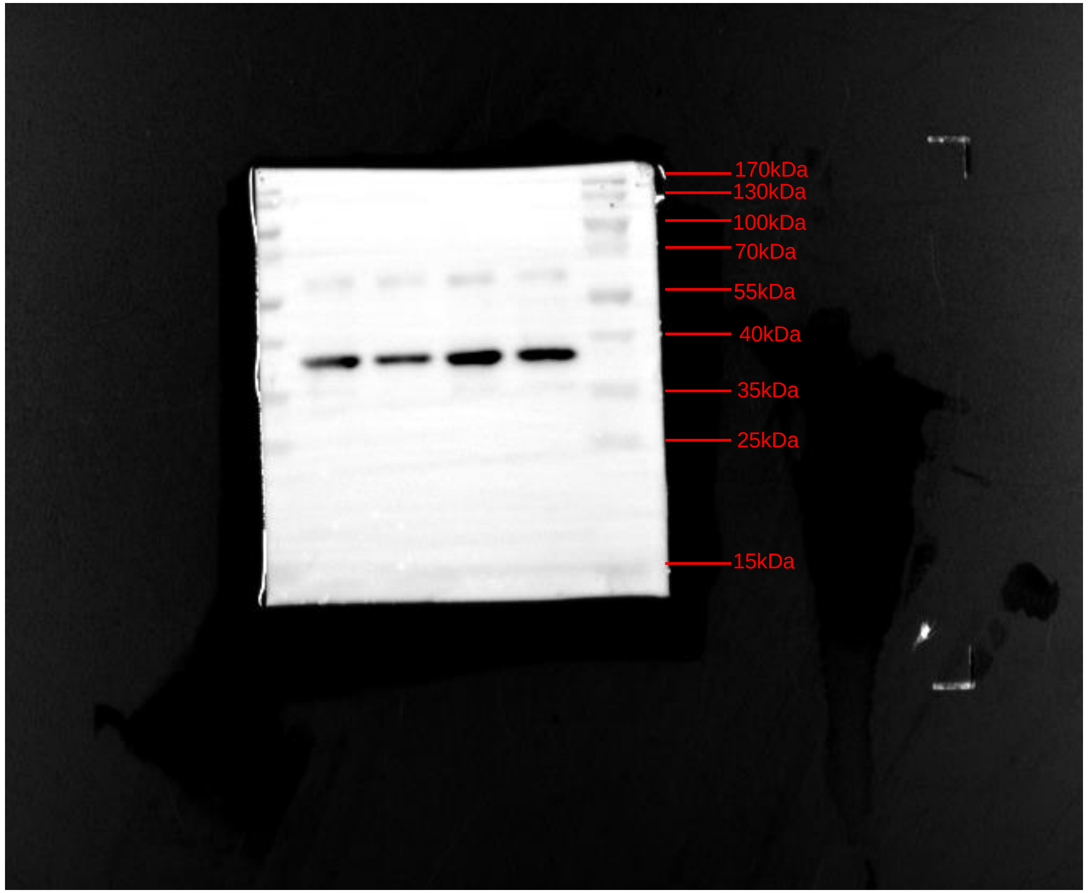

Figure 5h COX2 RBE

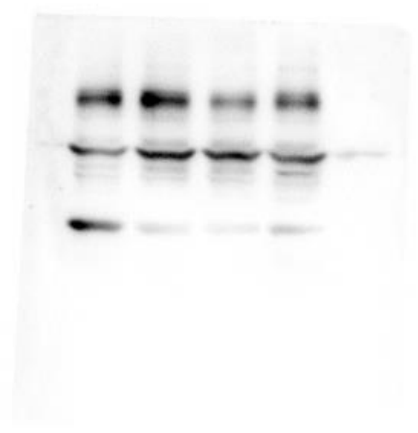

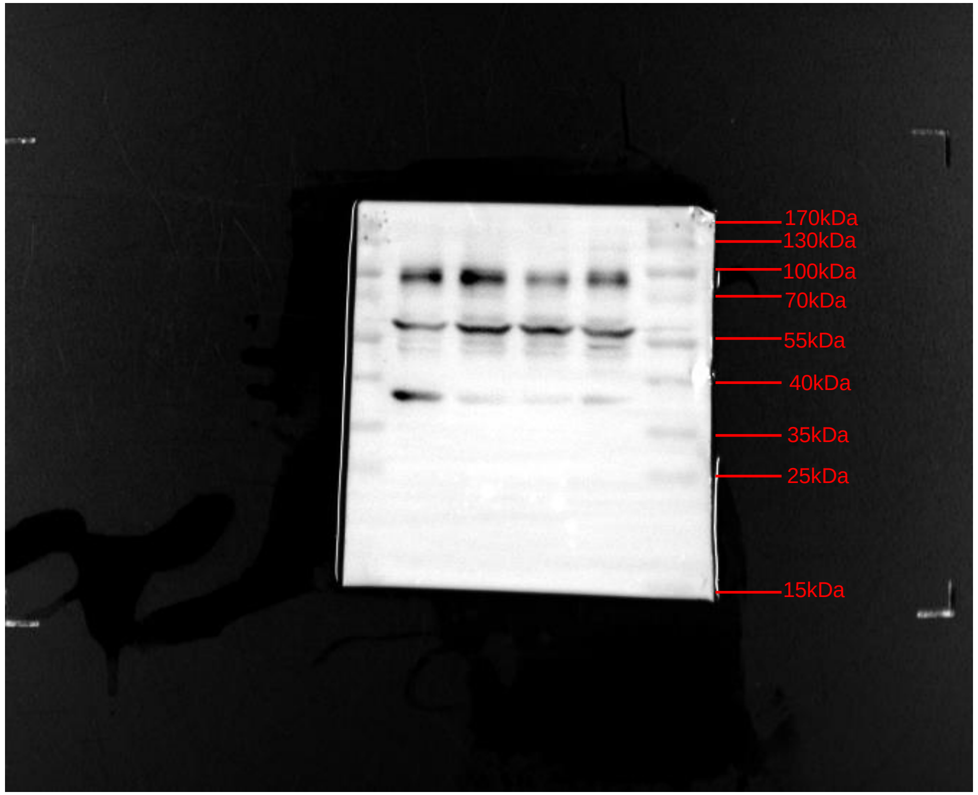

Figure 5h SLC40A1 RBE

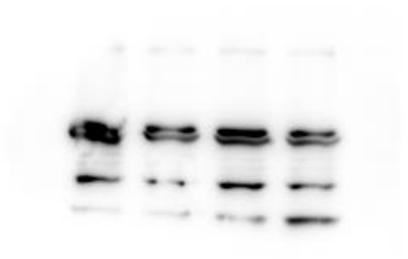

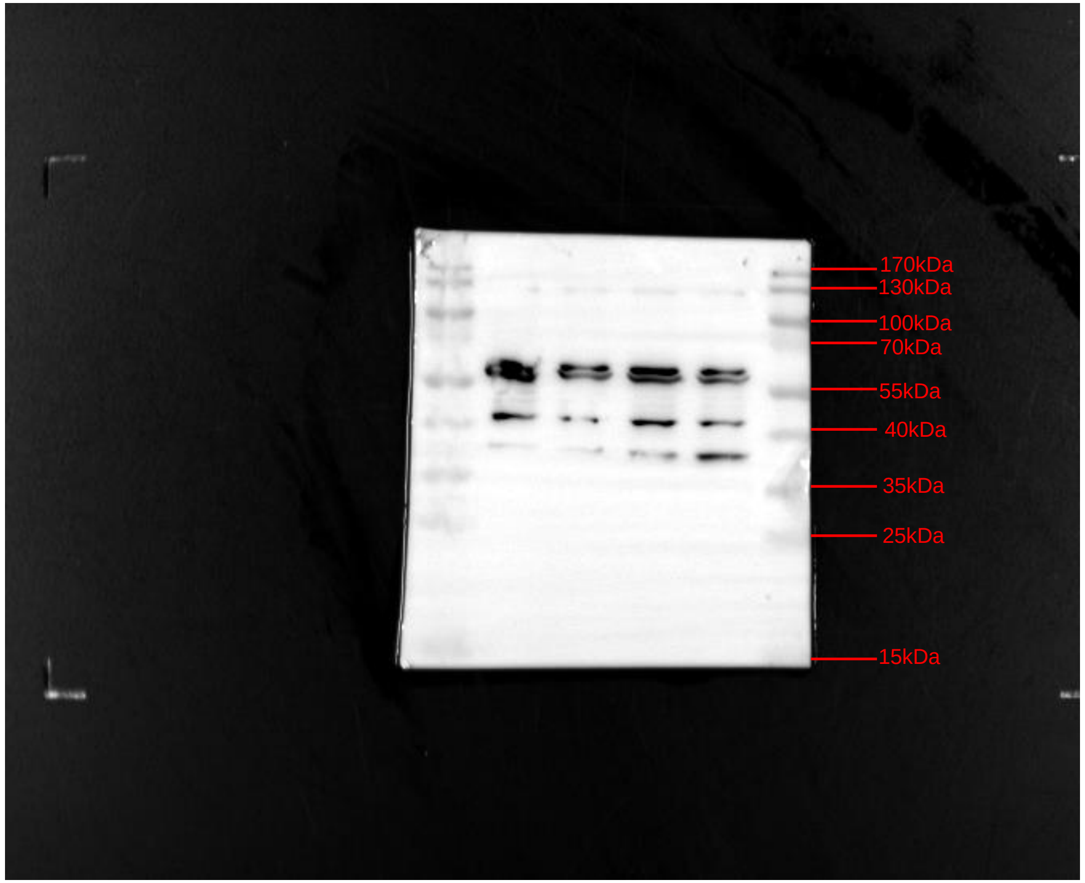

Figure 5h GPX4 REB

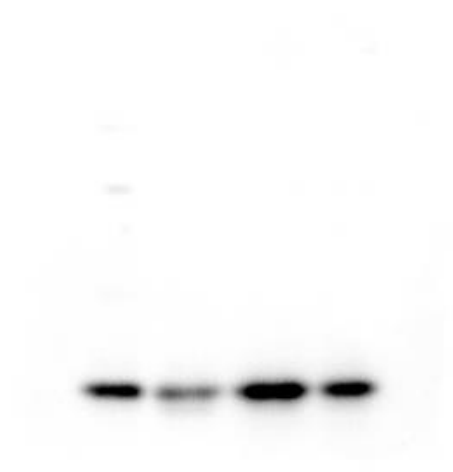

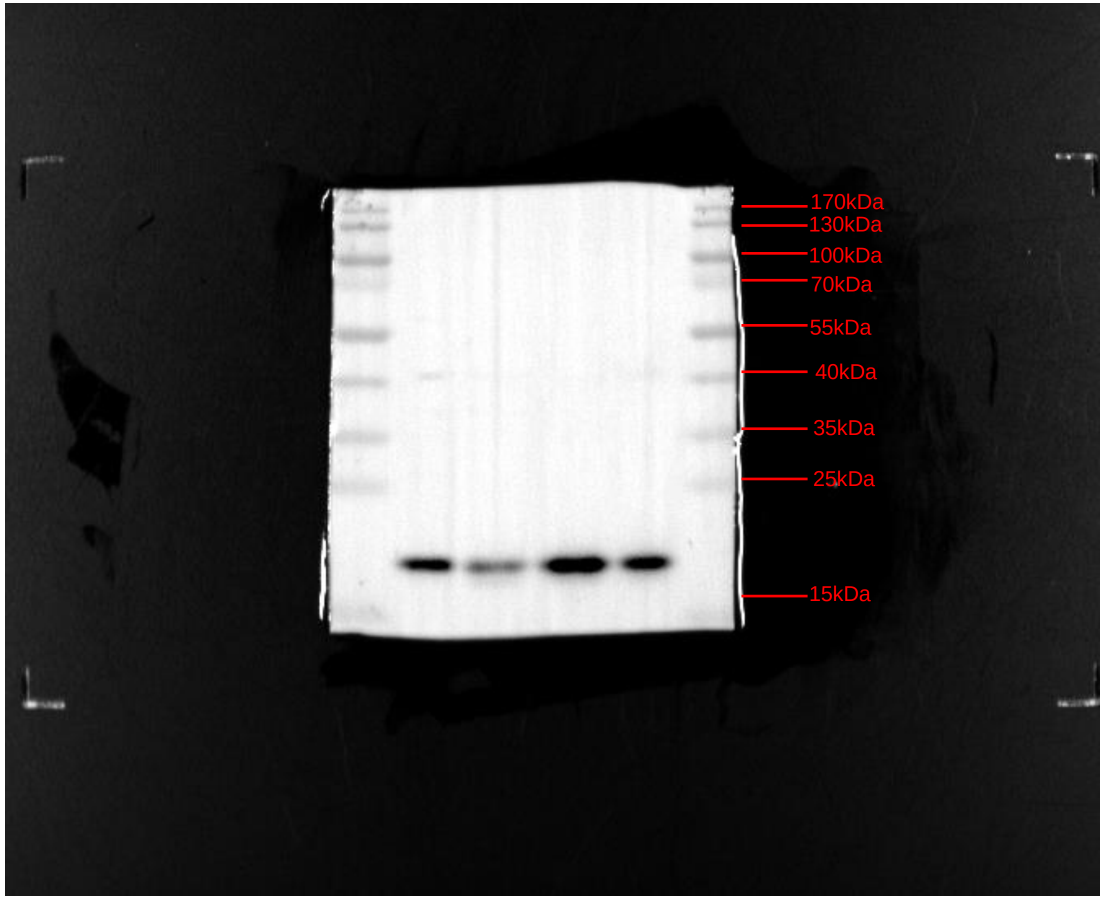

Figure 5h GPADH RBE

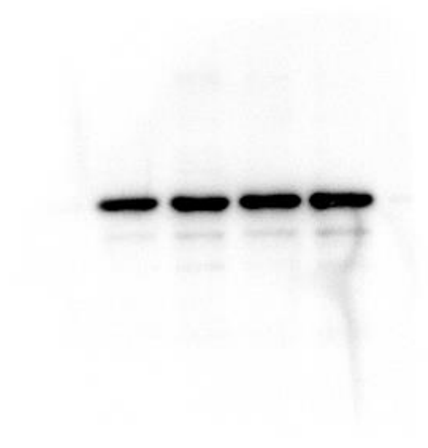

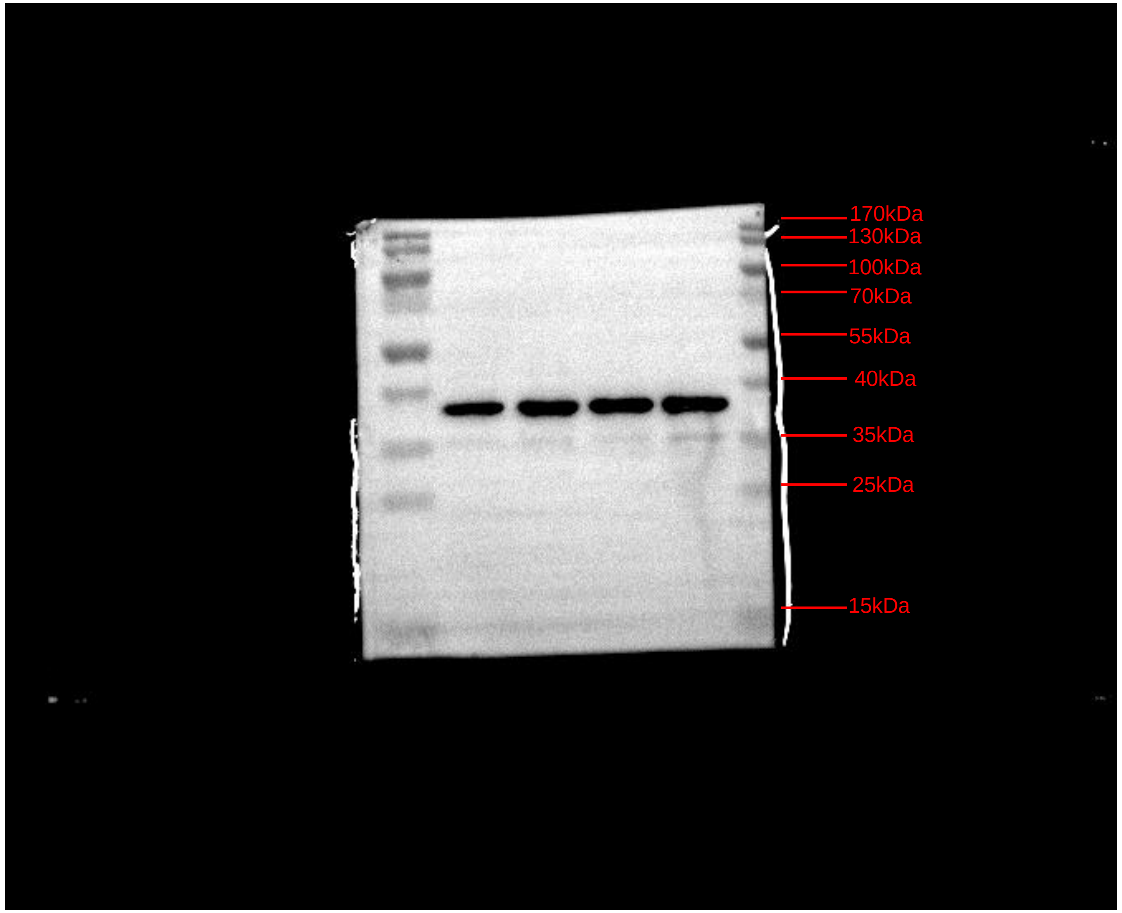

Figure 7h N-cadherin HuCCT1

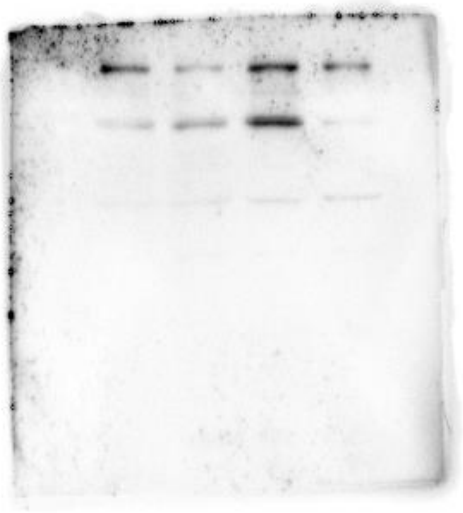

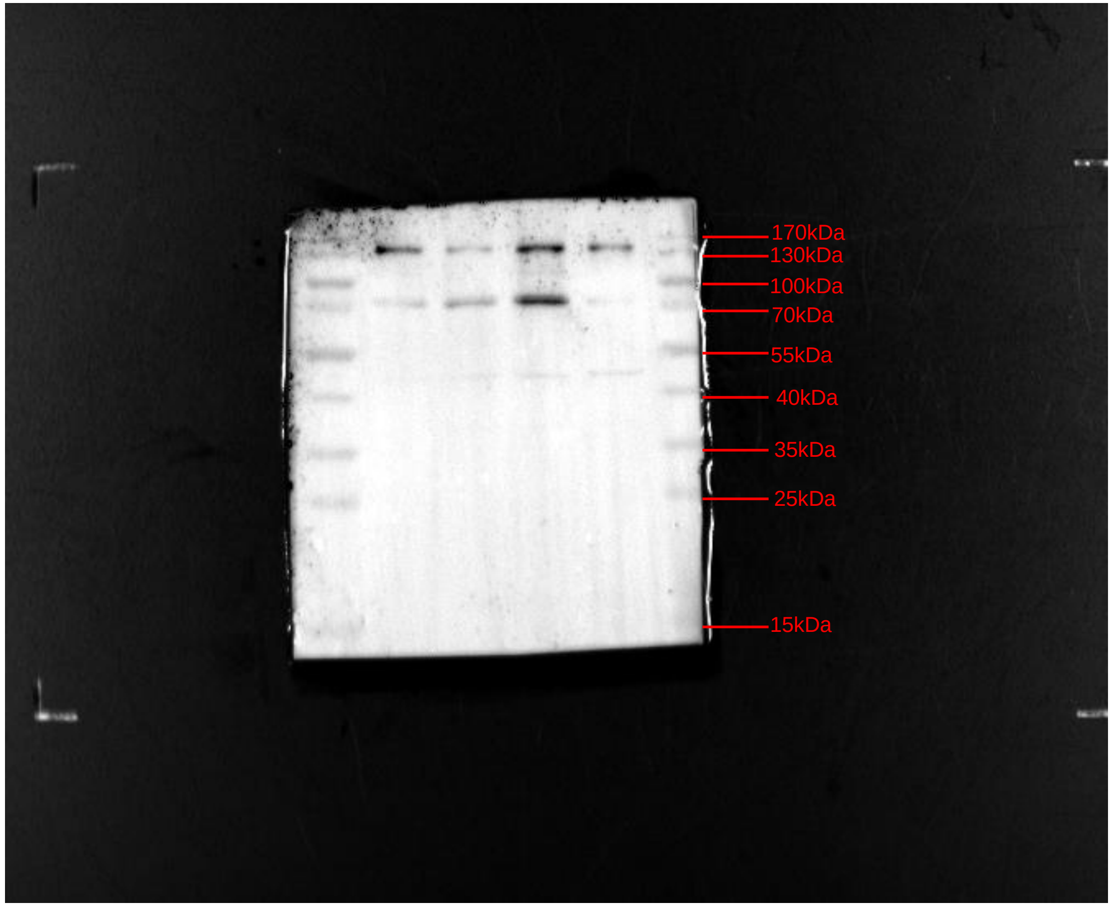

Figure 7h E-cadherin HuCCT1

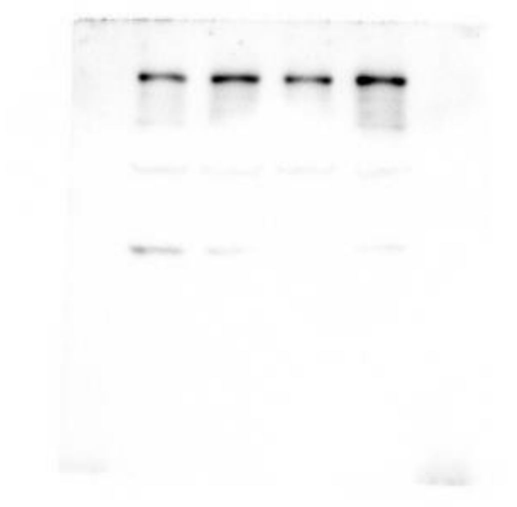

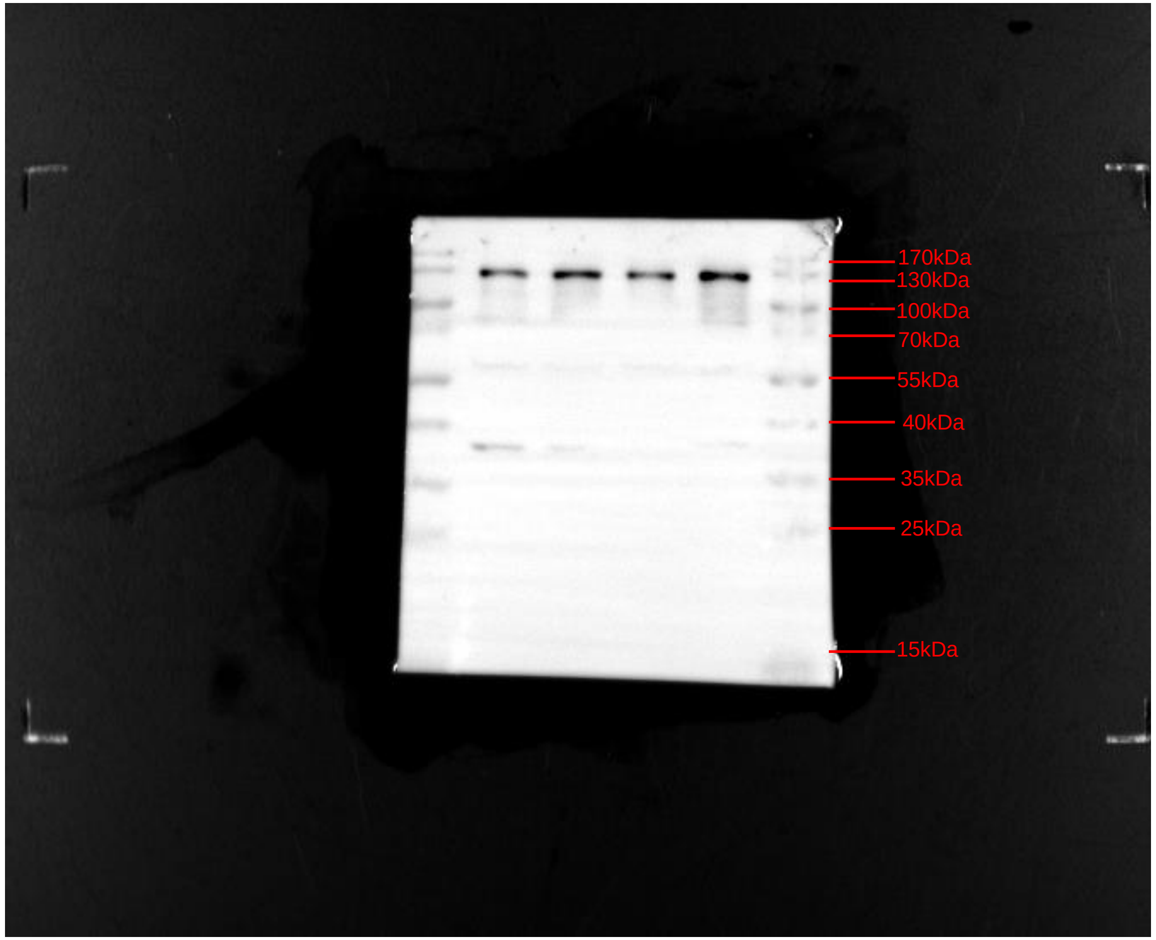

Figure 7h Vimentin HuCCT1

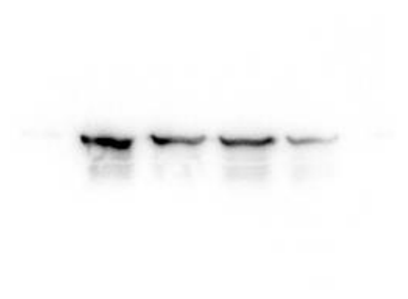

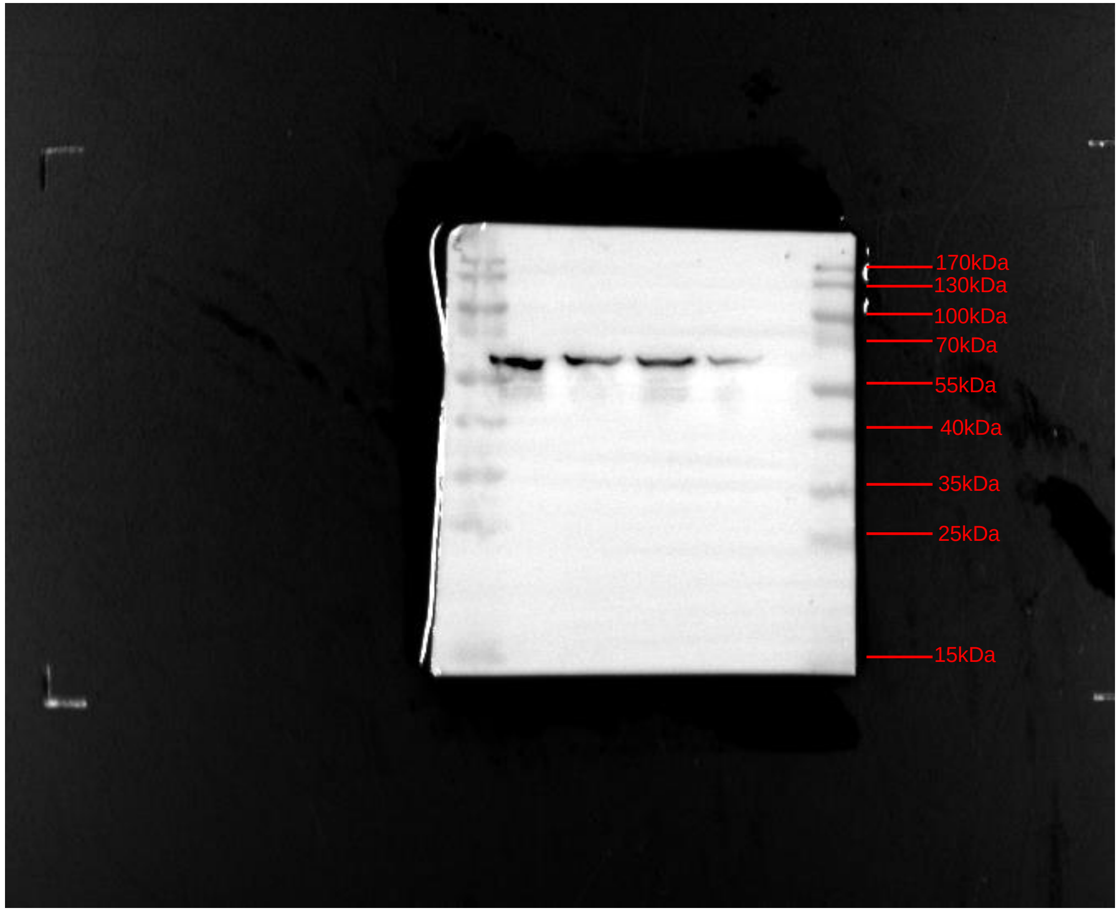

Figure 7h Transferrin HuCCT1

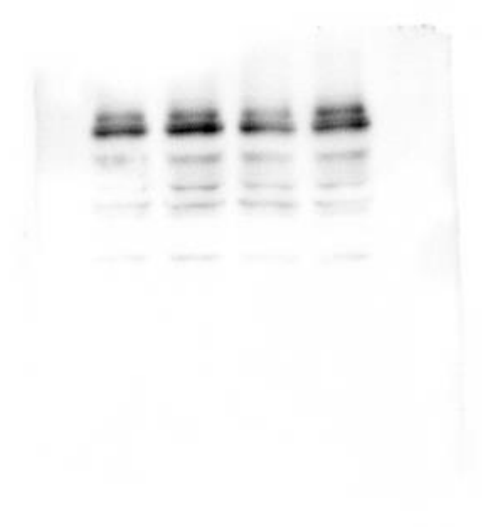

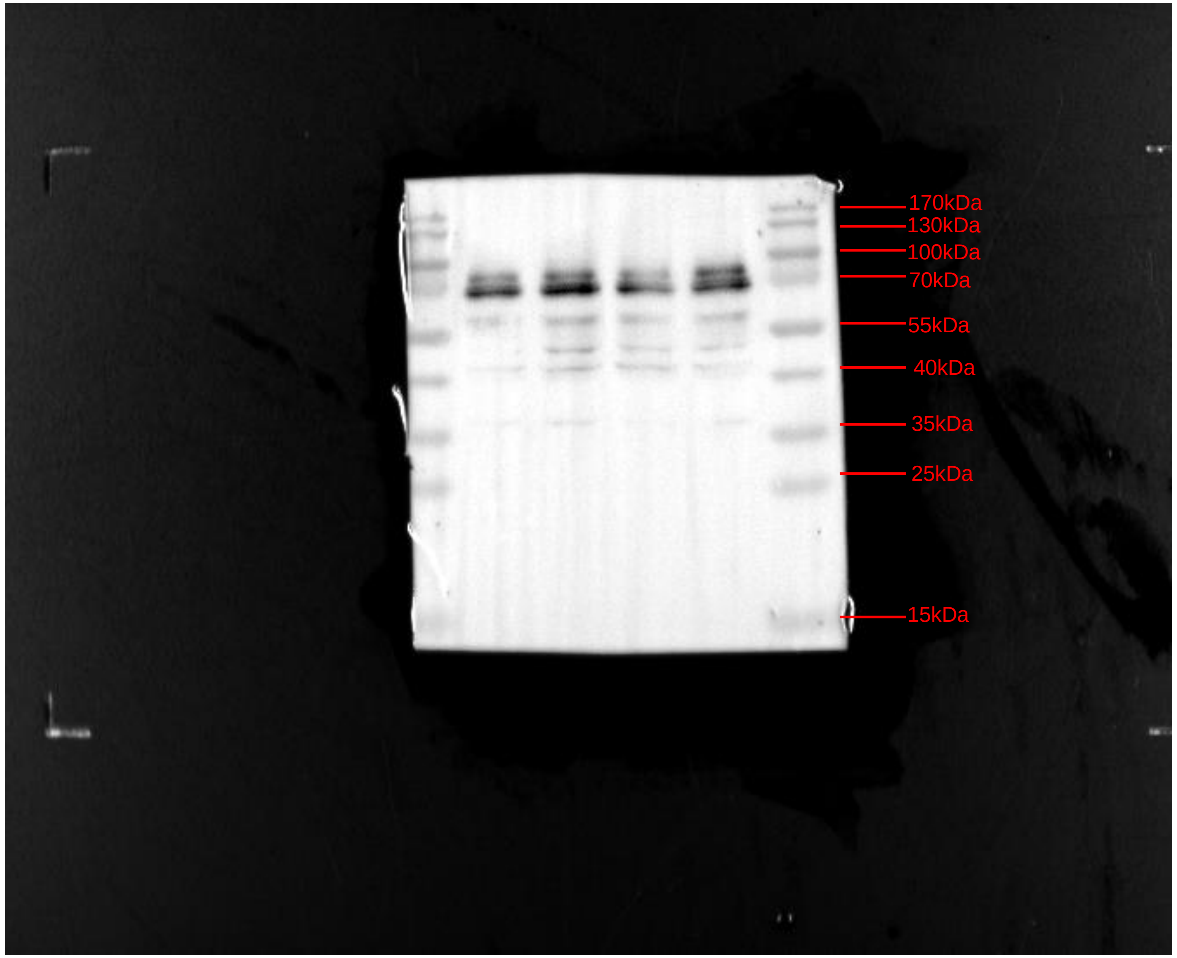

Figure 7h SLC40A1 HuCCT1

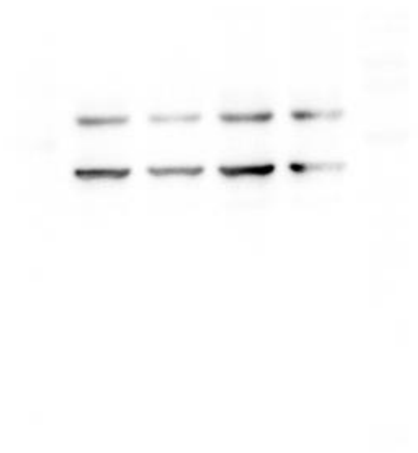

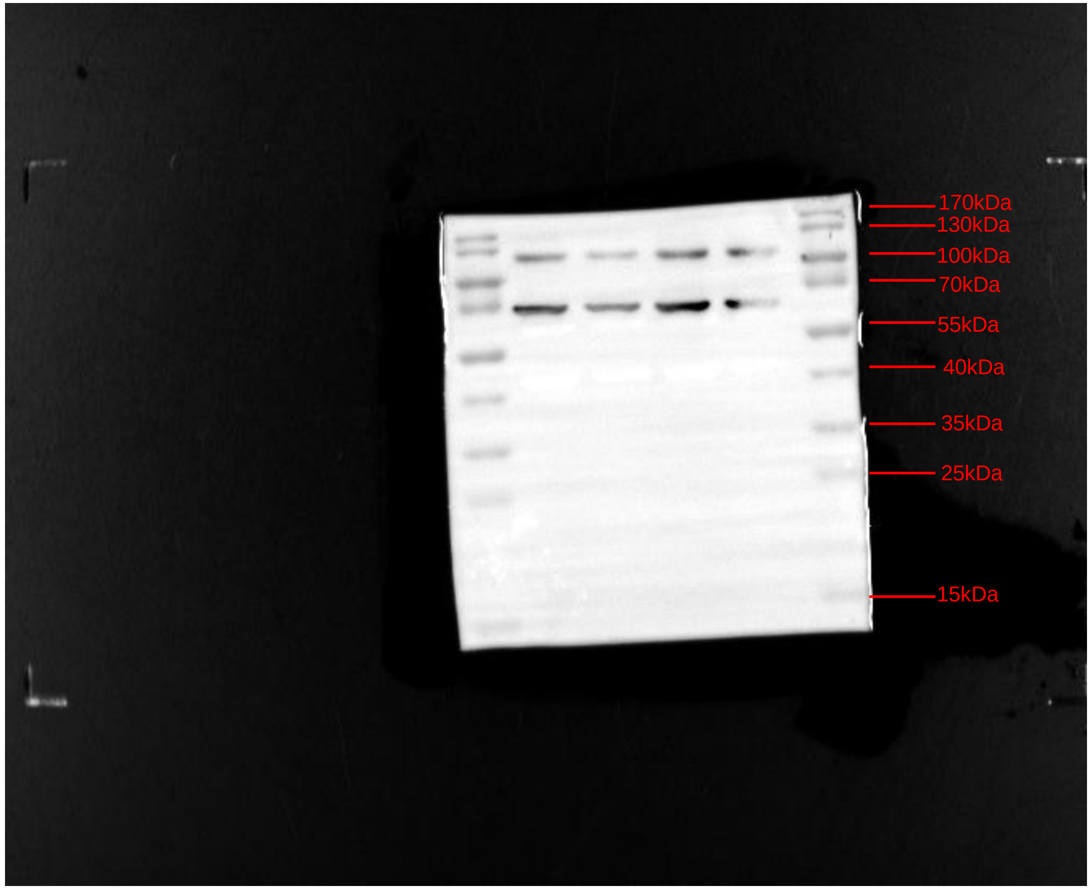

Figure 7h SLC7A11 HuCCT1

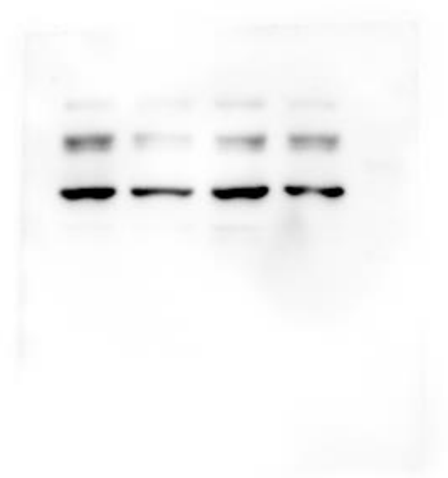

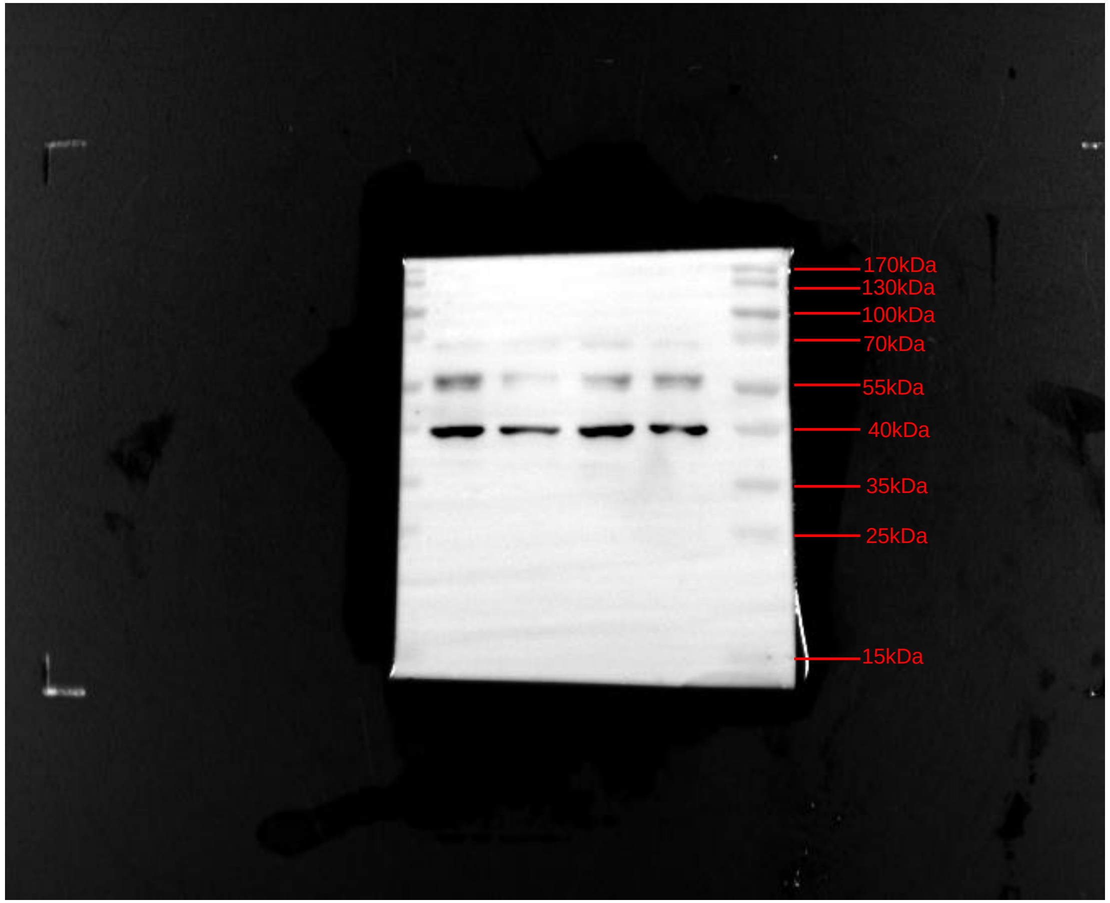

Figure 7h COX2 HuCCT1

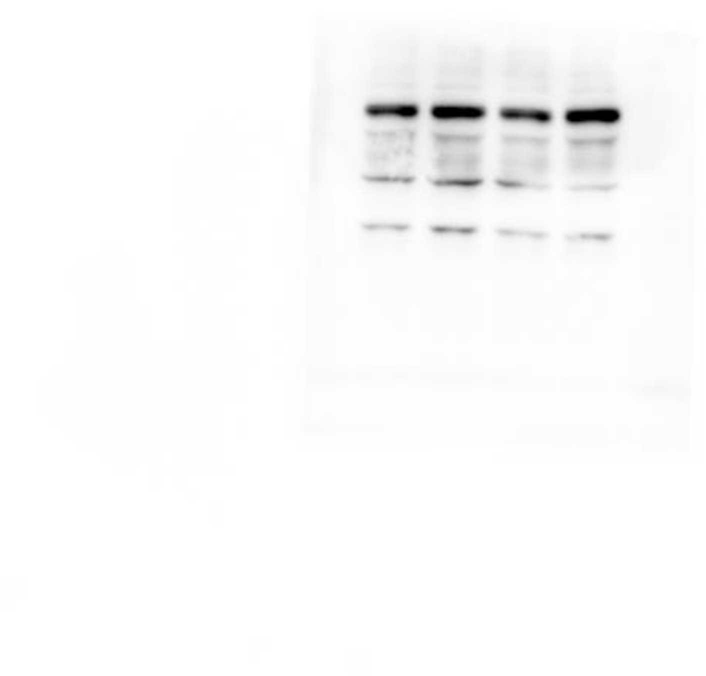

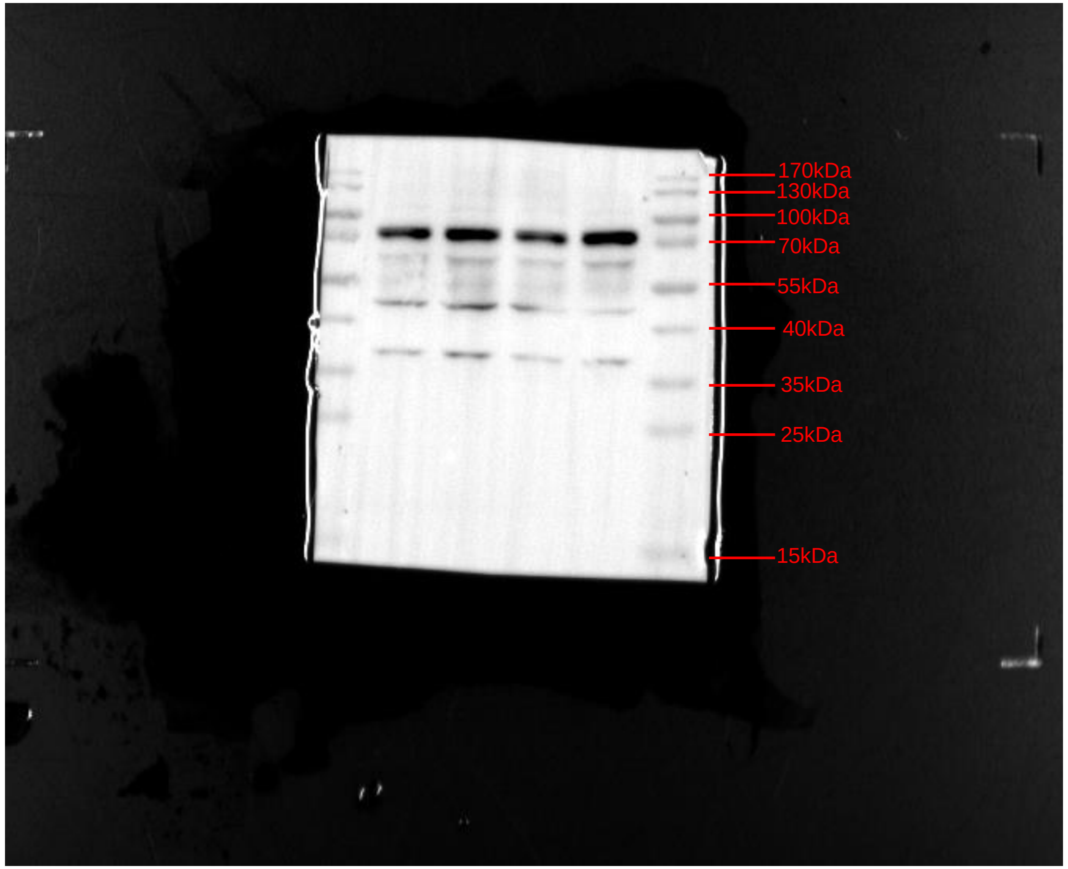

Figure 7h GPX4 HuCCT1

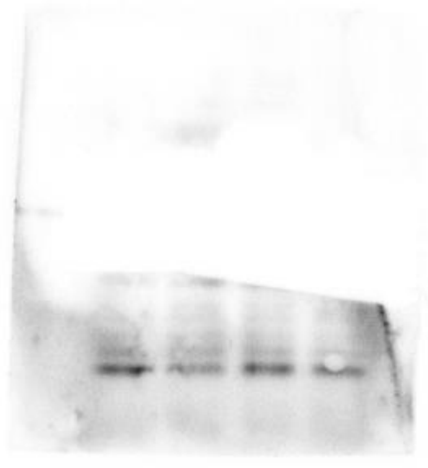

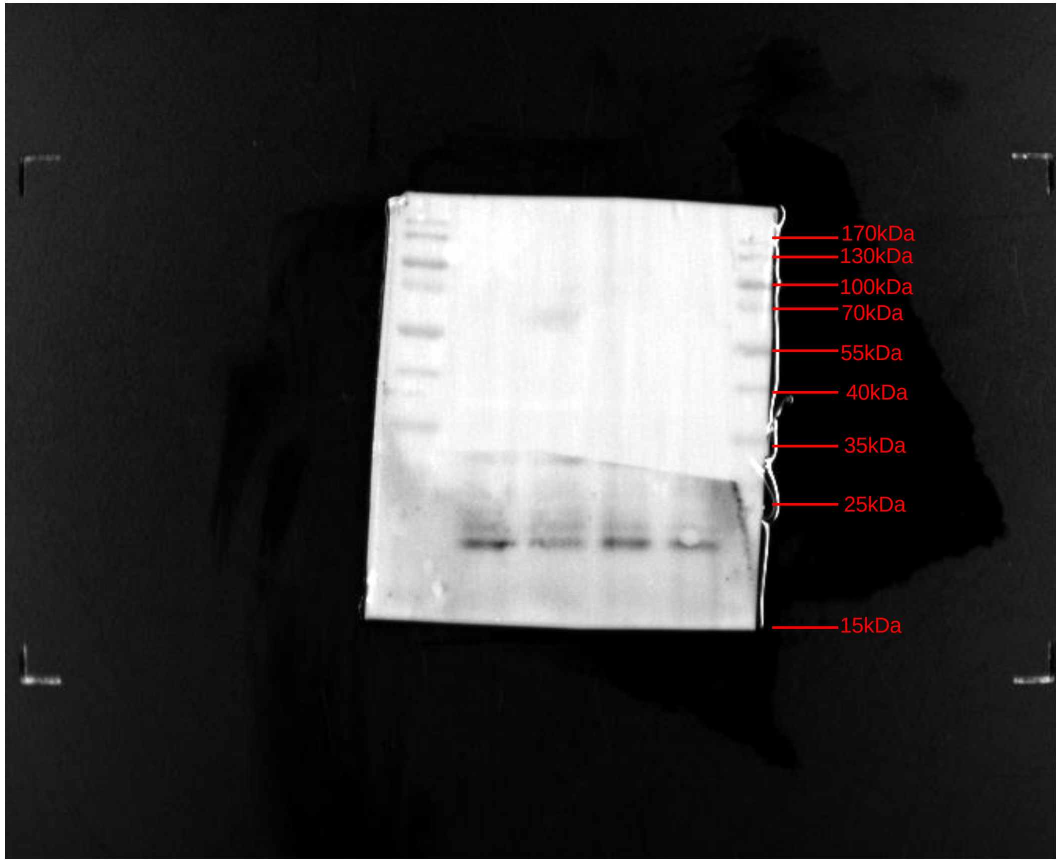

Figure 7h GPADH HuCCT1

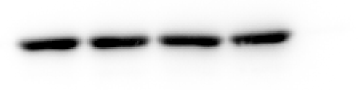

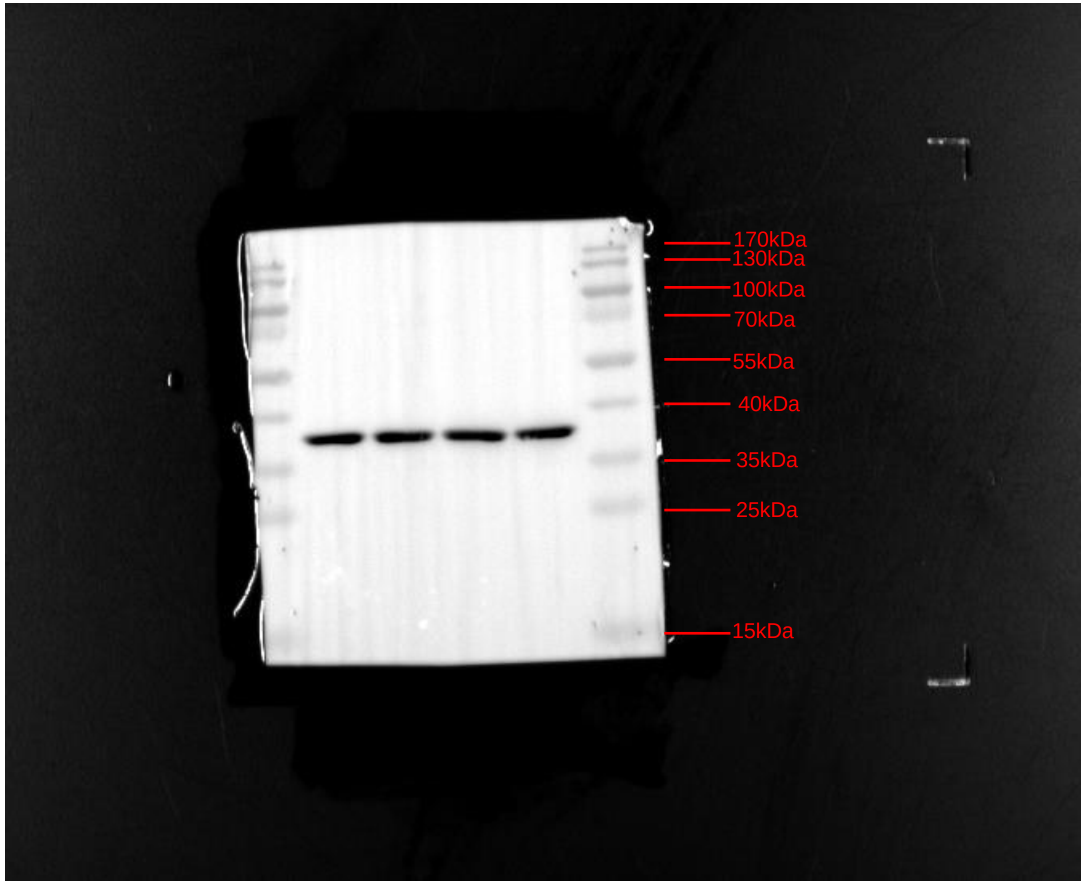

Figure 7h N-cadherin RBE

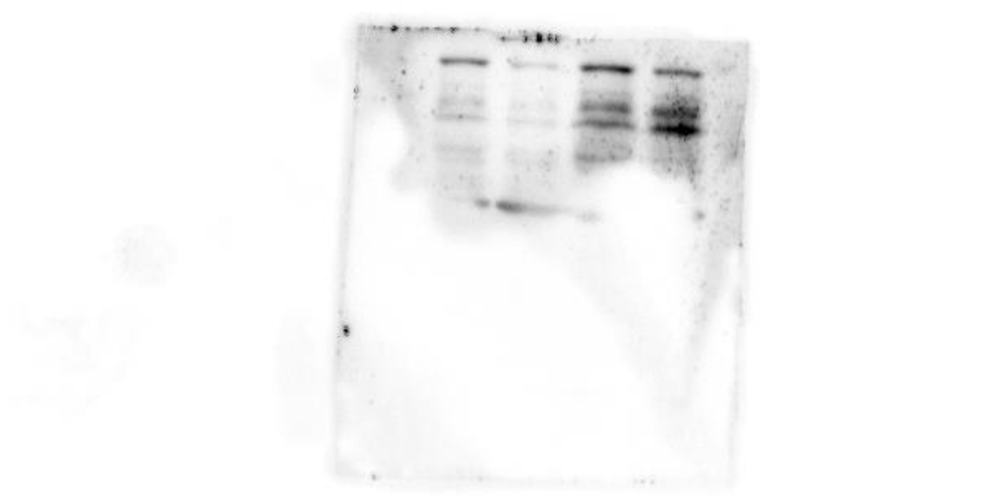

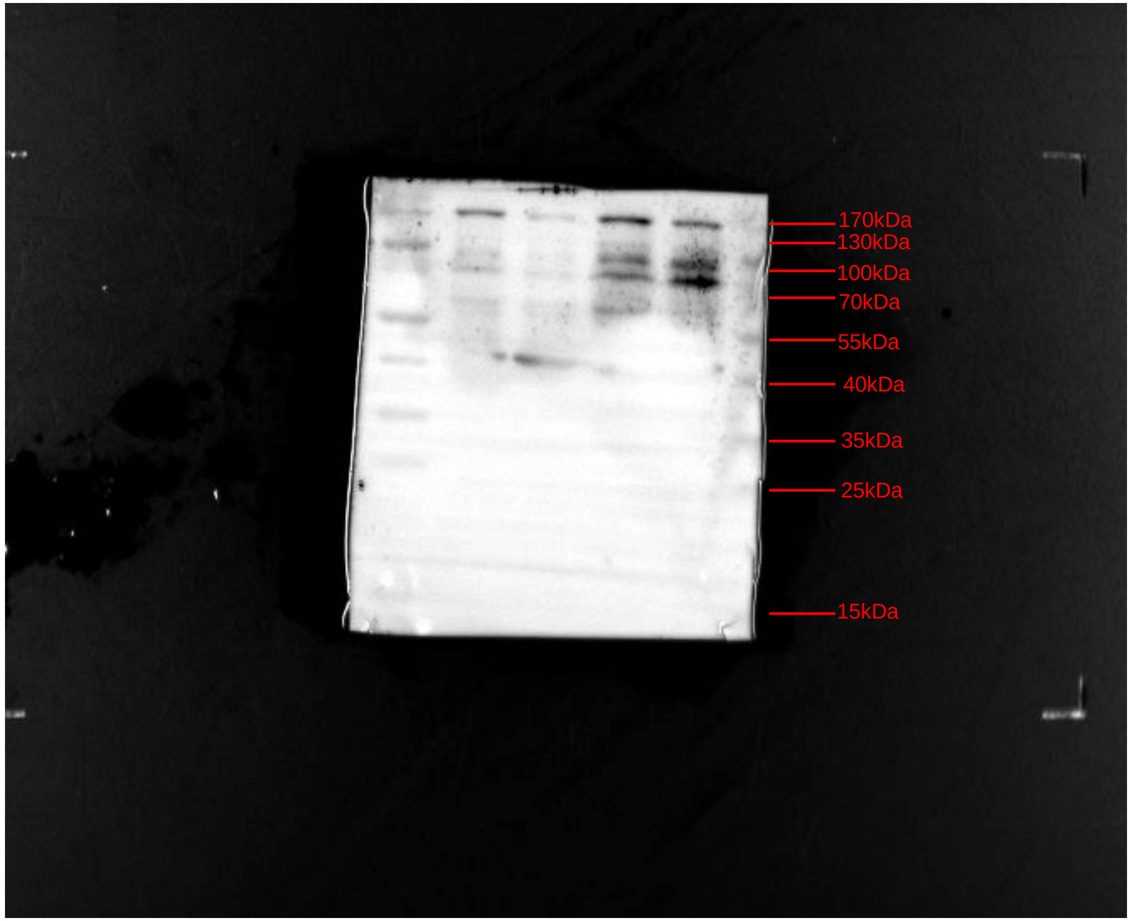

Figure 7h E-cadherin RBE

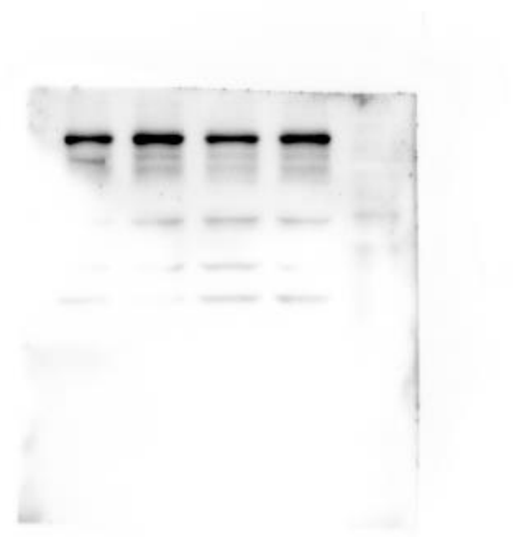

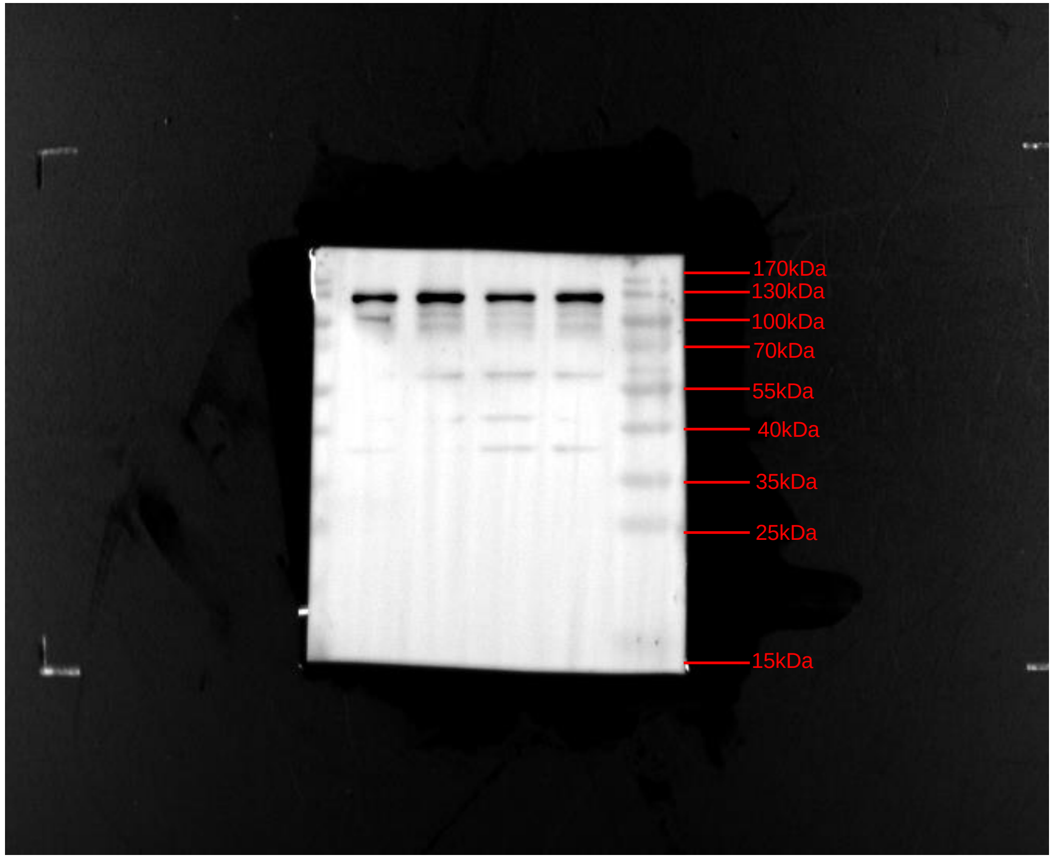

Figure 7h Vimentin RBE

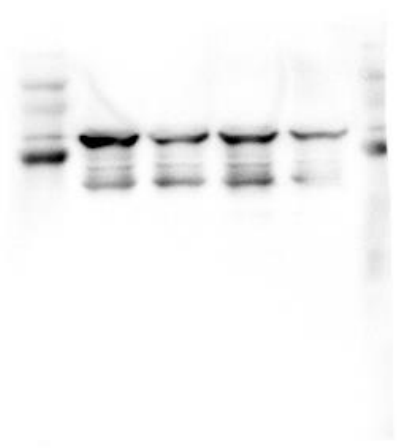

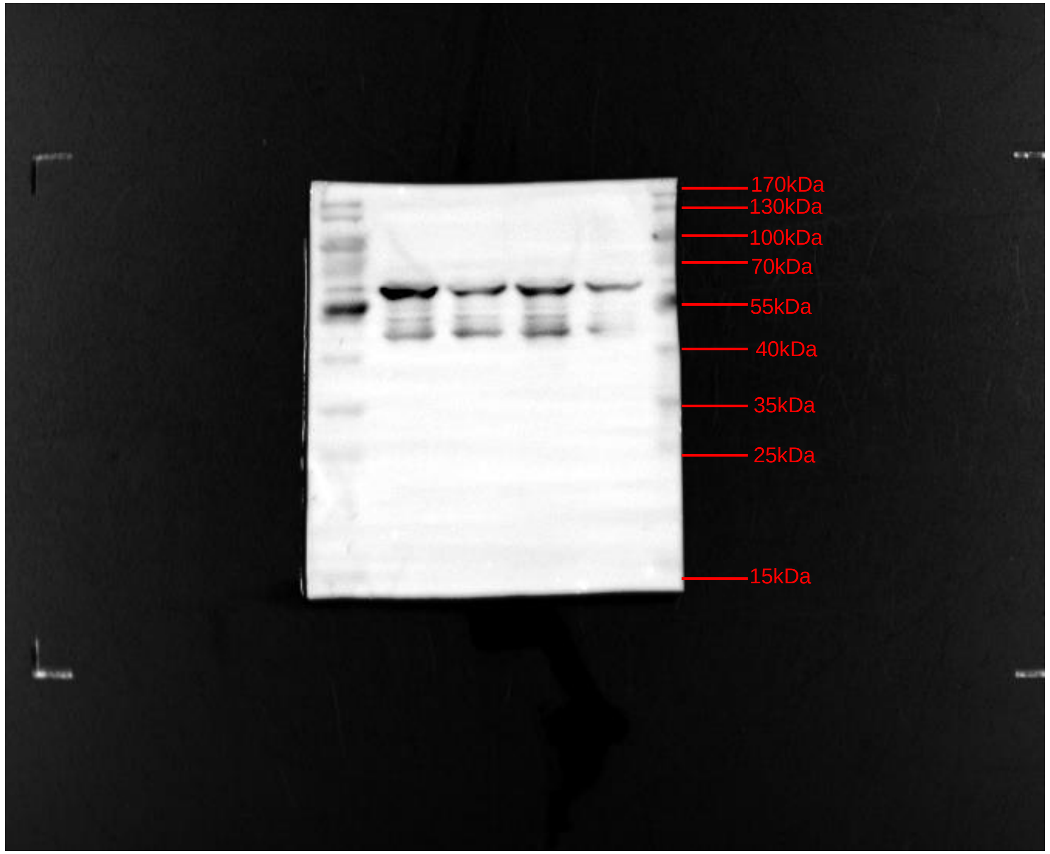

Figure 7h Transferrin RBE

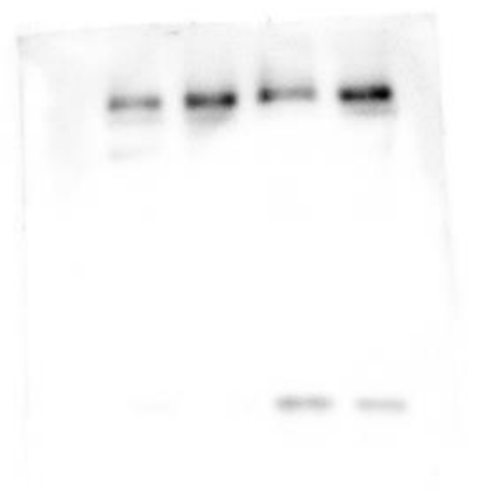

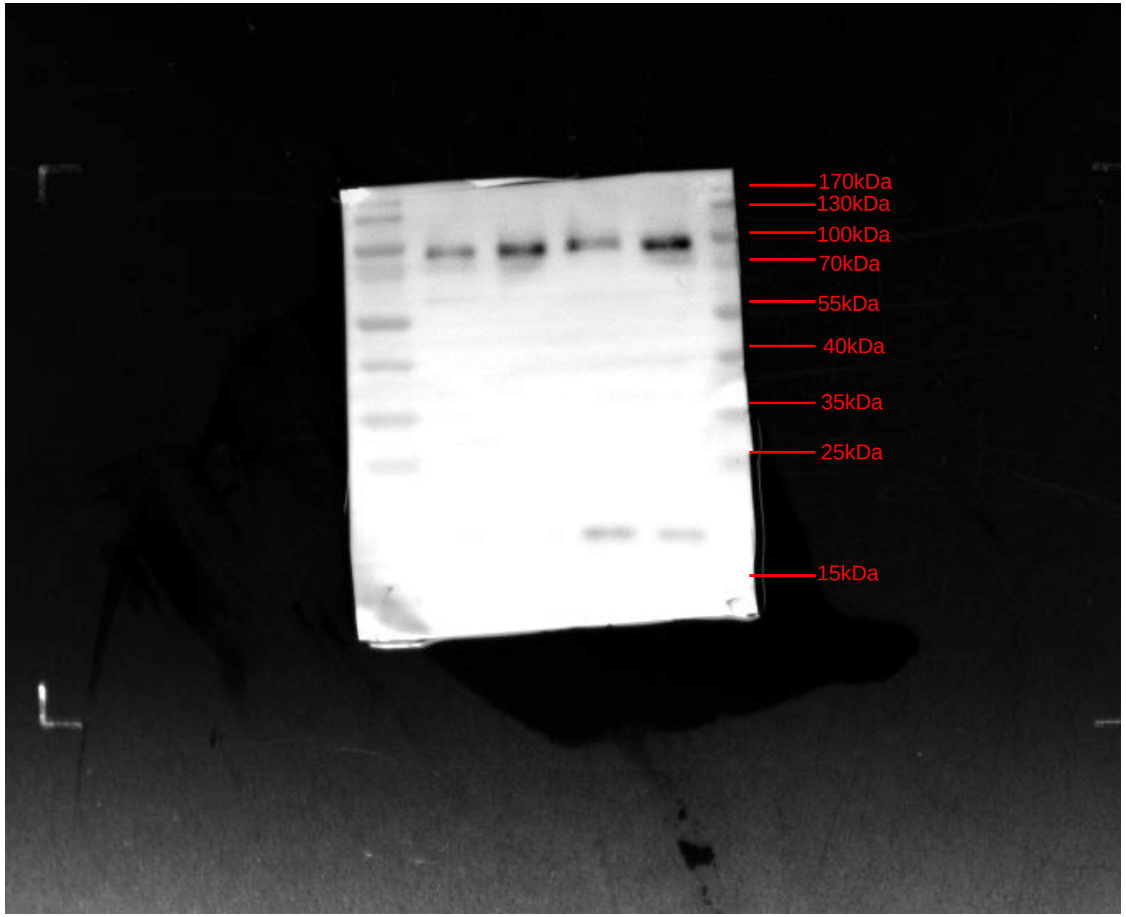

Figure 7h SLC40A1 RBE

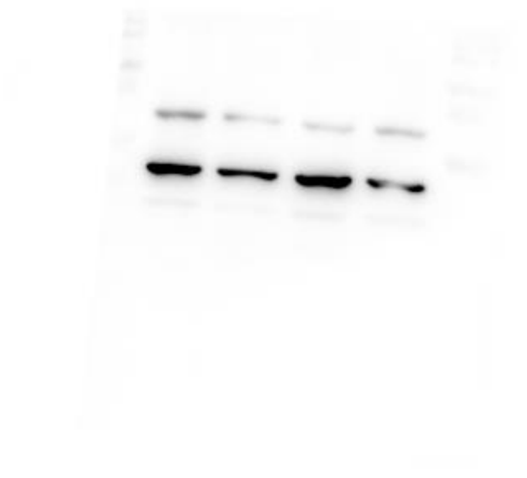

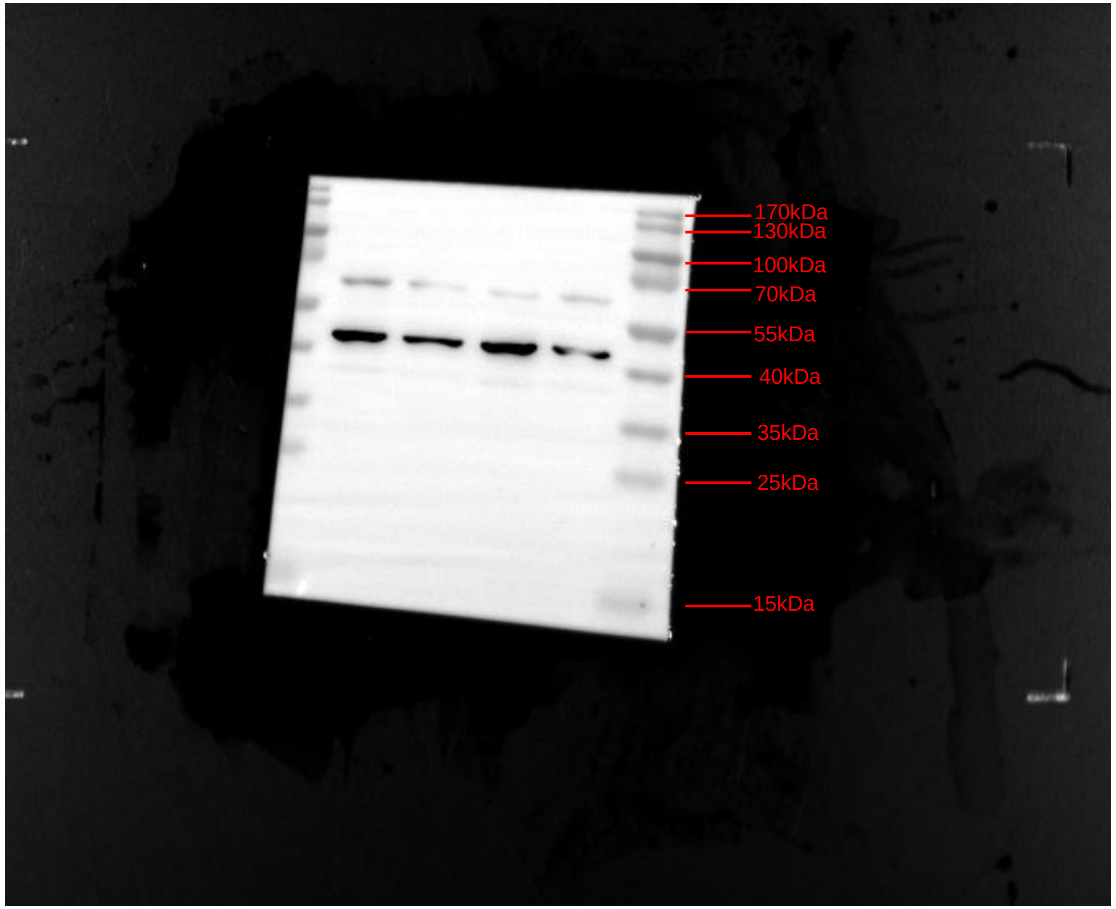

Figure 7h SLC7A11 RBE

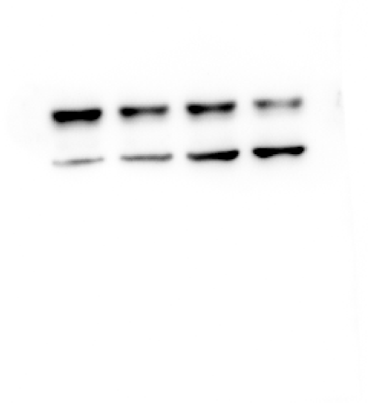

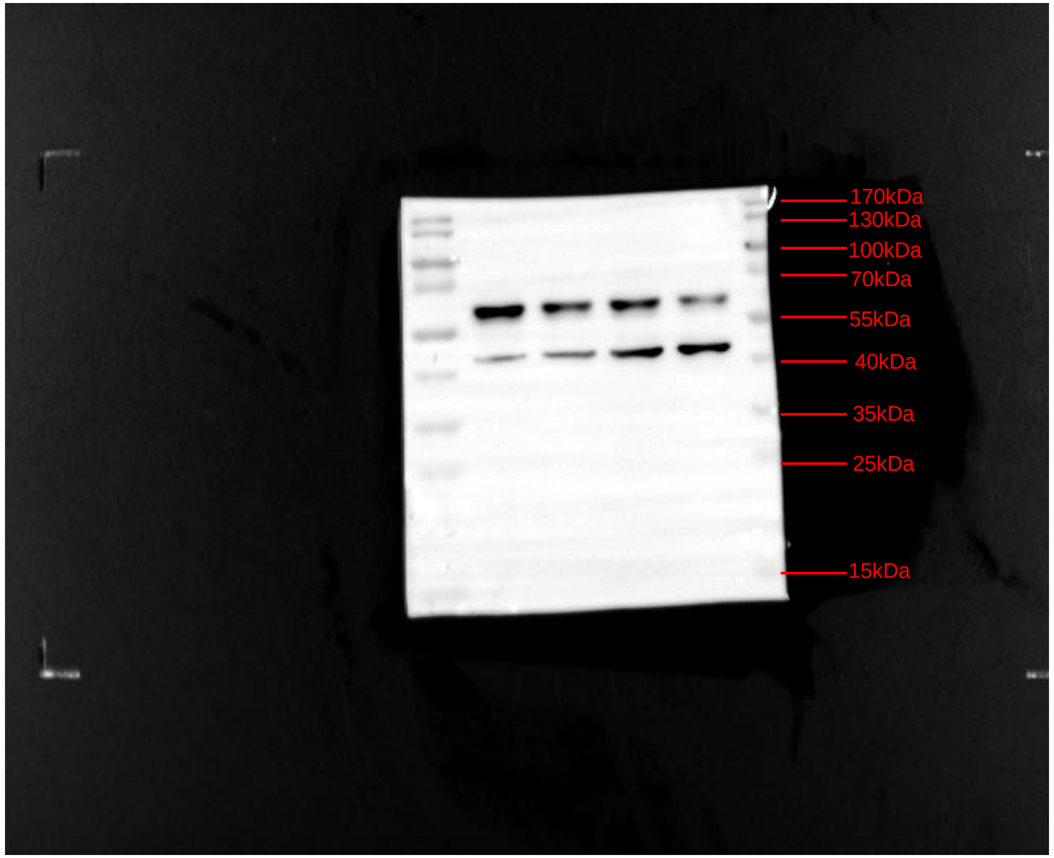

Figure 7h COX2 RBE

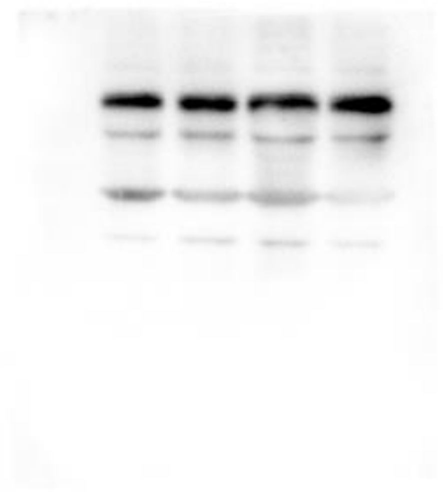

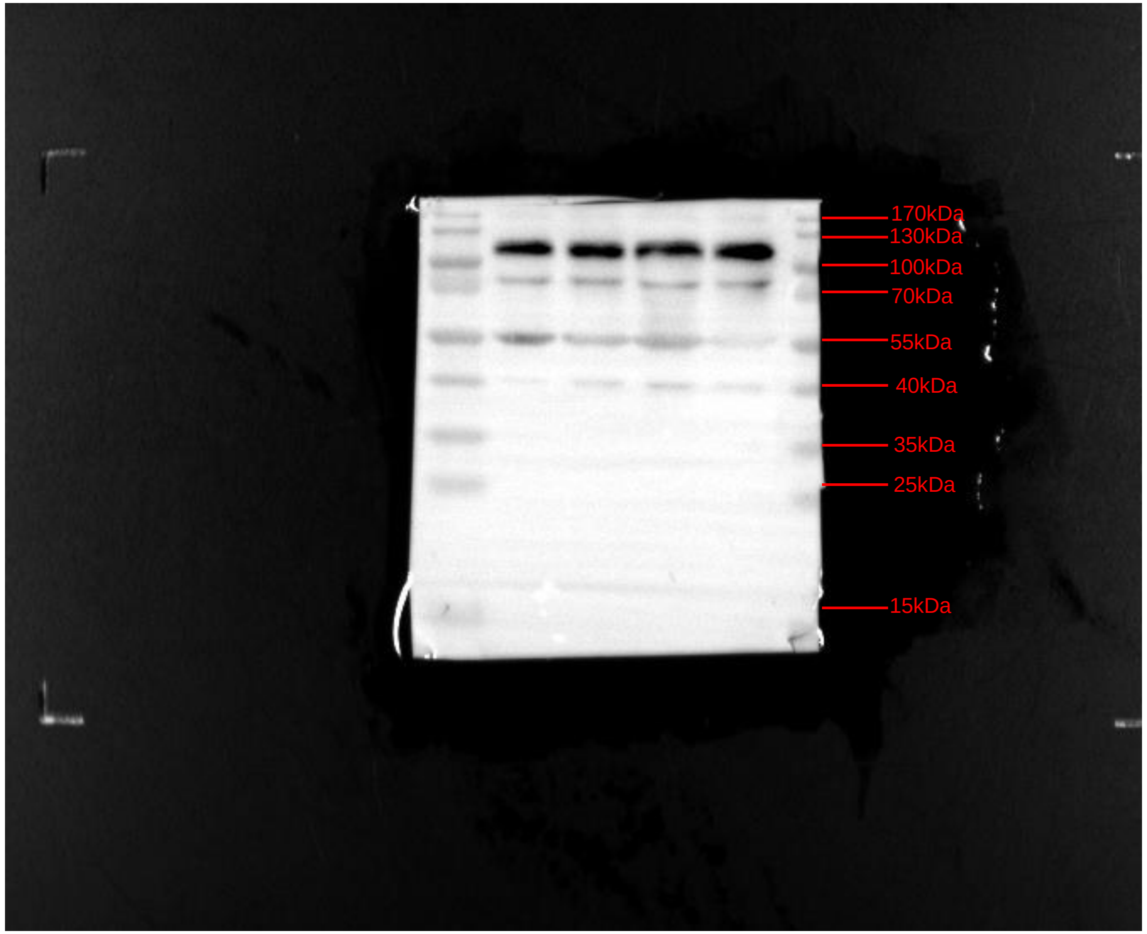

Figure 7h GPX4 RBE

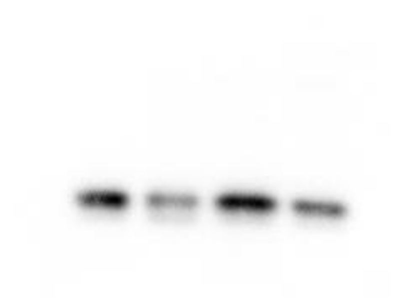

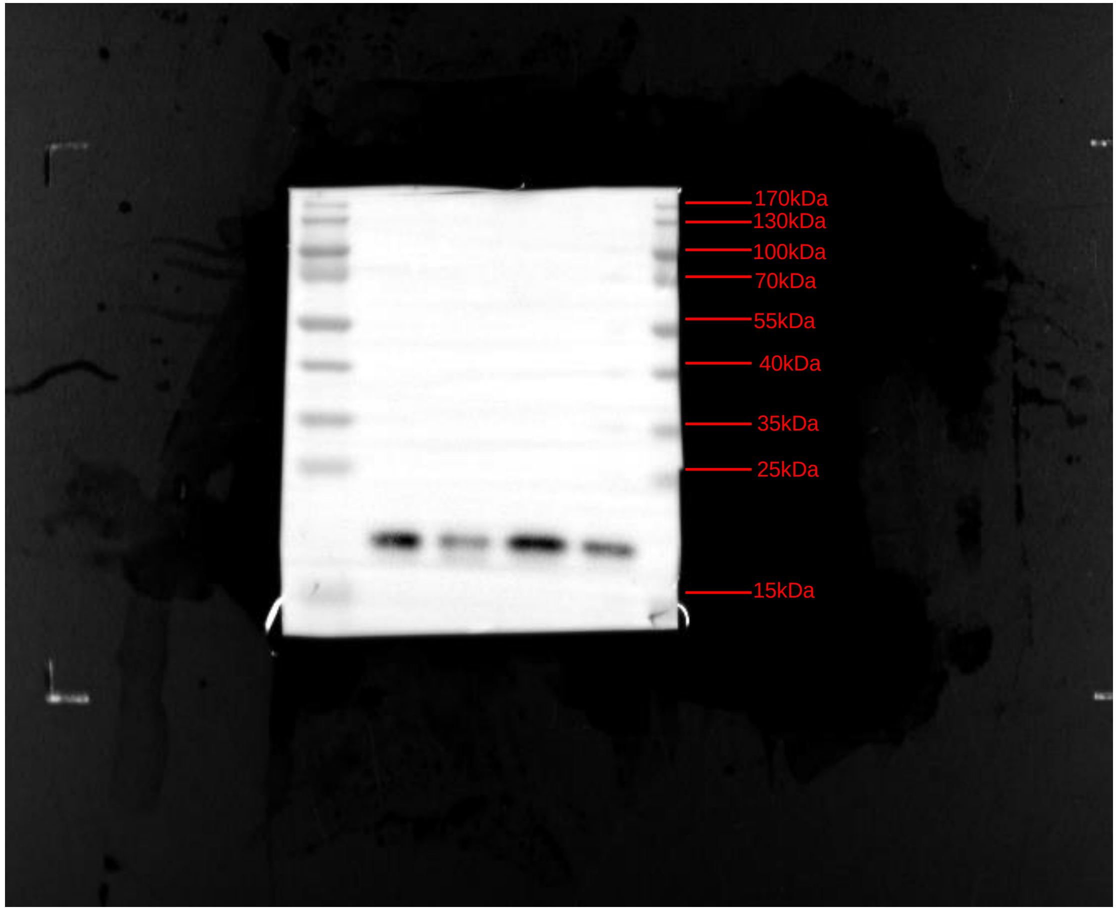

Figure 7h GPADH RBE

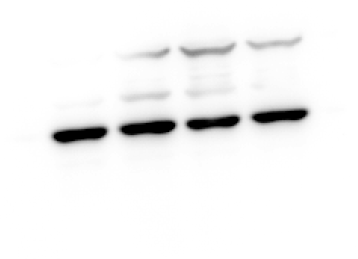

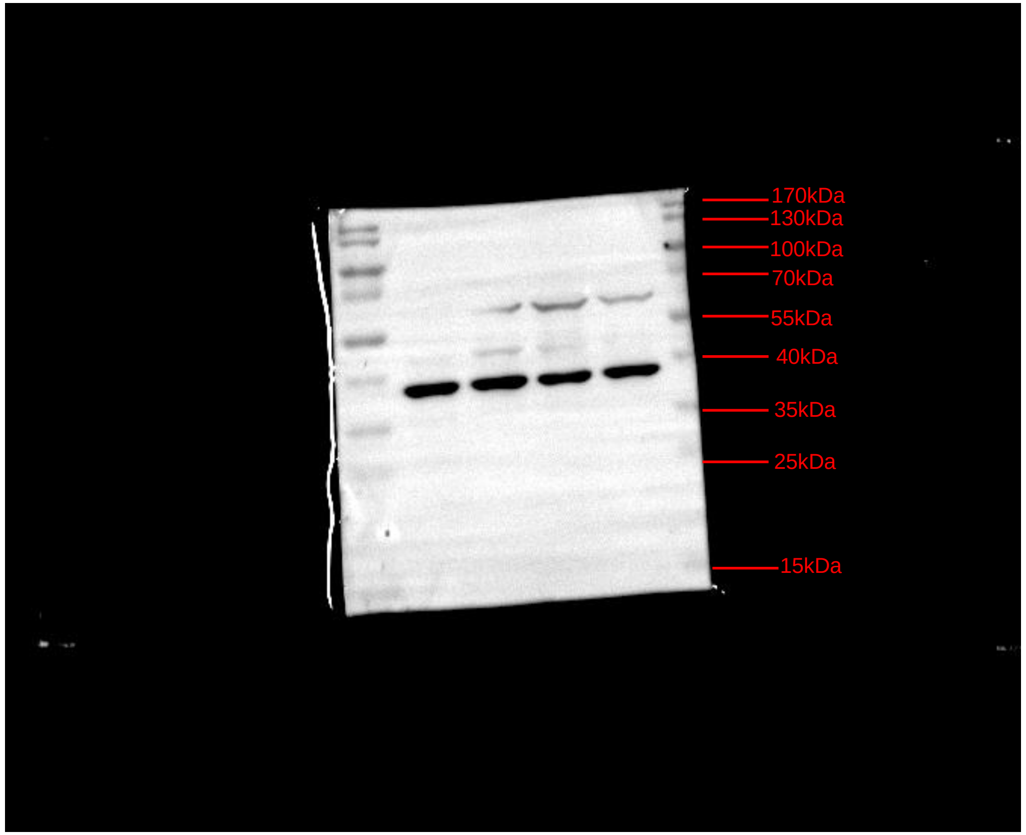

Figure 8h GPX4

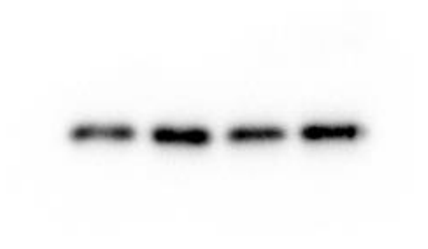

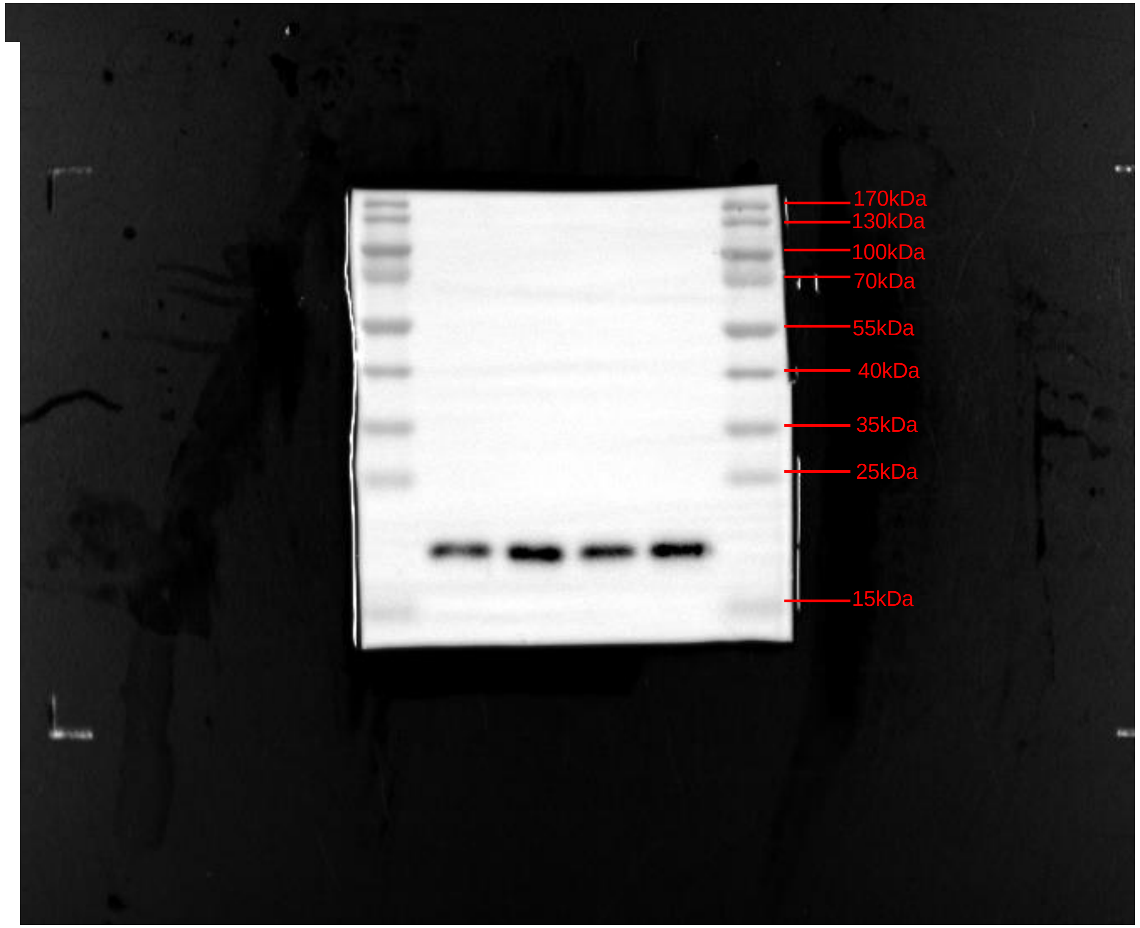

Figure 8h GAPDH

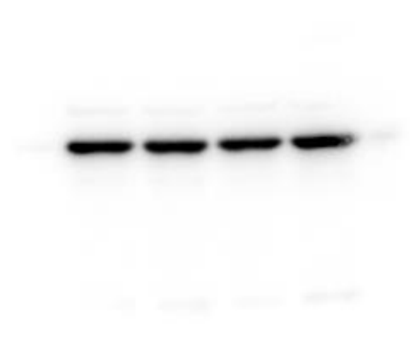

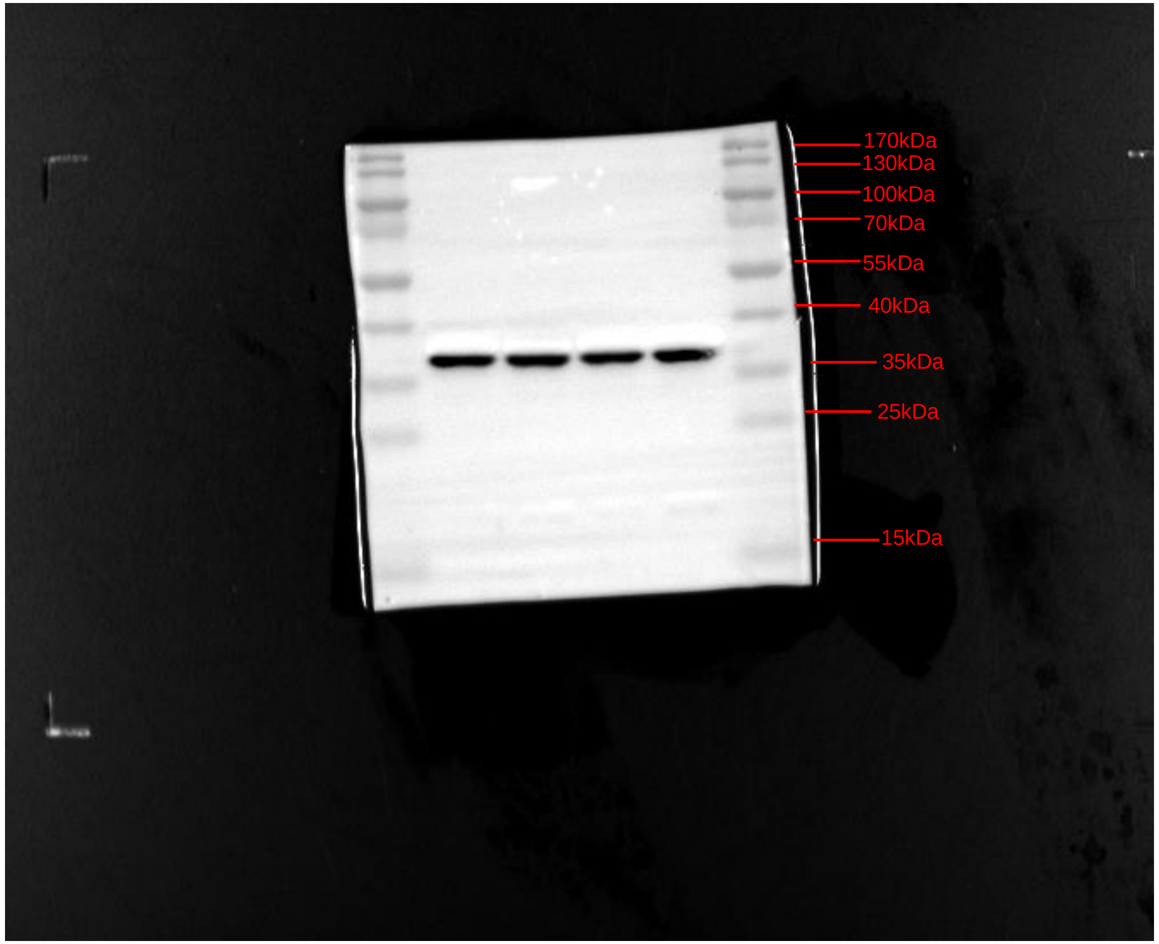

Supplement: Supplementary file 6 — Supplemental Material [file 41419_2022_5412_MOESM6_ESM.pdf]
