# Supplementary material for: JUND/linc00976 promotes cholangiocarcinoma progression and metastasis, inhibits ferroptosis by regulating the miR-3202/GPX4 axis
Source: Cell Death Dis. 2022 Nov 18;13(11):967. doi: 10.1038/s41419-022-05412-5 (PMC9674662; doi:10.1038/s41419-022-05412-5)
Supplement: Supplementary file 10 — Certificate_of_editing [file 41419_2022_5412_MOESM10_ESM.pdf]

CERTIFICATE OF ENGLISH EDITING

This document certifies that the paper listed below has been edited to ensure that the language is clear and free of errors. The edit was performed by professional editors at Editage, a division of Cactus Communications, in cooperation with Taylor & Francis Group. The intent of the author's message was not altered in any way during the editing process. The quality of the edit has been guaranteed, with the assumption that our suggested changes have been accepted and have not been further altered without the knowledge of our editors.

Title

JUND/linc00976 promotes cholangiocarcinoma progression and metastasis and inhibits ferroptosis by regulating the miR-3202/GPX4 axis

Authors

Lei Shan1,2#, Zhixue Zhang1,2#, Zhirui Zeng1,2#, Wenpeng Cao3#, Qianting Tian2, Bangming Jin1,2, Tuo Zhang1,2, Yingming Wu1,2, Dahuan Li5, Chujiao Hu6, Jinzhi Lan2, Jinjuan Zhang2,4*, Tengxiang Chen1,2*

Order No.

FIENZ_3

EDITINGSERVICES
Supporting Taylor & Francis authors

Signature

Vikas Narang

Vikas Narang,
Chief Operating Officer,
Editage

DNV·GL ISO/IEC 27001

editage

Date of Issue
August 01, 2022
